# Supplementary material for: A Comprehensive Metabolomic Analysis of Volatile and Non-Volatile Compounds in Folium Artemisia argyi Tea from Different Harvest Times
Source: Foods. 2025 Feb 28;14(5):843. doi: 10.3390/foods14050843 (PMC11899400; doi:10.3390/foods14050843)
Supplement: Supplementary file 1 [file foods-14-00843-s001.zip › Figure S1 PLS-DA analysis of UPLC-MSMS for FAA tea harvested at four different times.pdf]

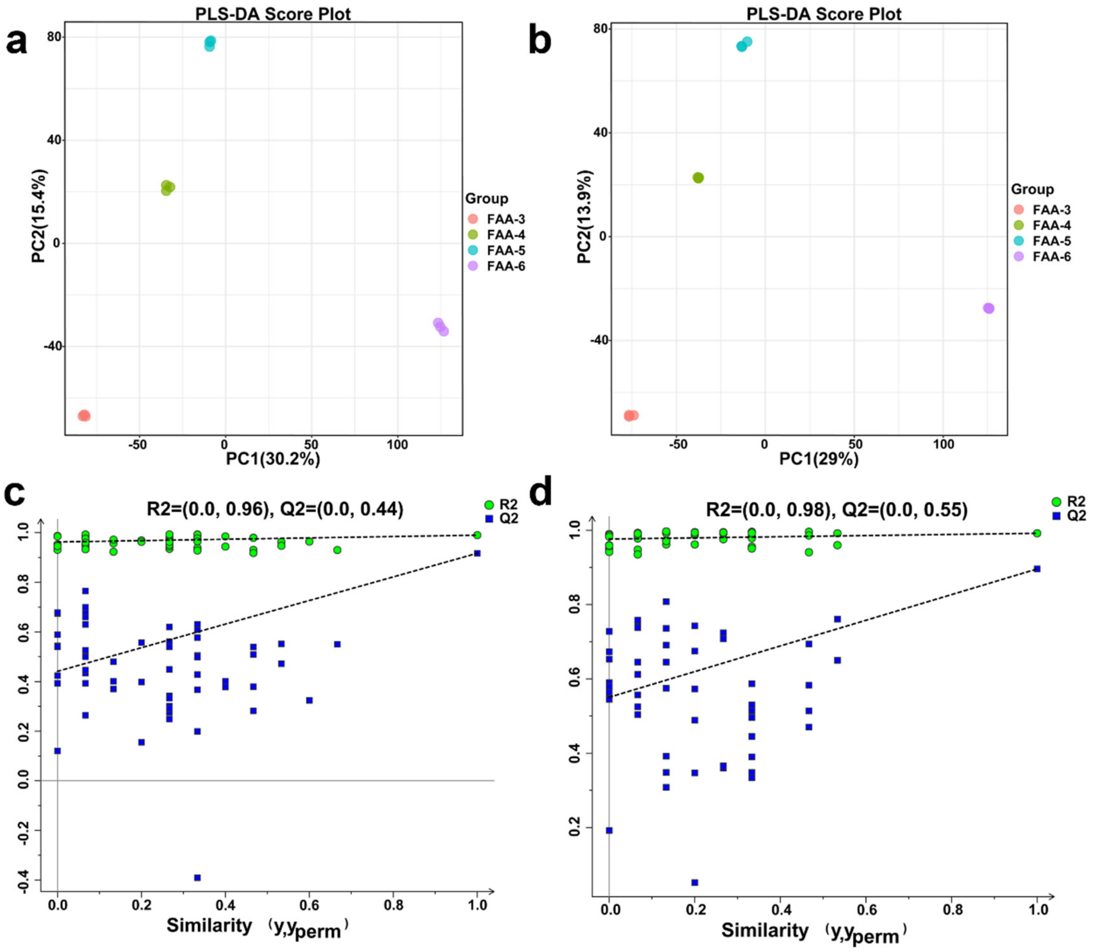

Figure S1 PLS-DA analysis of UPLC-MS/MS for FAA tea harvested at four different times. (a-b) PLS-DA score plots in positive (+) and negative (−) ion modes; (c-d) PLS-DA permutation test in positive (+) and negative (−) ion modes.
