# Supplementary material for: A Comprehensive Metabolomic Analysis of Volatile and Non-Volatile Compounds in Folium Artemisia argyi Tea from Different Harvest Times
Source: Foods. 2025 Feb 28;14(5):843. doi: 10.3390/foods14050843 (PMC11899400; doi:10.3390/foods14050843)
Supplement: Supplementary file 1 [file foods-14-00843-s001.zip › Figure S2 MS2 spectra of differential nonvolatile metabolites in FAA tea harvested at four different times.pdf]

MS² spectra of differential nonvolatile metabolites in FAA tea harvested at four different times

Naringenin

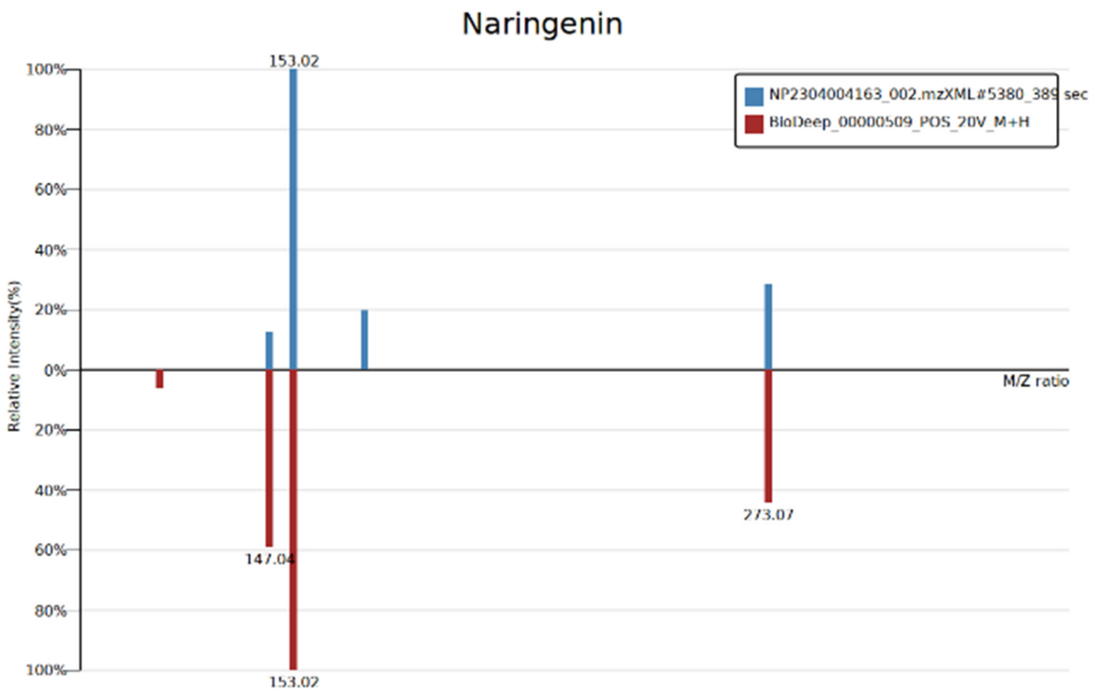

Fisetin

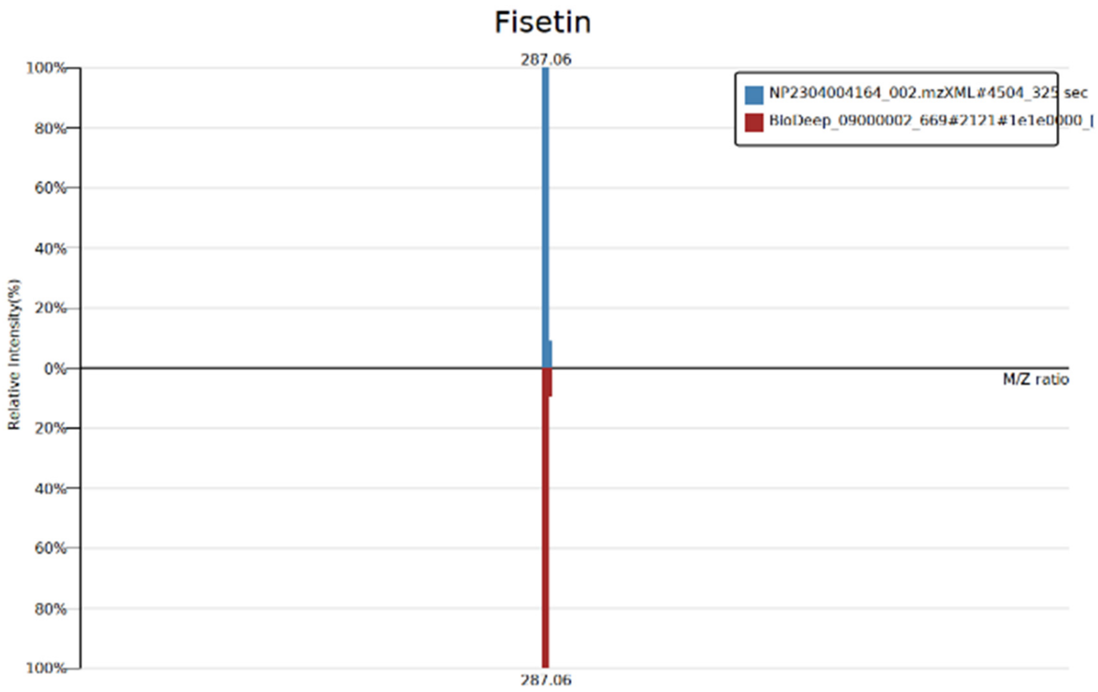

Hispidulin

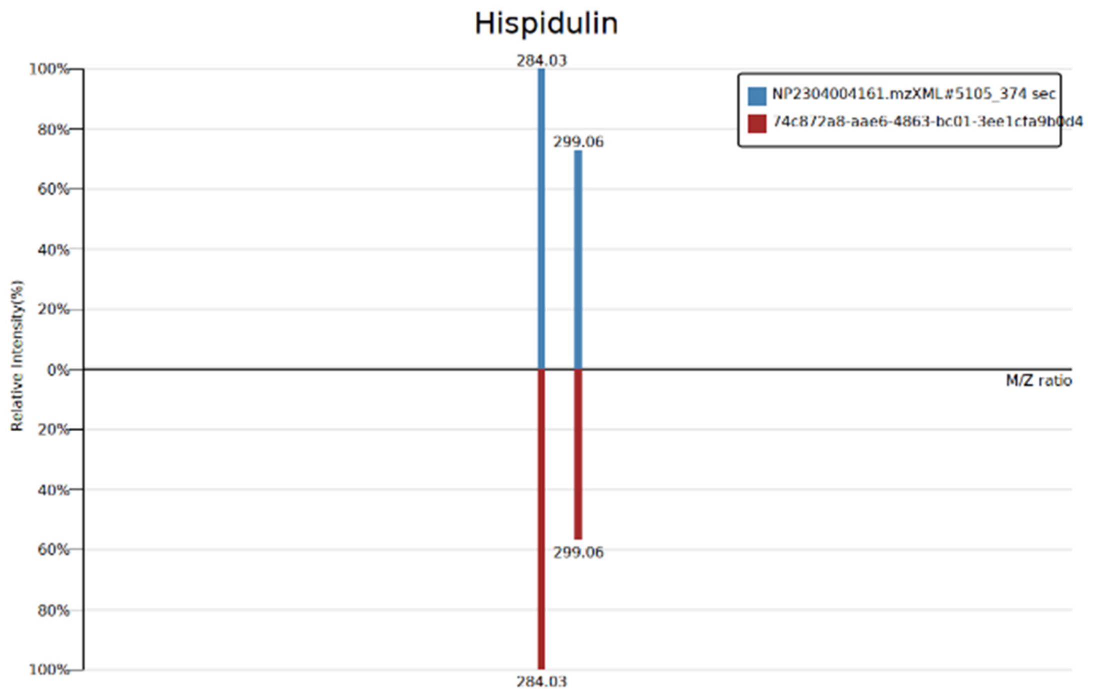

Diosmetin

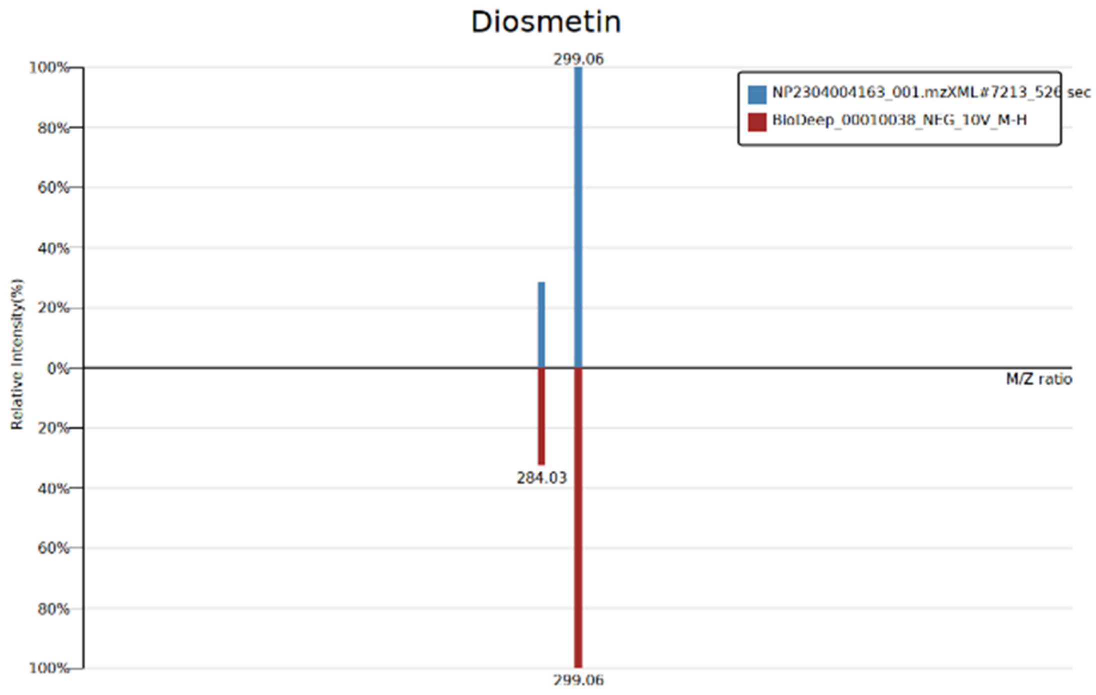

Taxifolin

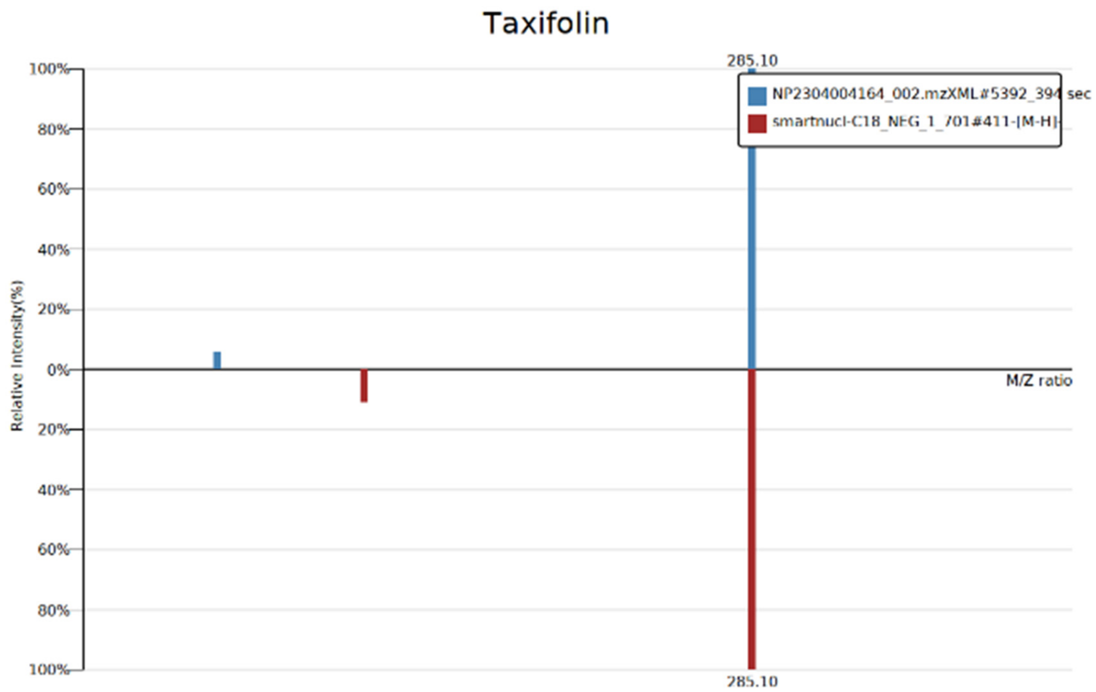

Isorhamnetin

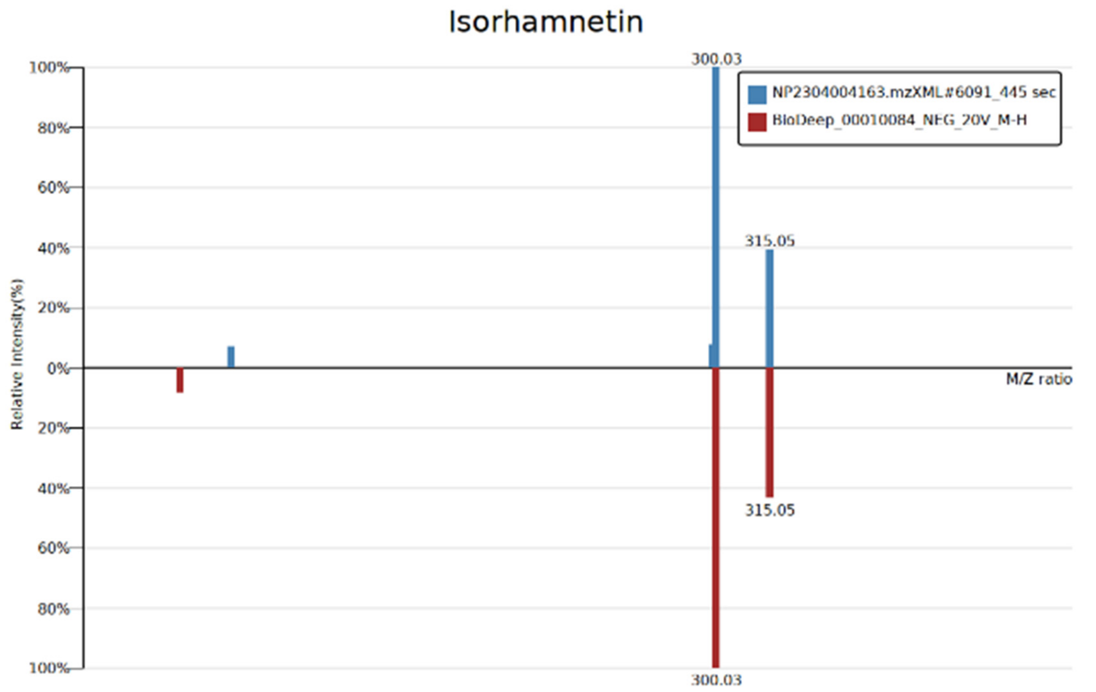

Rhamnetin

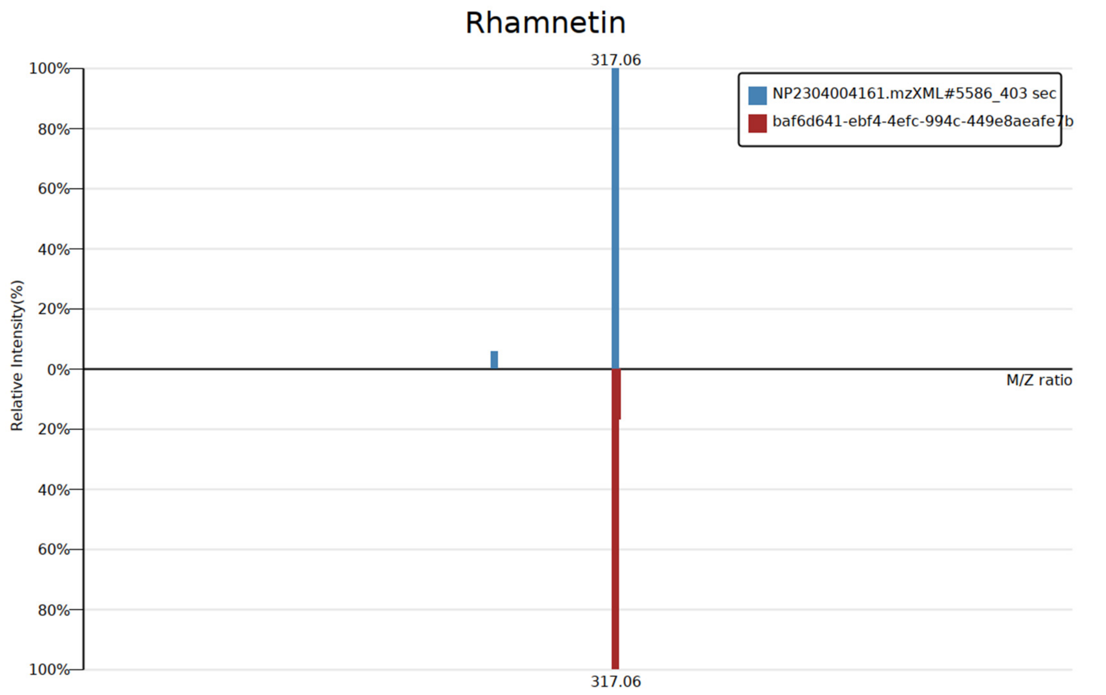

Homoeriodictyol

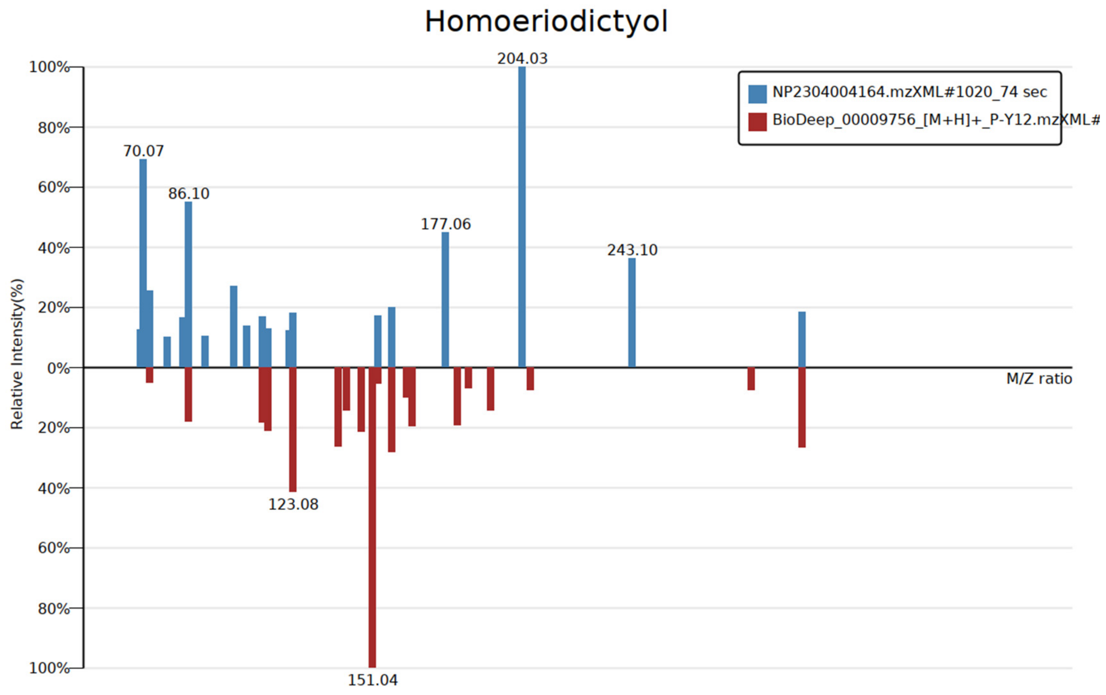

Isoquercitrin

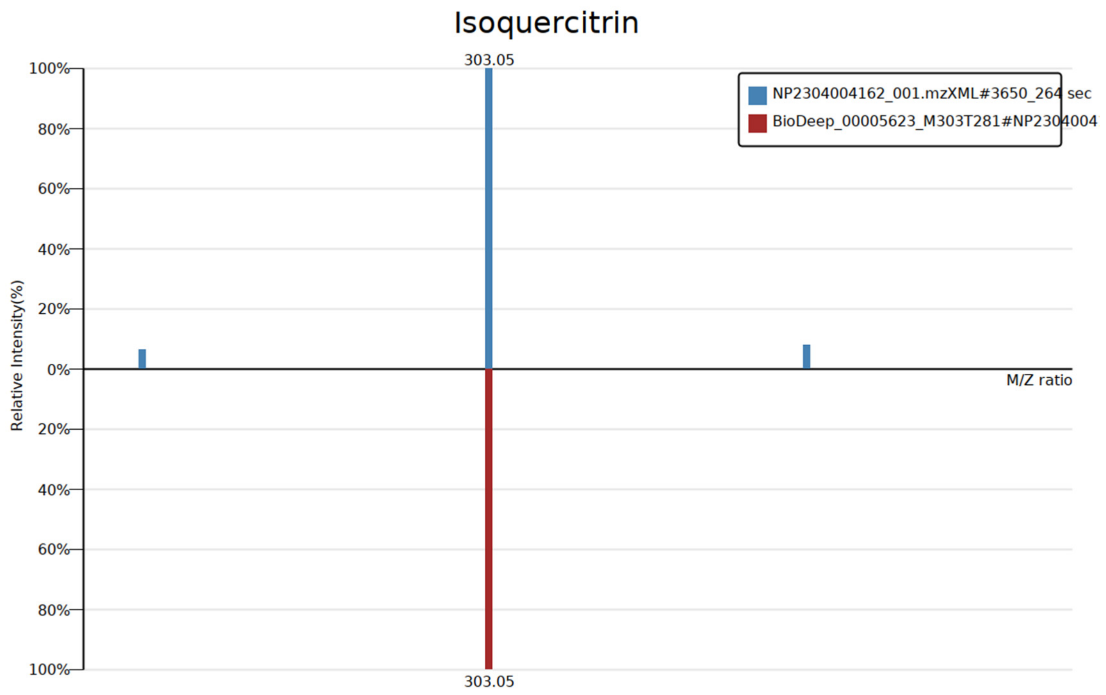

Naringin

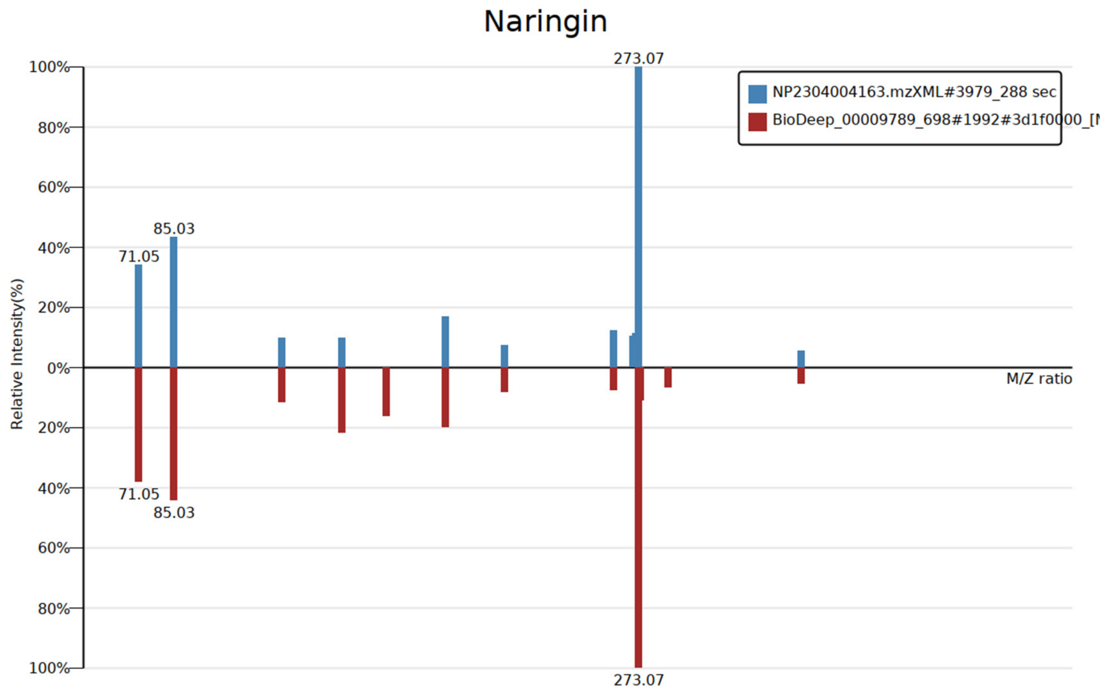

(-)-Epigallocatechin

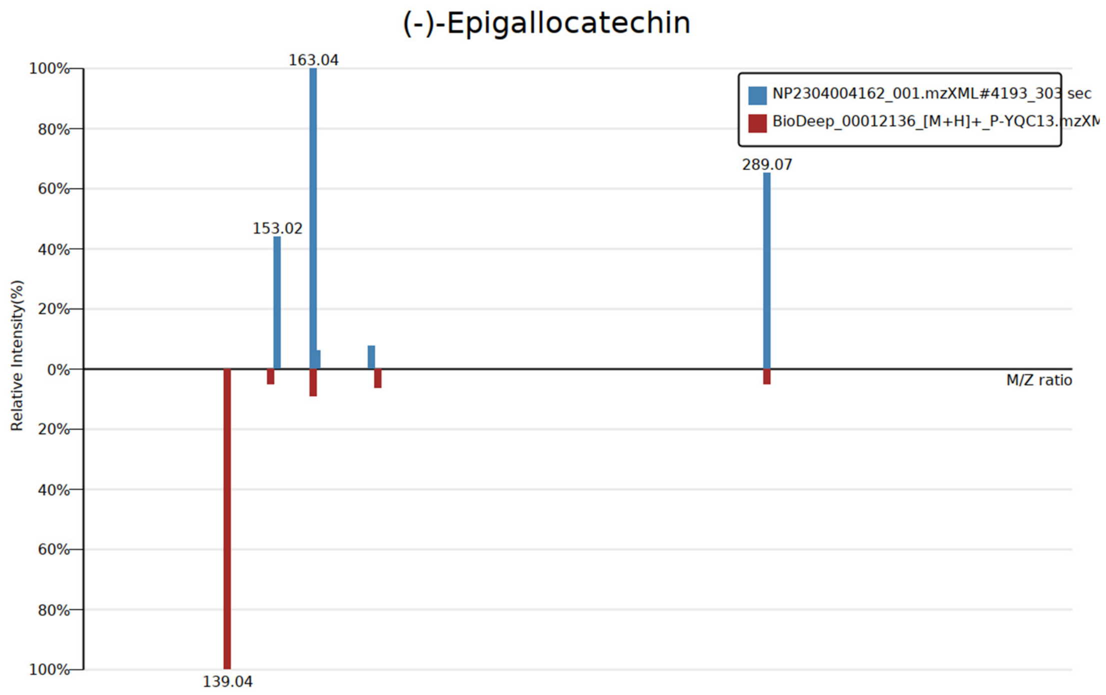

p-Hydroxyphenylacetic acid

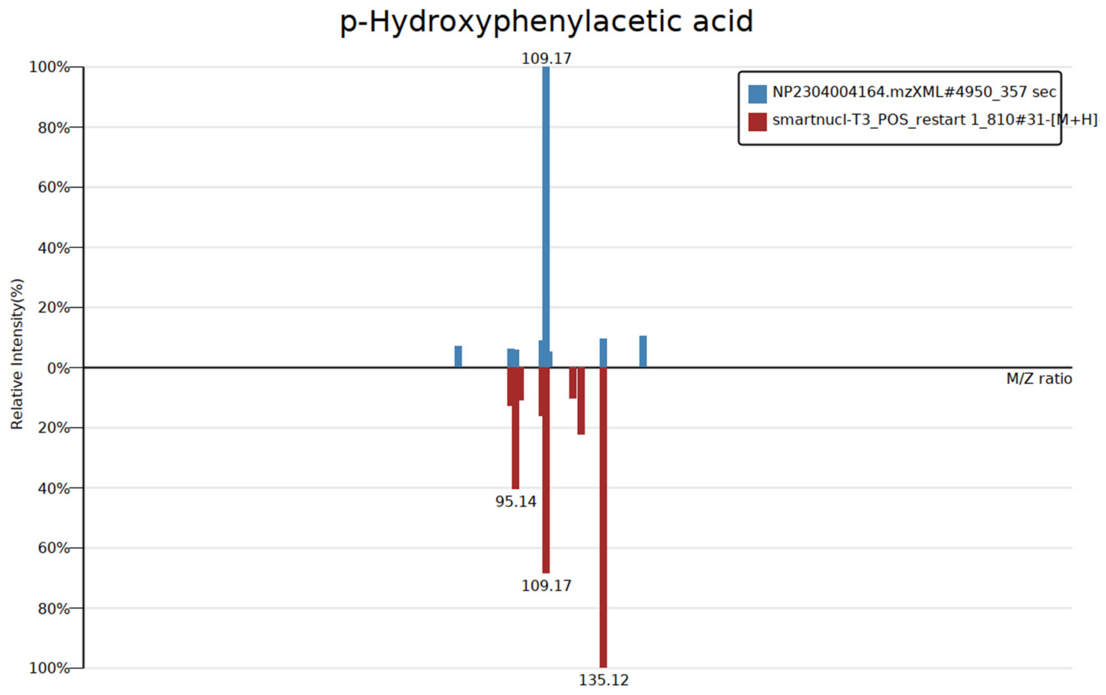

3-Hydroxyanthranilic acid

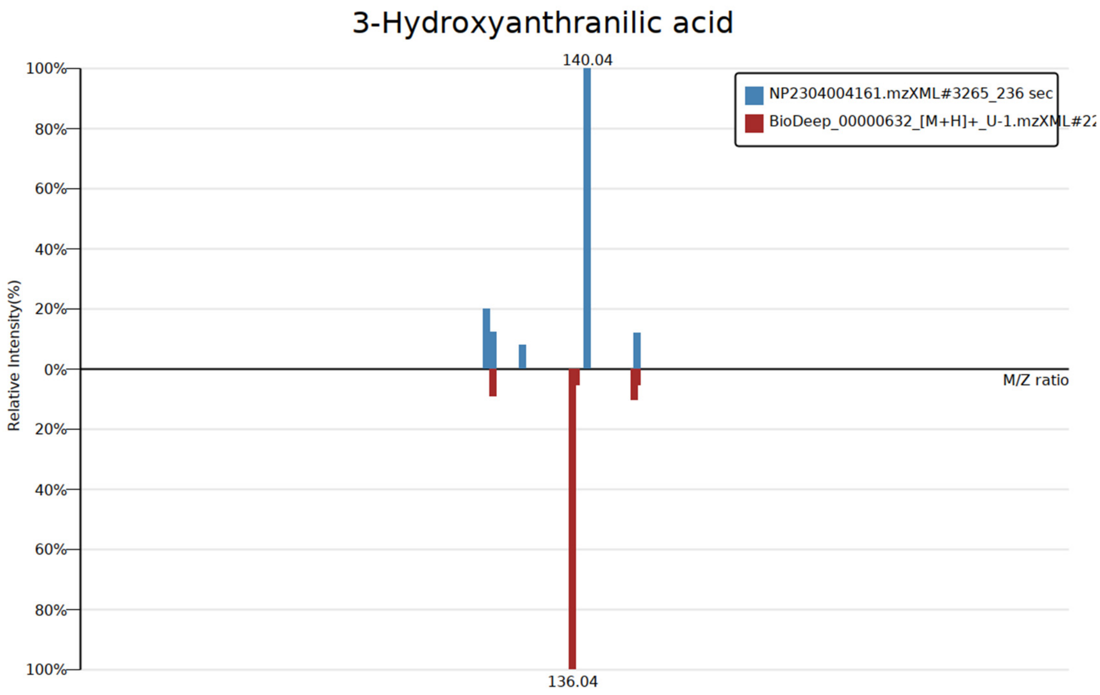

3-(2-Hydroxyphenyl)propanoic acid

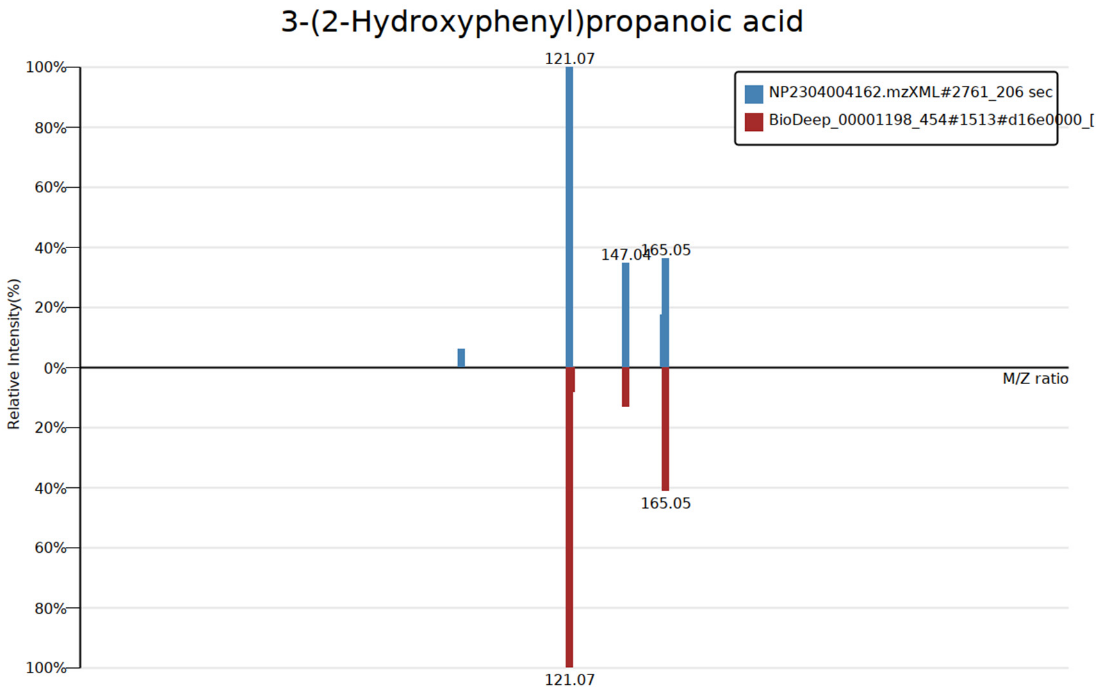

3,4-Dihydroxybenzeneacetic acid

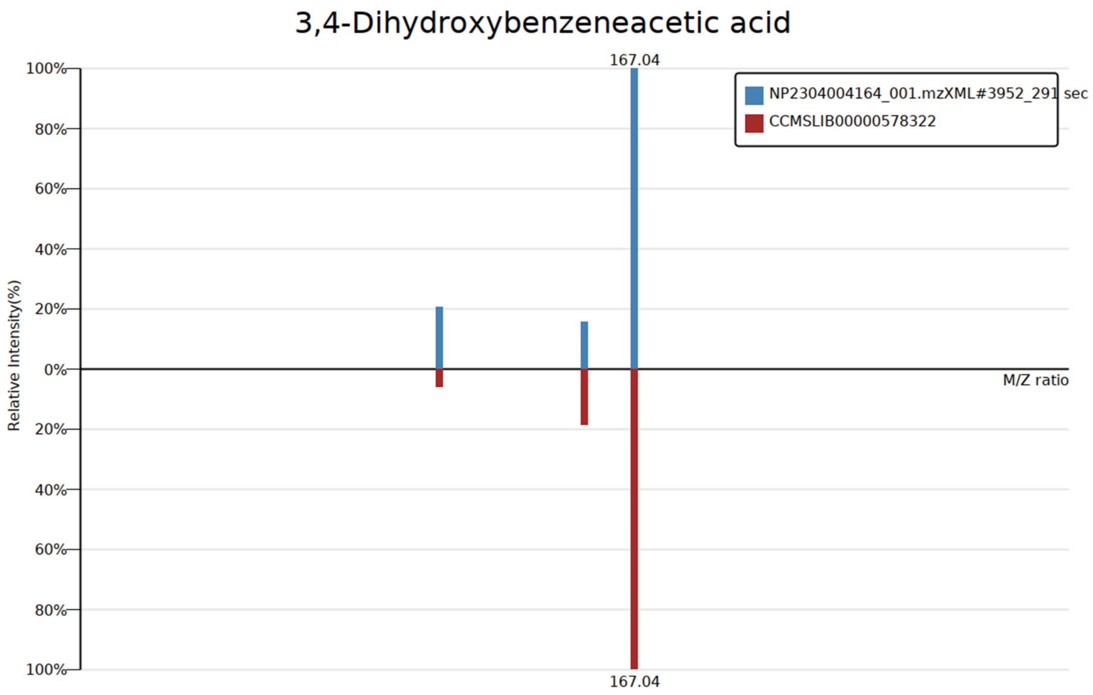

Gallic acid

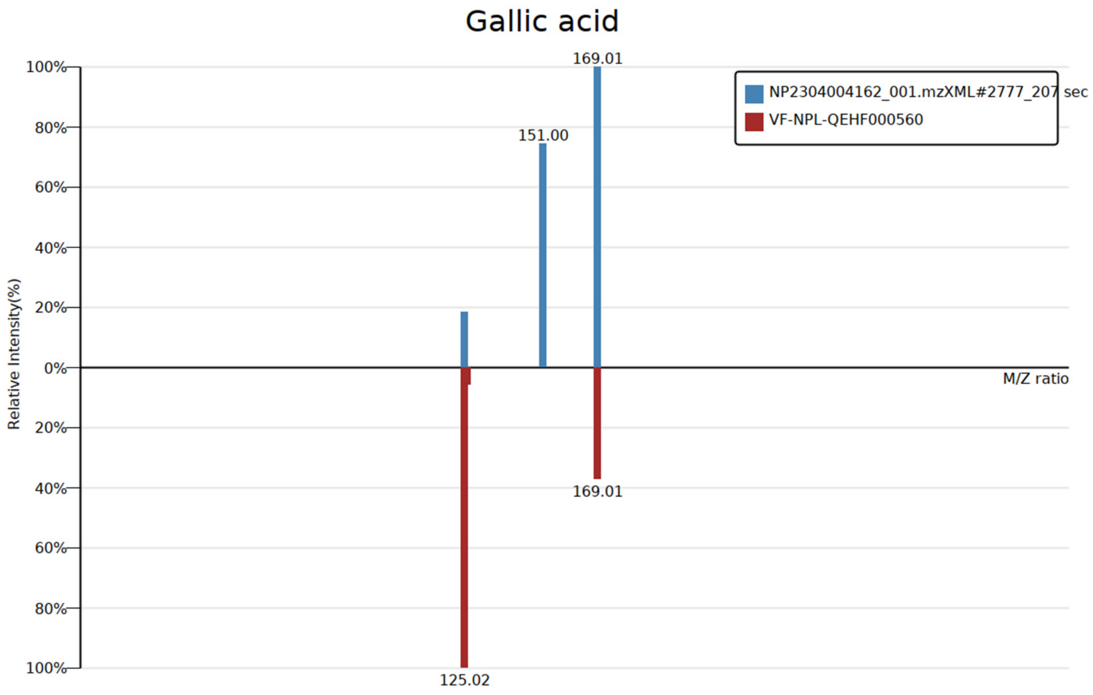

4-Hydroxyphenylpyruvic acid

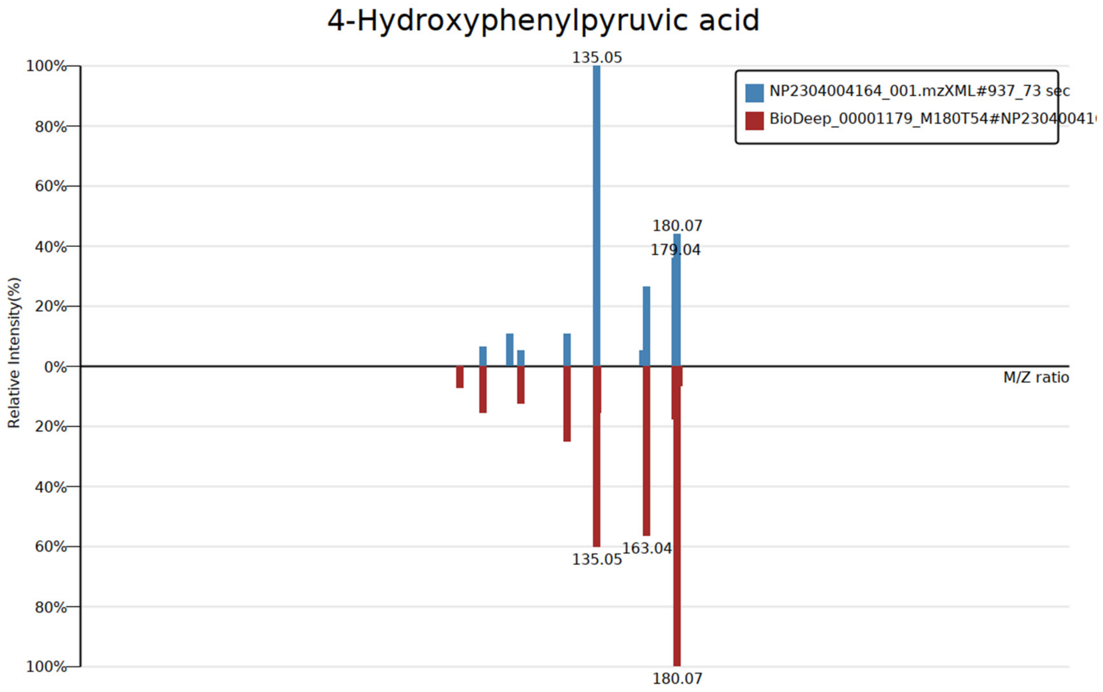

Hydroxyphenyllactic acid

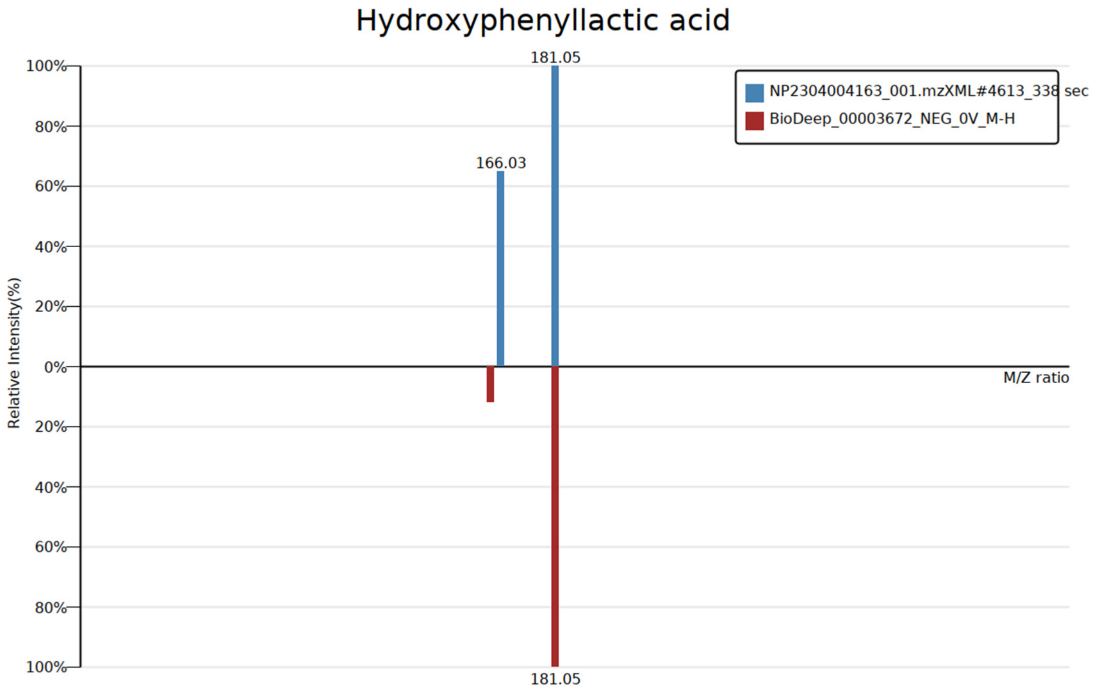

p-Coumaroyl quinic acid

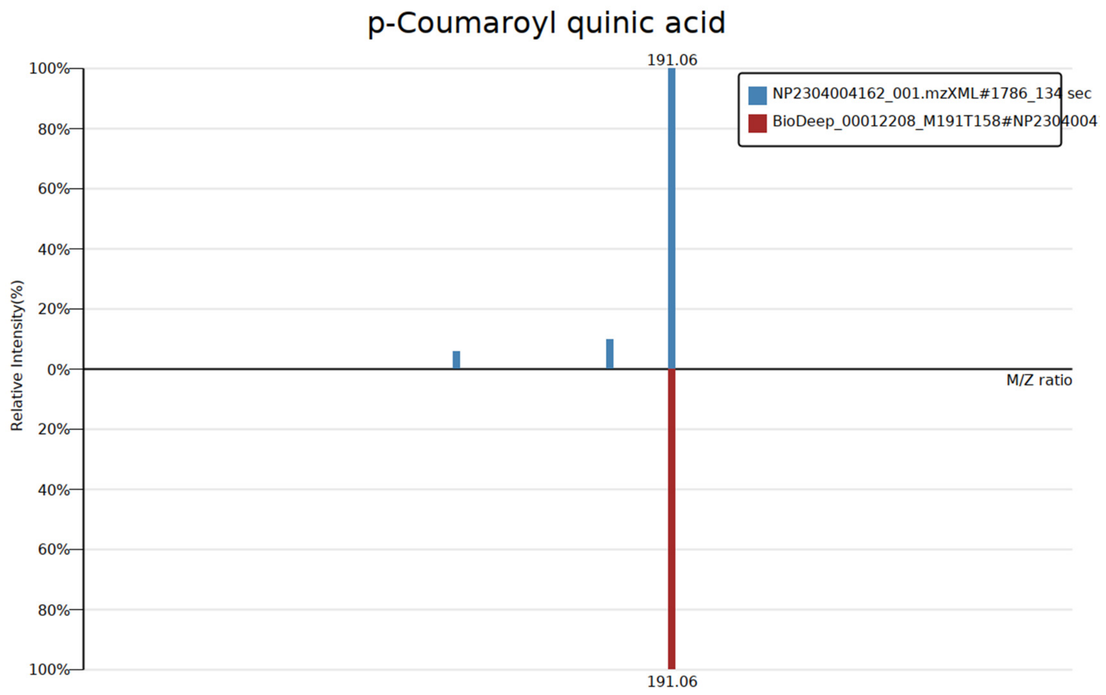

trans-Cinnamoyl beta-D-glucoside

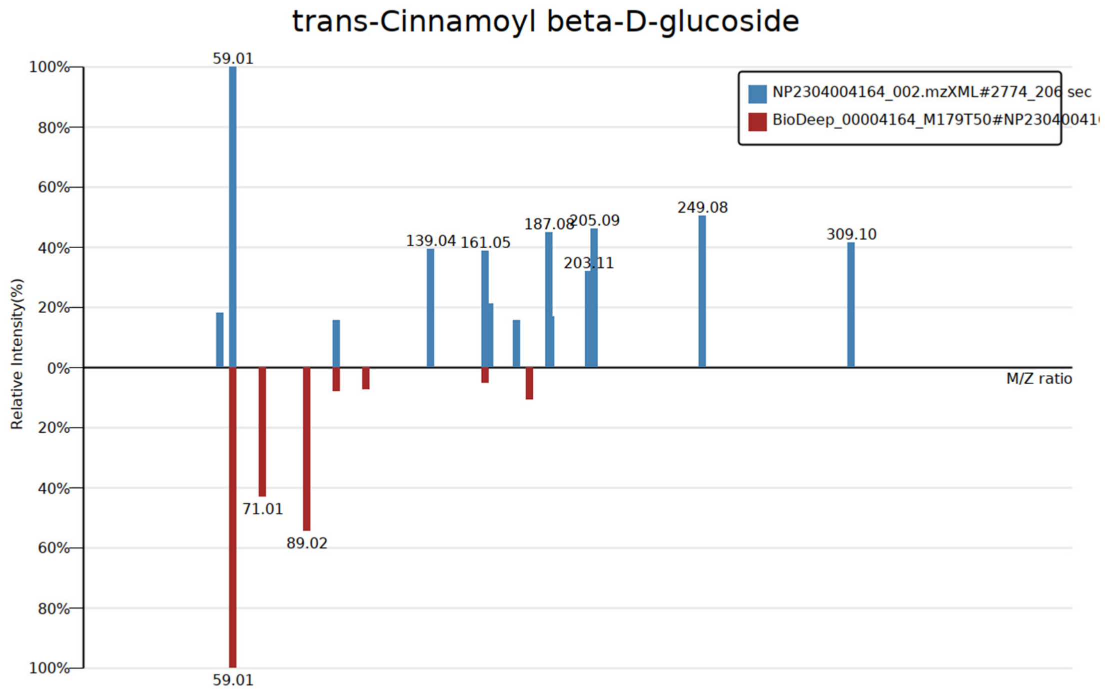

1-O-Vanilloyl-beta-D-glucose

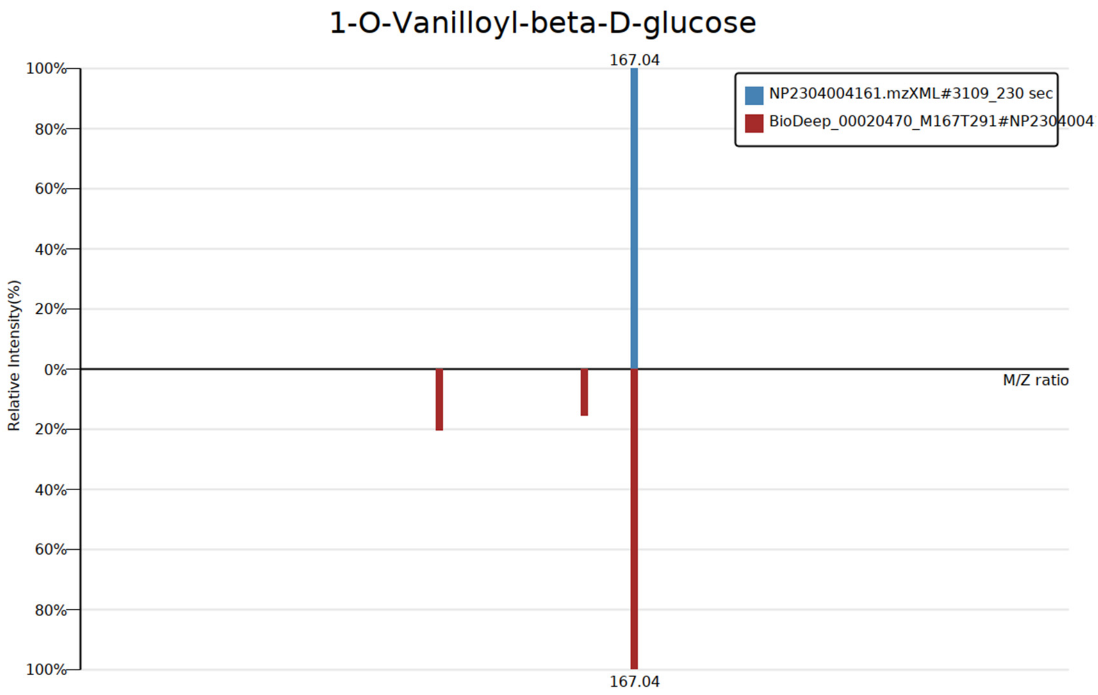

1-O-Feruloyl-beta-D-glucose

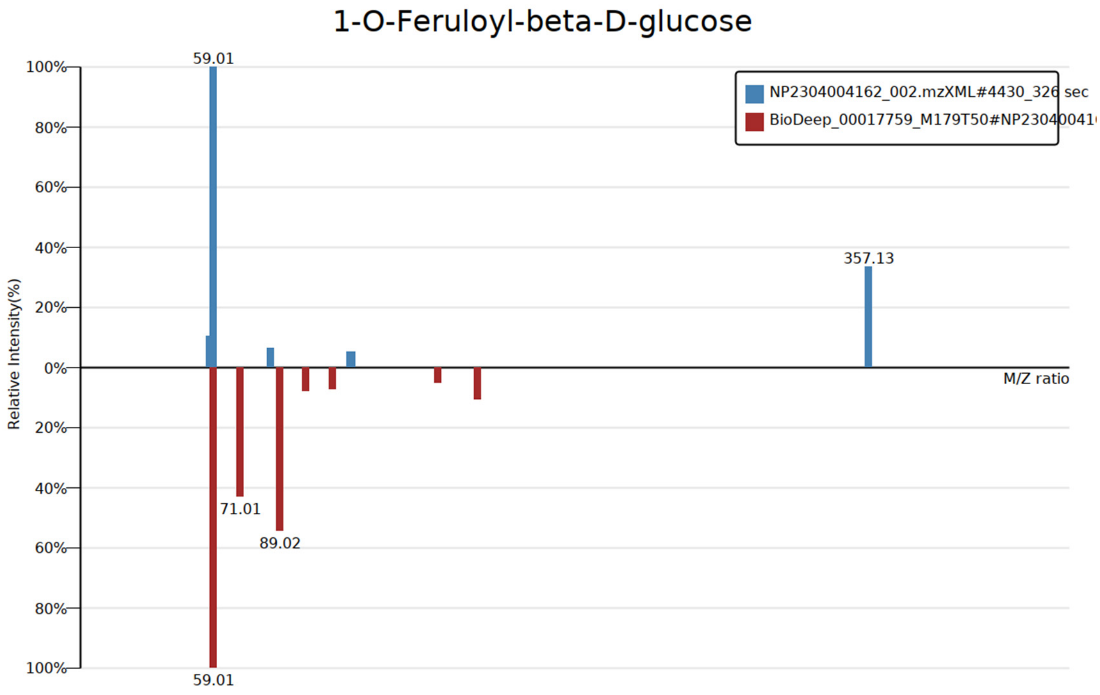

Hydroxykynurenine

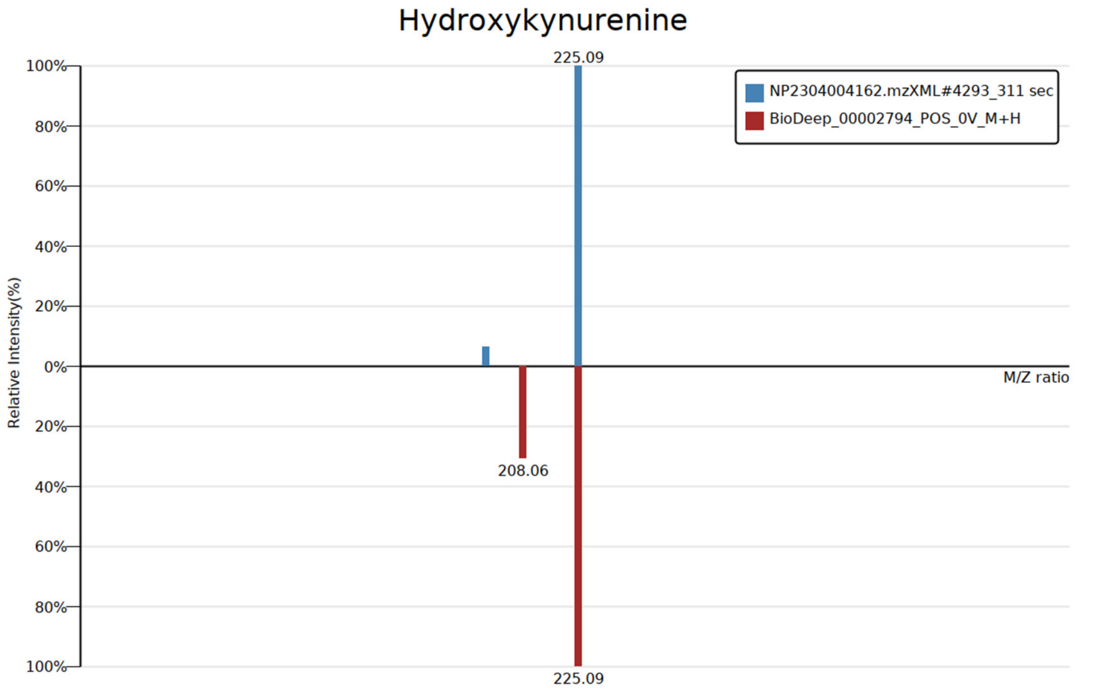

Prephenate

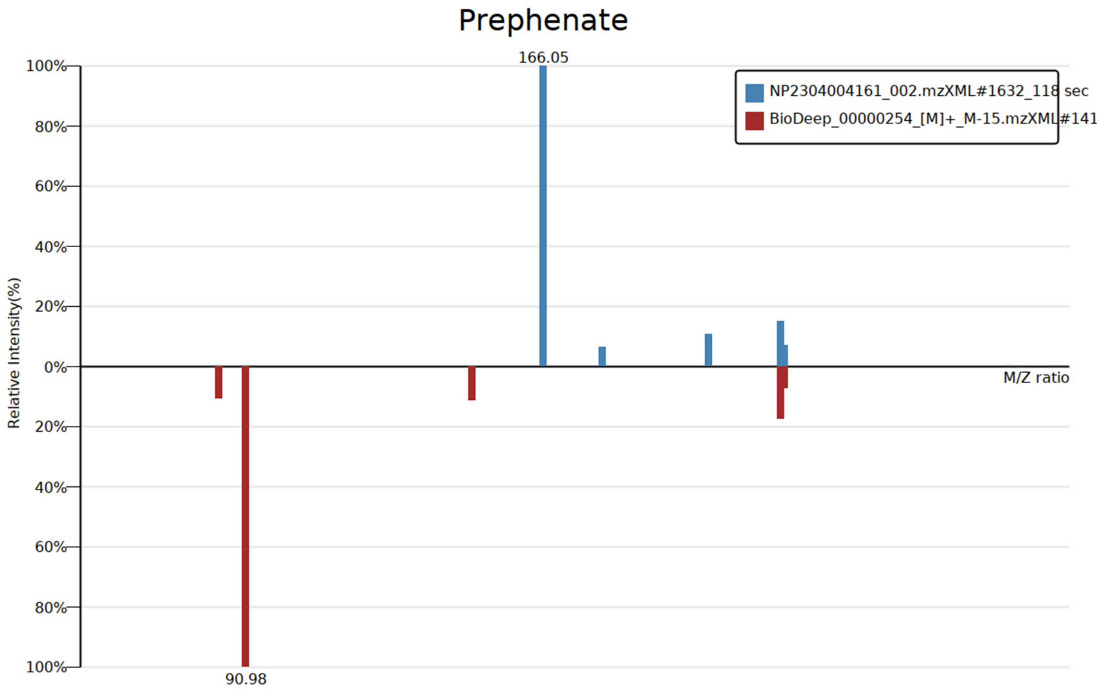

N-Succinyl-2-amino-6-ketopimelate

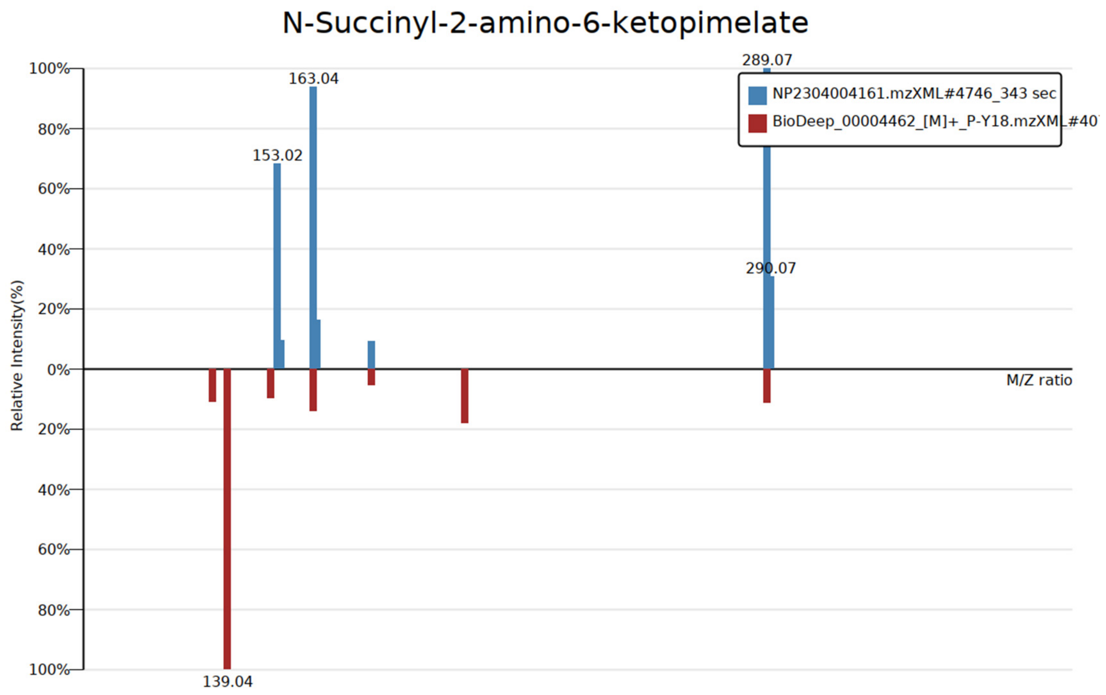

N-methyl-L-glutamic Acid

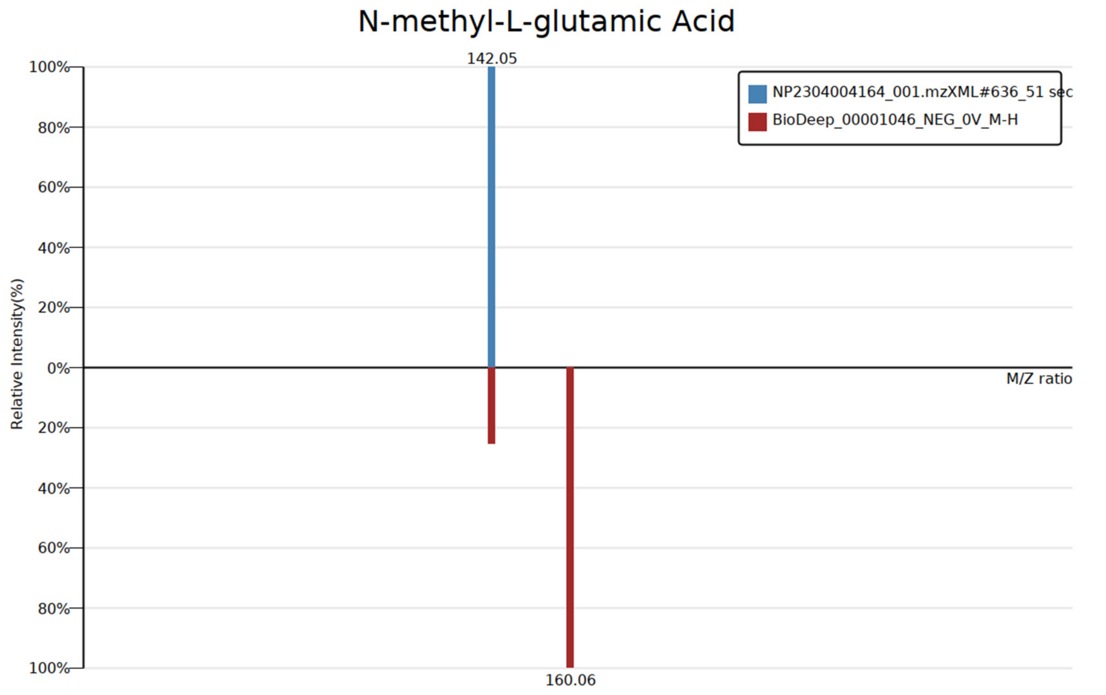

gamma-Aminobutyric acid

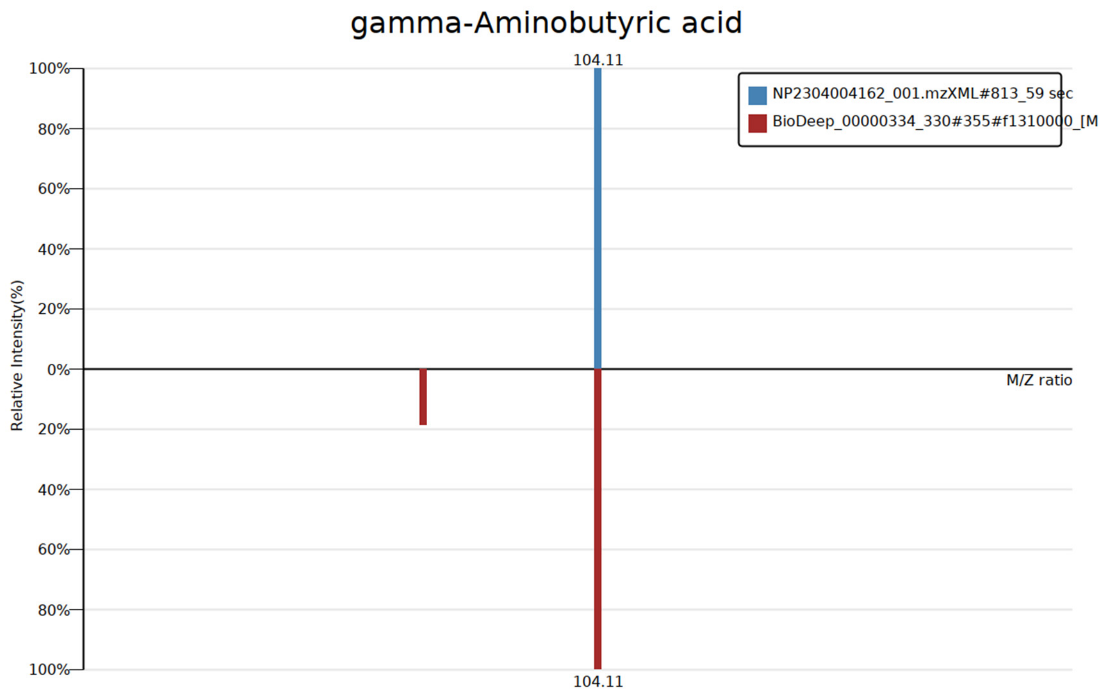

L-Proline

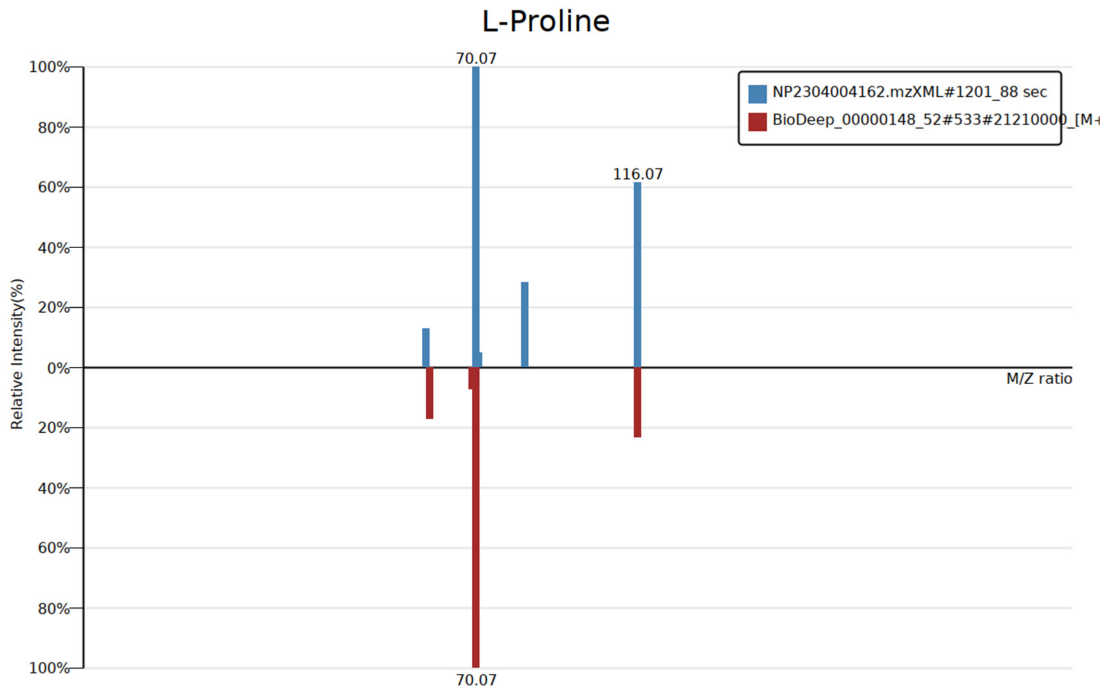

L-Valine

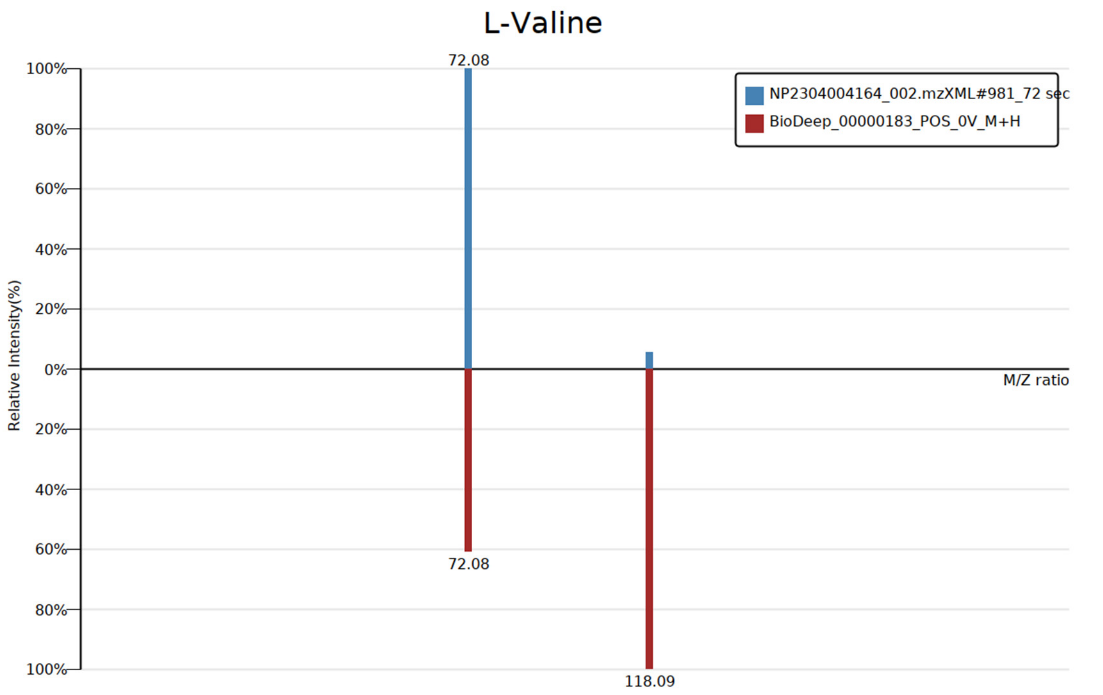

cis-4-Hydroxy-D-proline

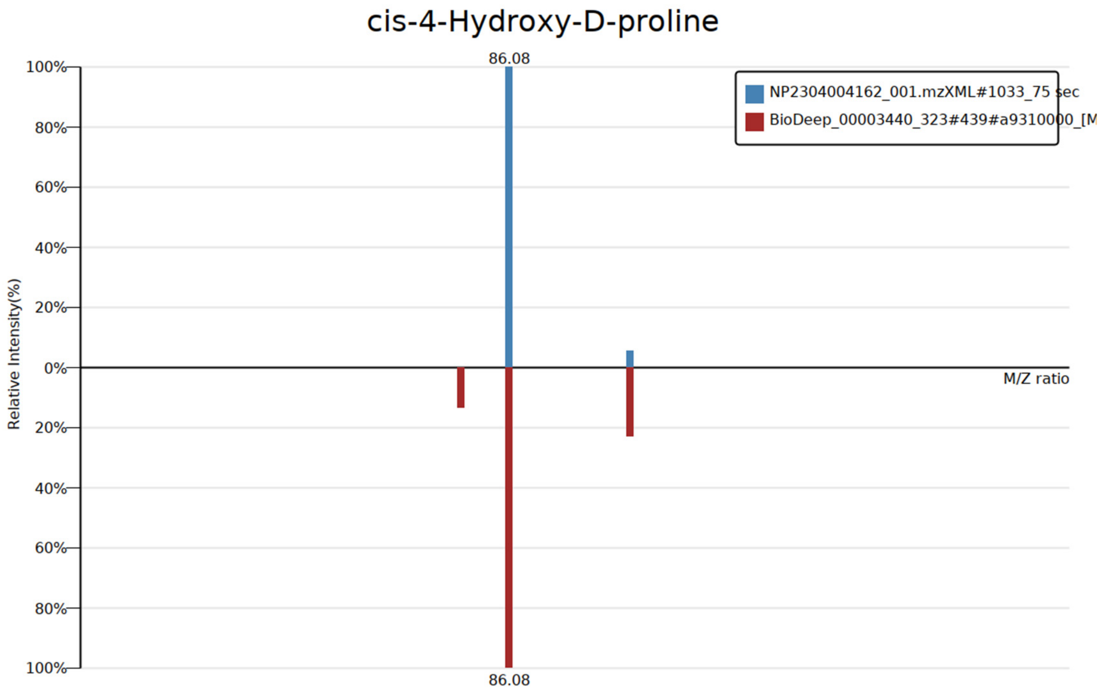

L-Asparagine

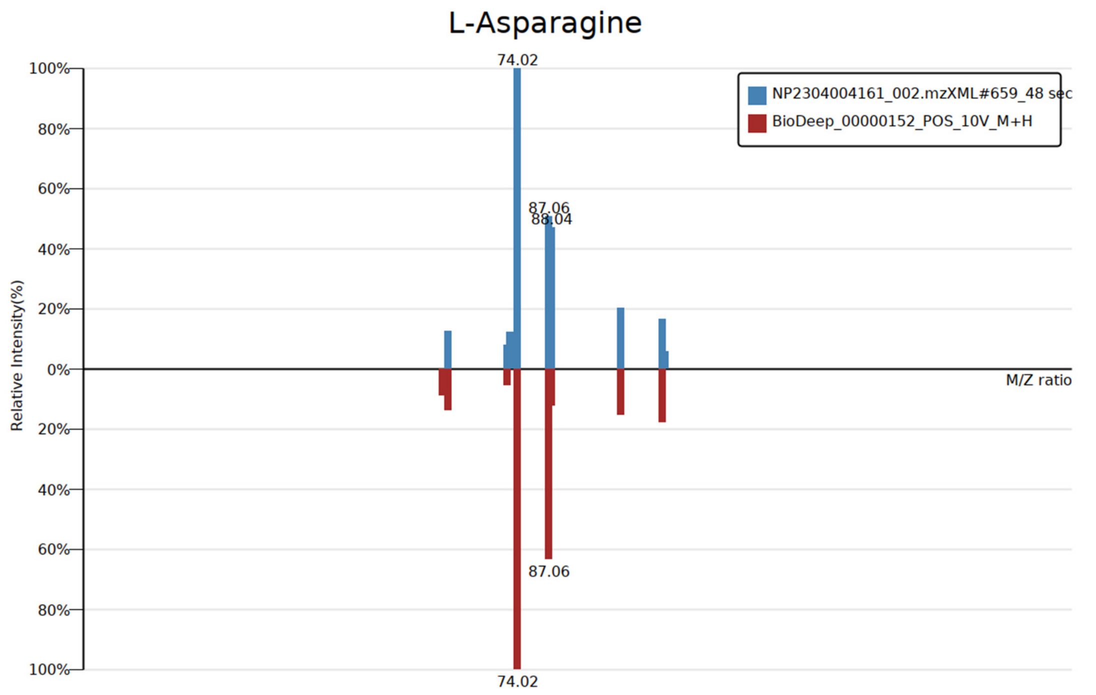

(2R,5S)-2,5-Diaminohexanoate

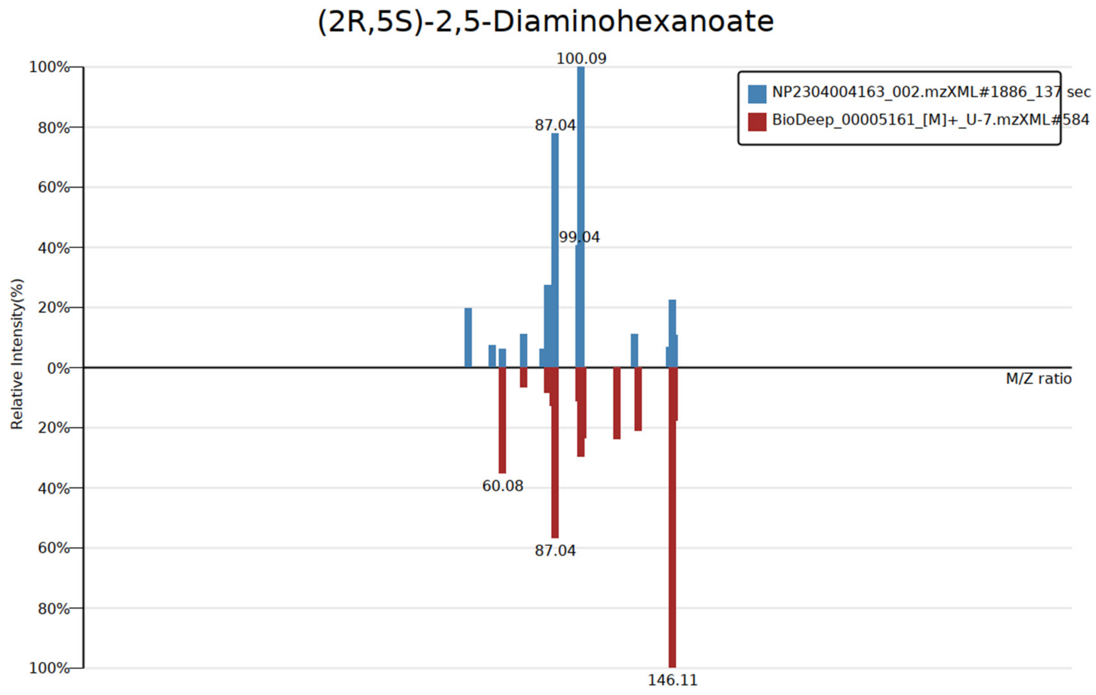

4-Guanidinobutanoic acid

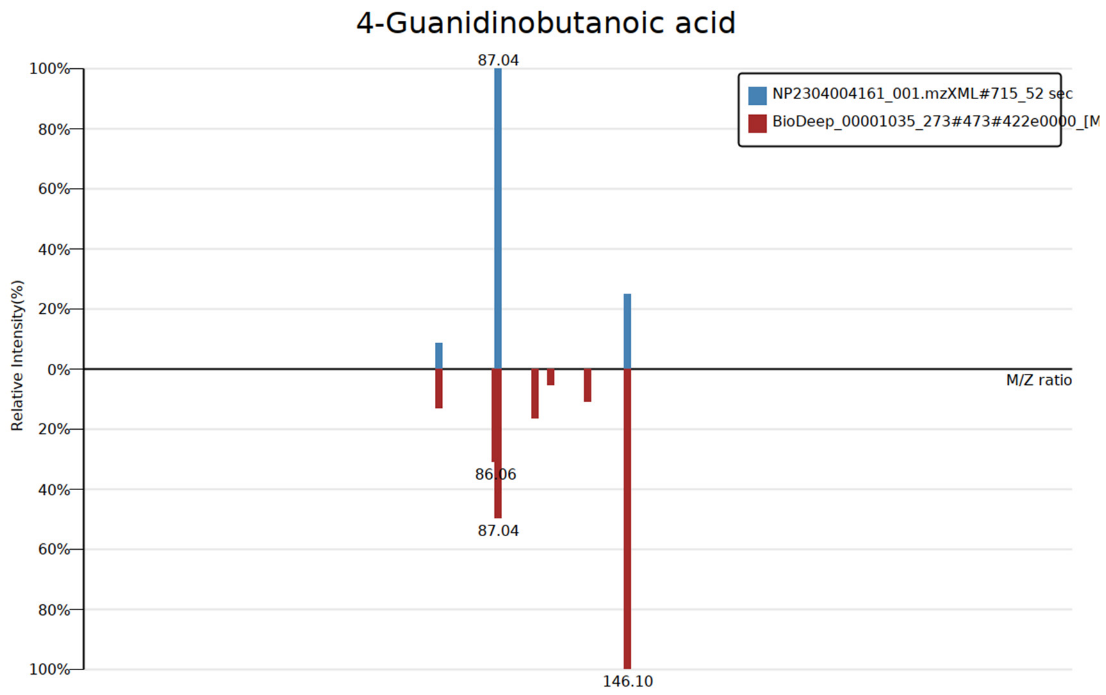

(3S,5S)-3,5-Diaminohexanoate

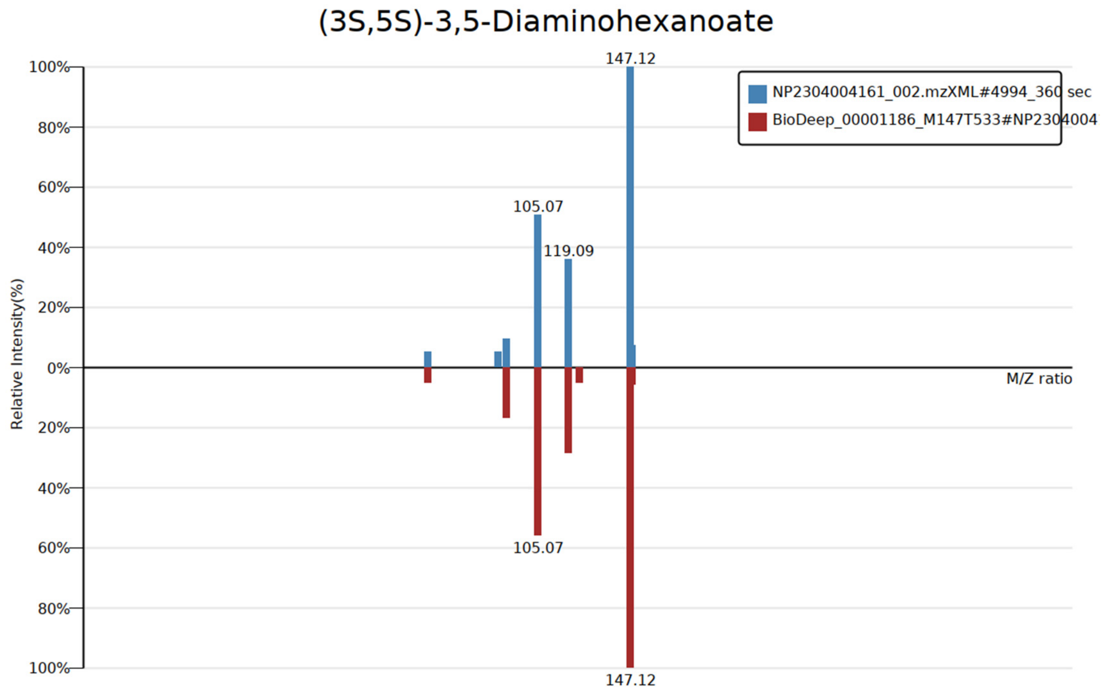

L-Glutamine

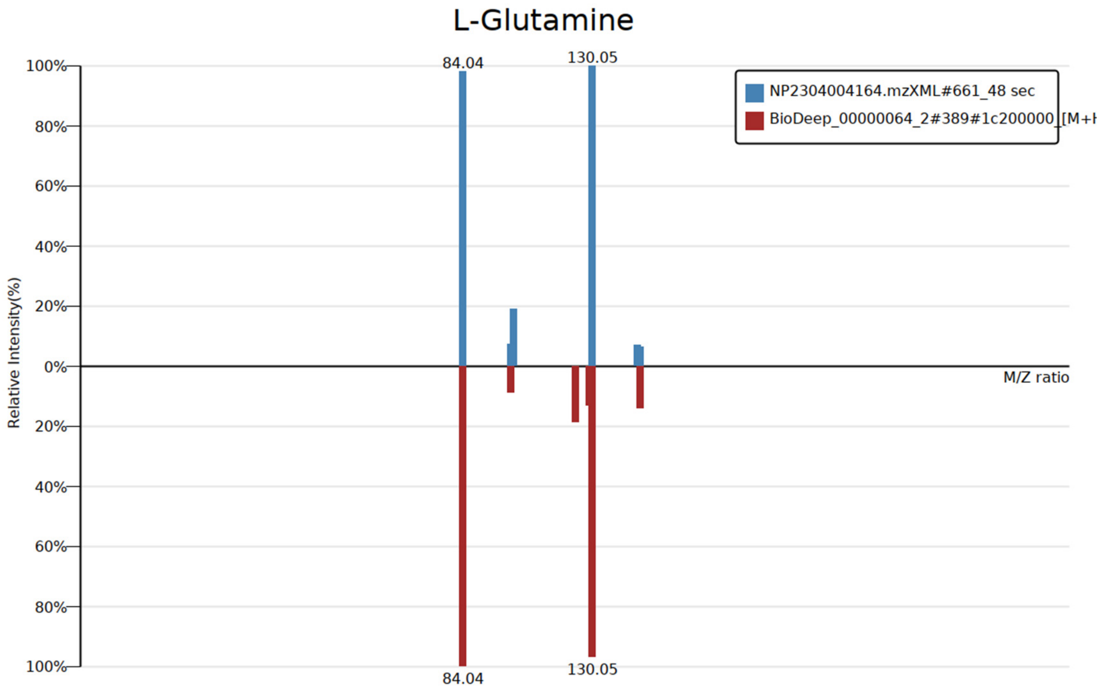

L-Glutamic acid

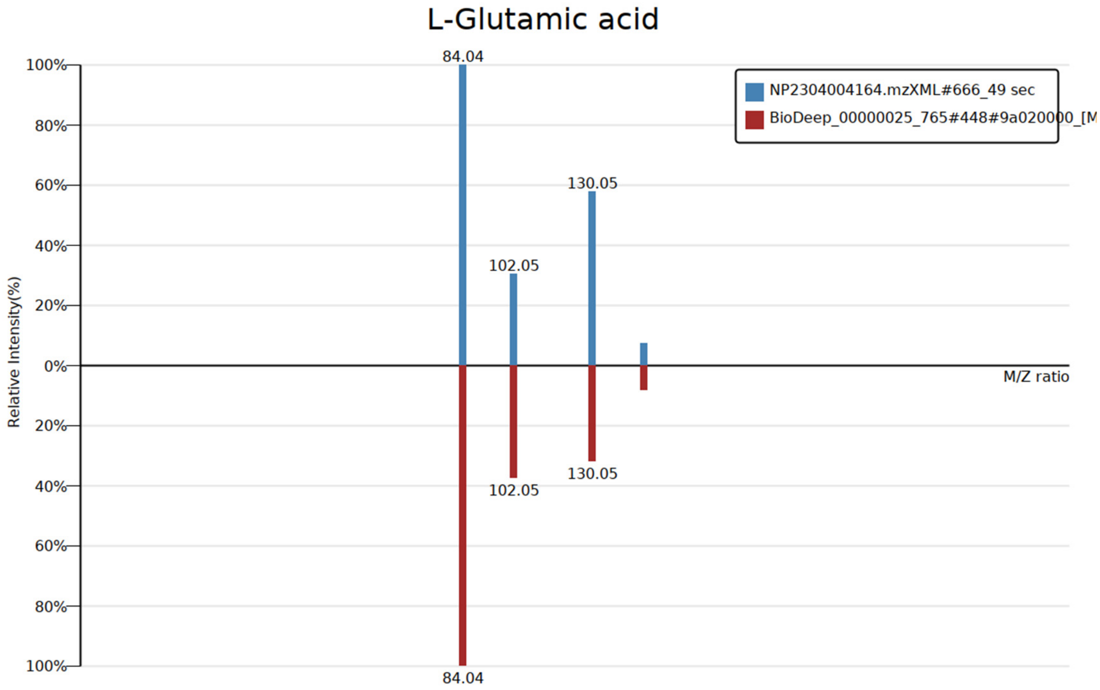

Acetylcysteine

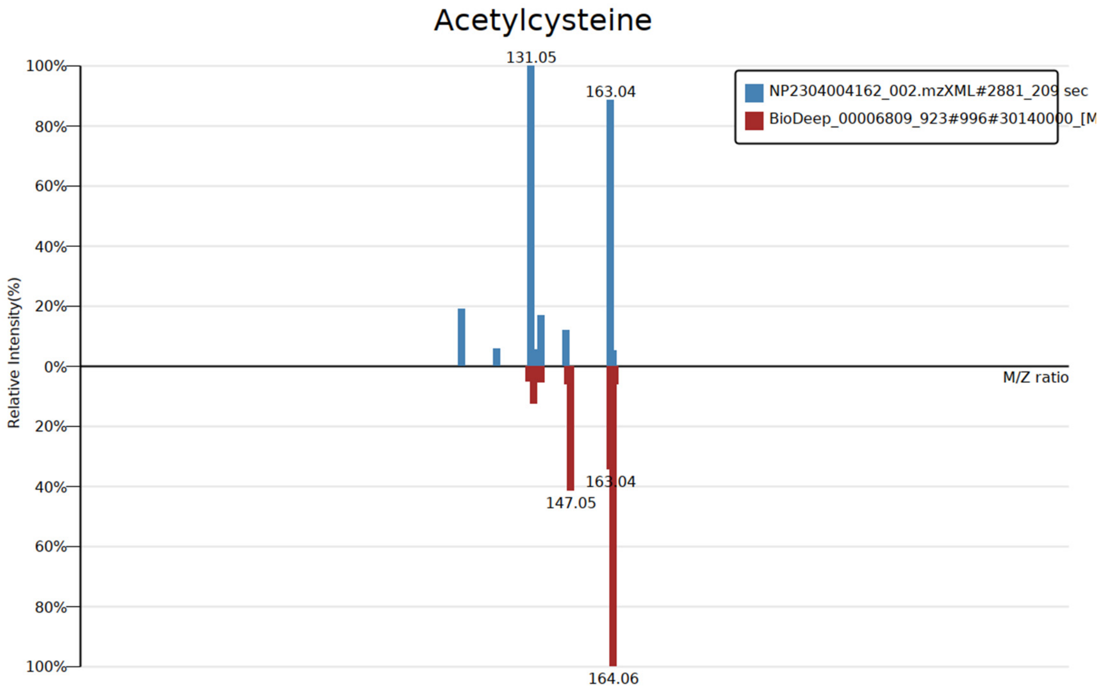

N(6)-Methyllysine

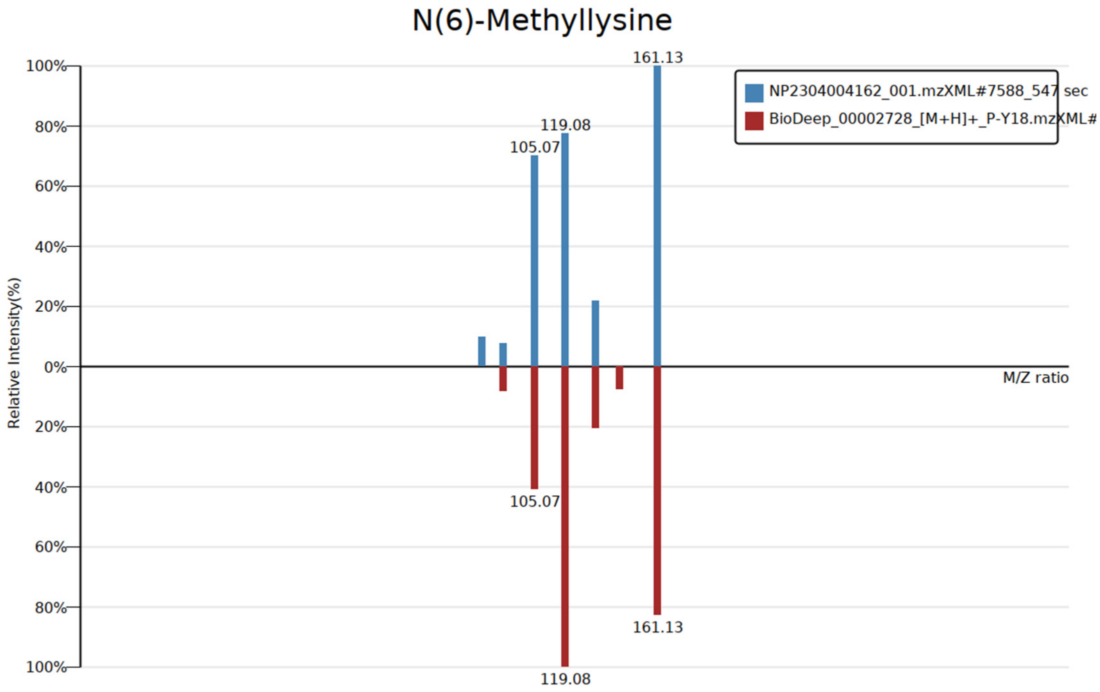

L-Arginine

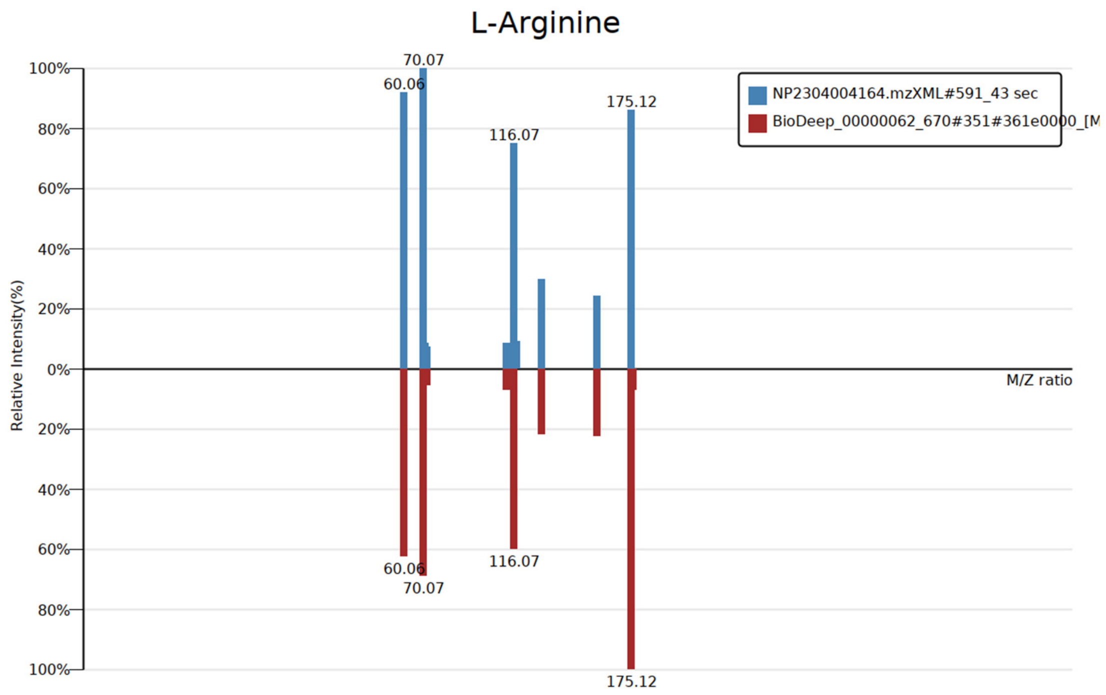

N-Acetylornithine

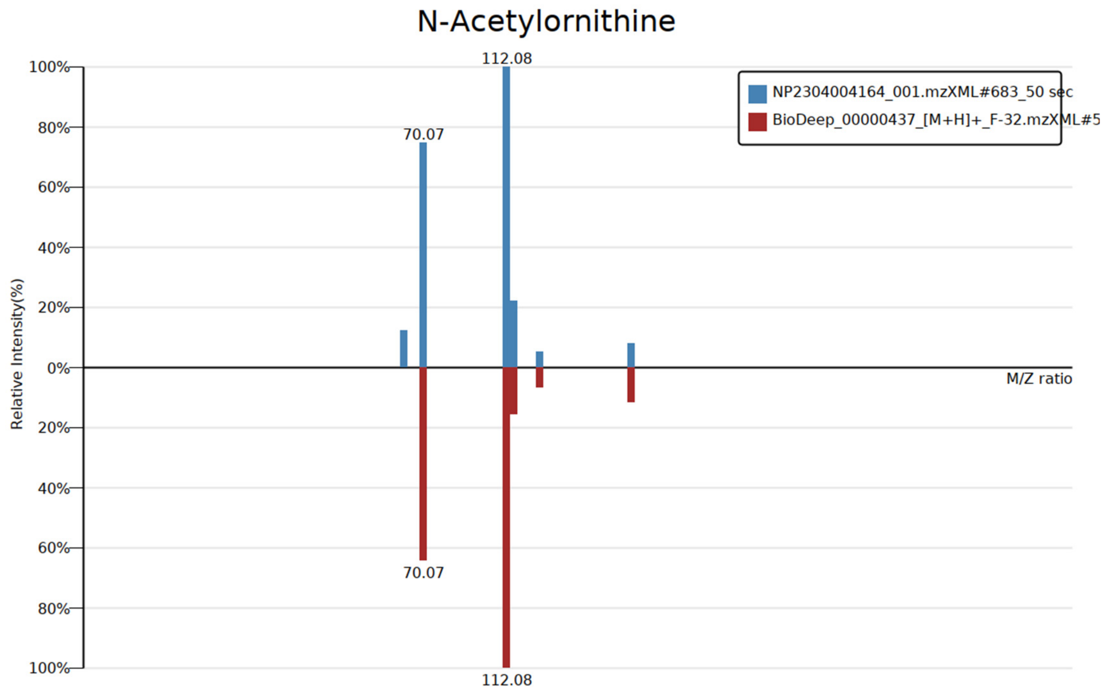

Beta-Tyrosine

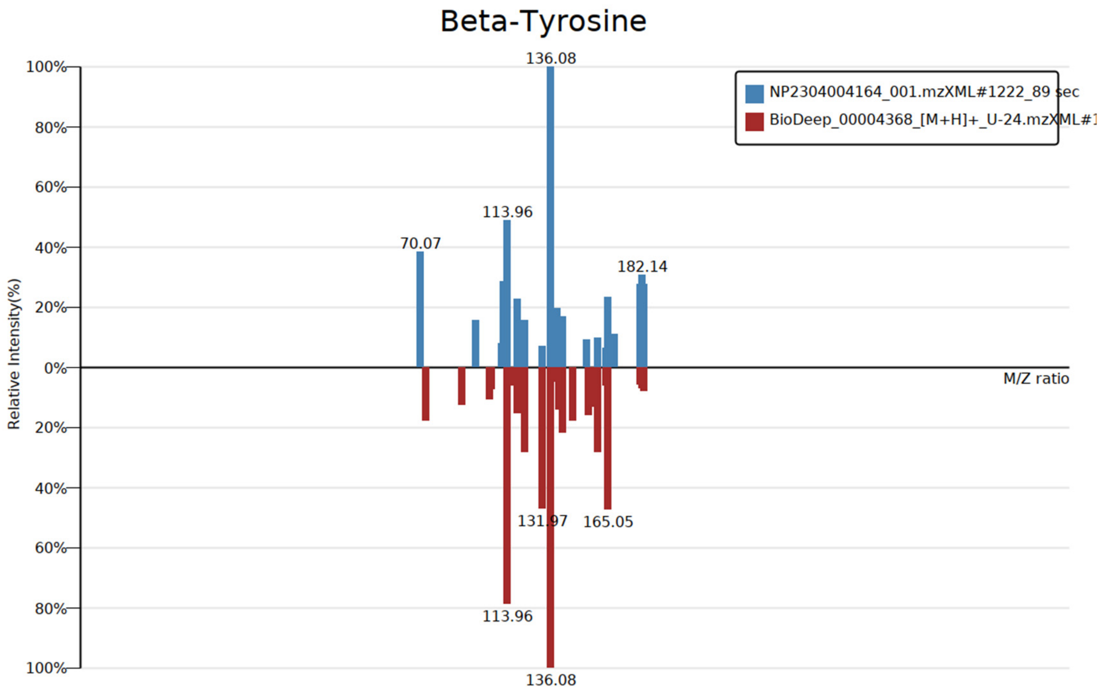

N-Alpha-acetyllysine

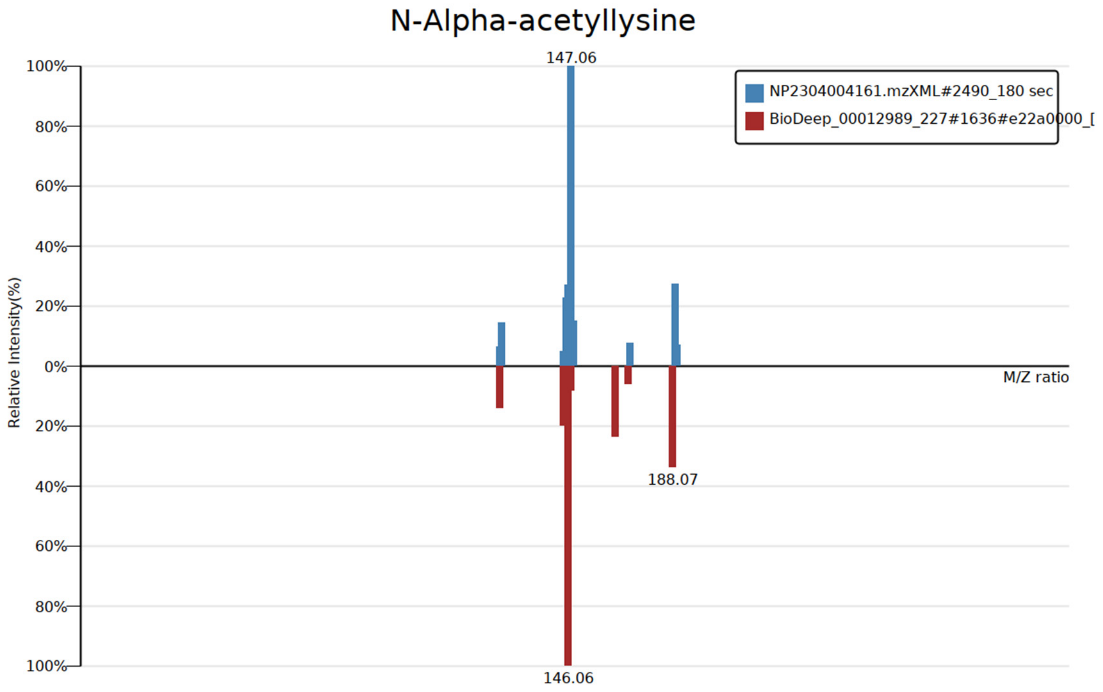

N-Acetylglutamic acid

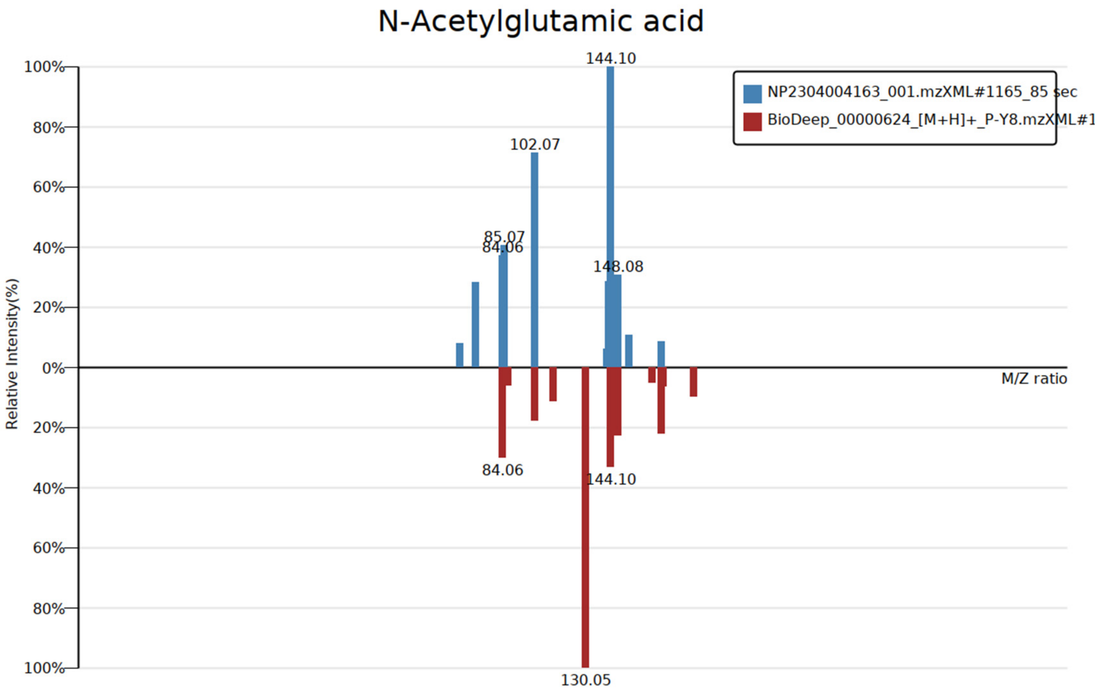

L-Dopa

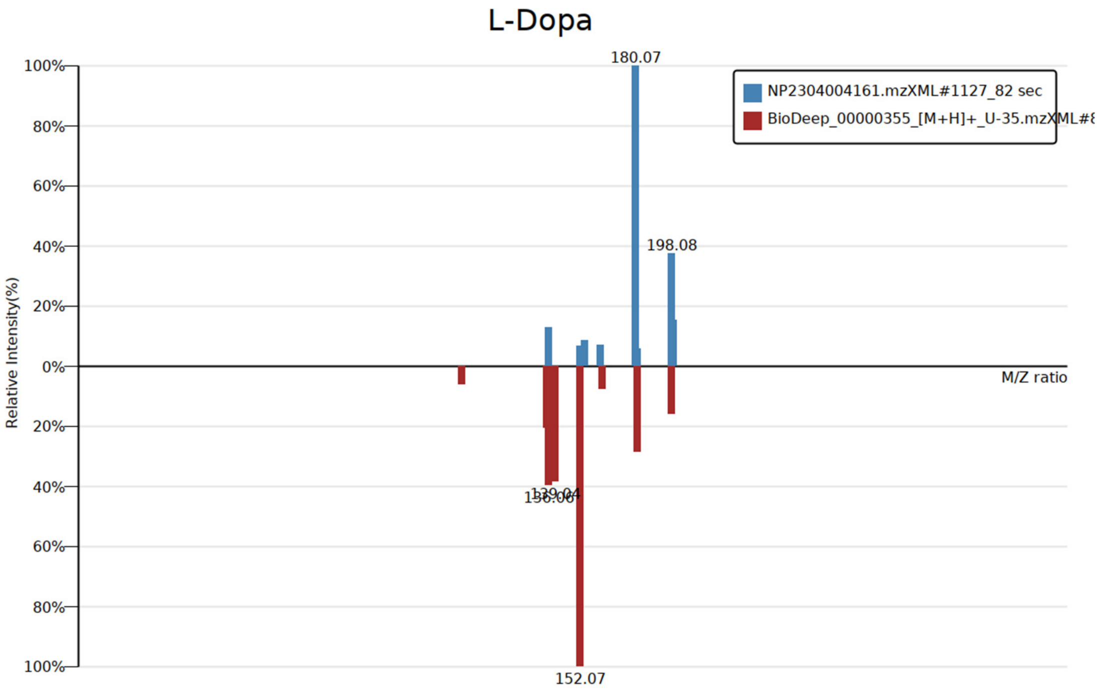

Saccharopine

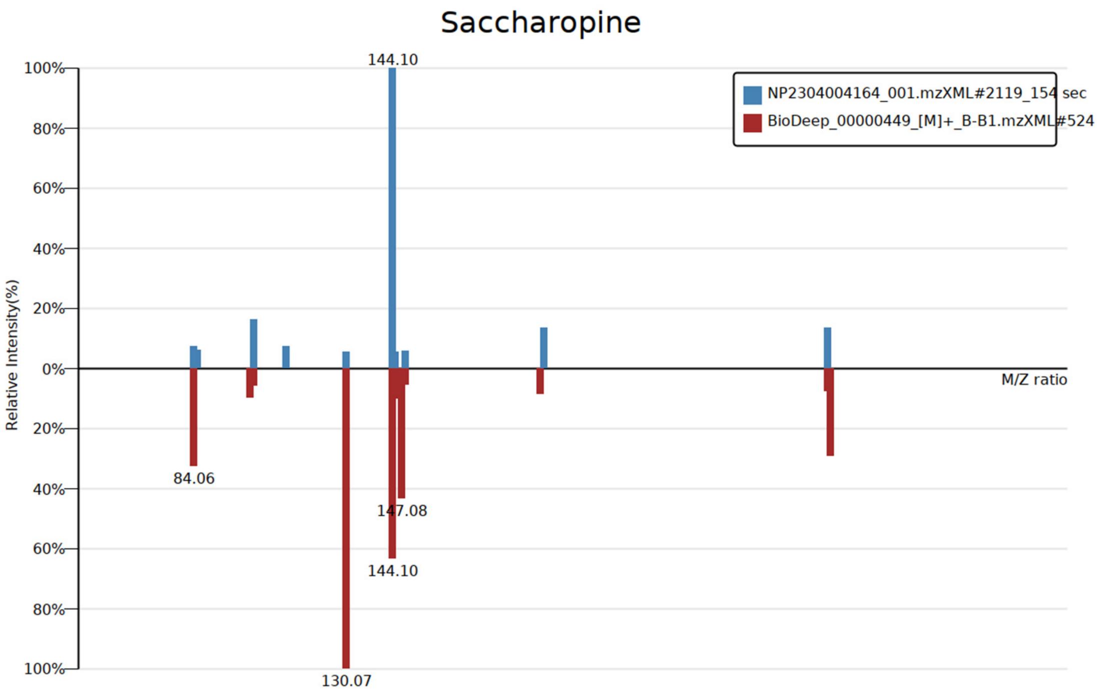

L-Norvaline

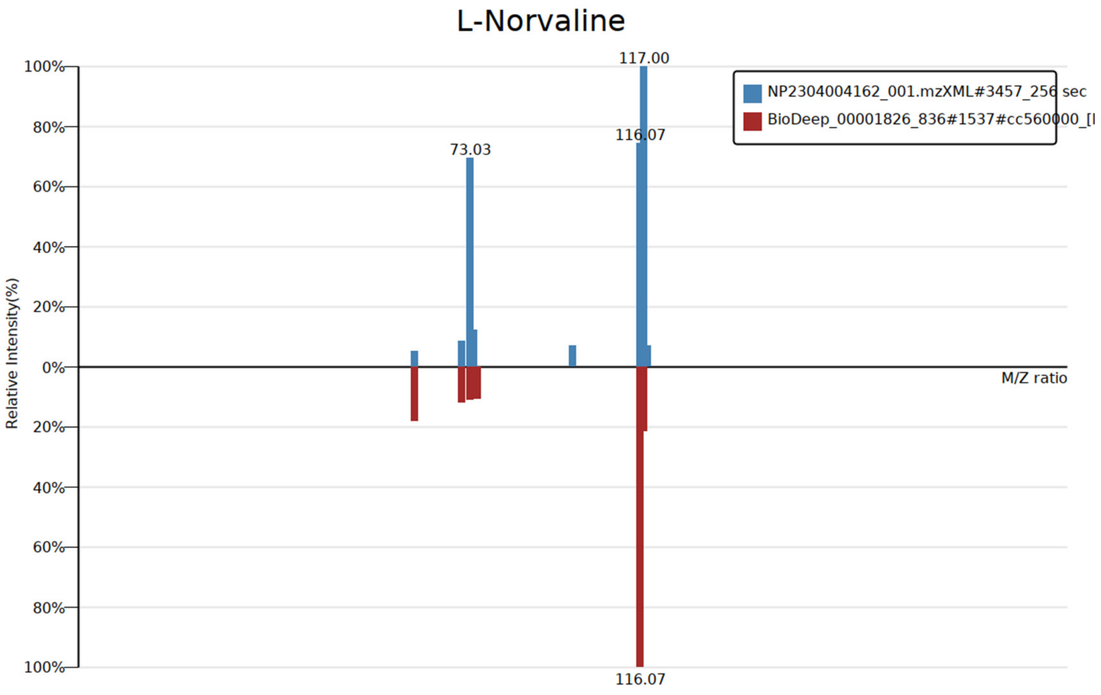

L-Phenylalanine

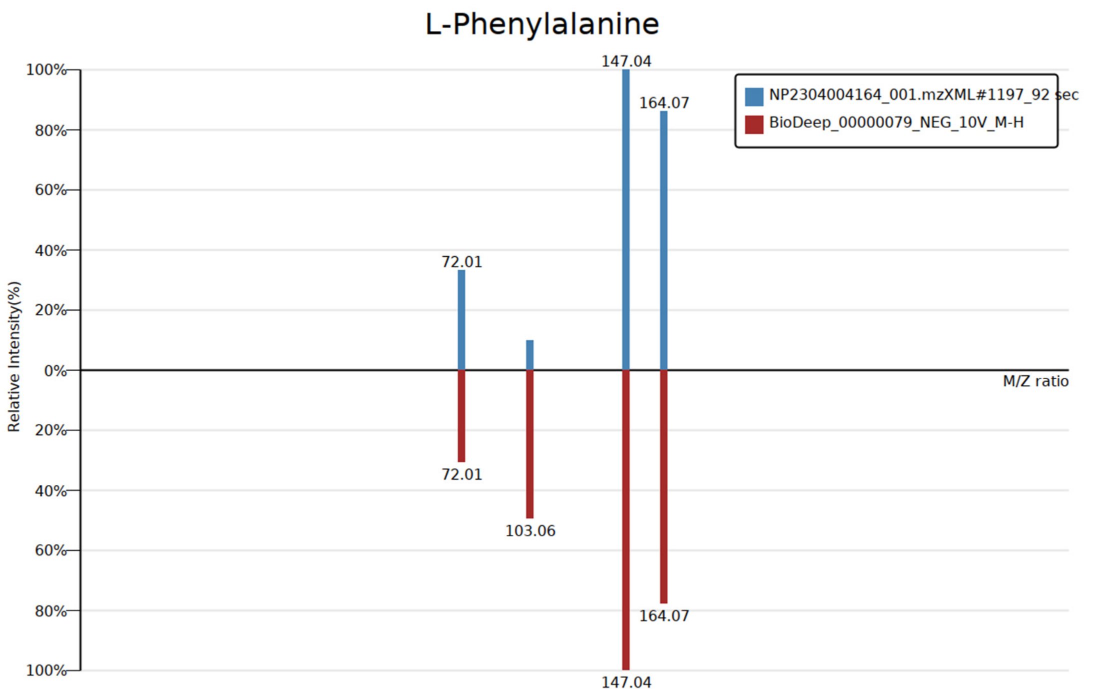

Desaminotyrosine

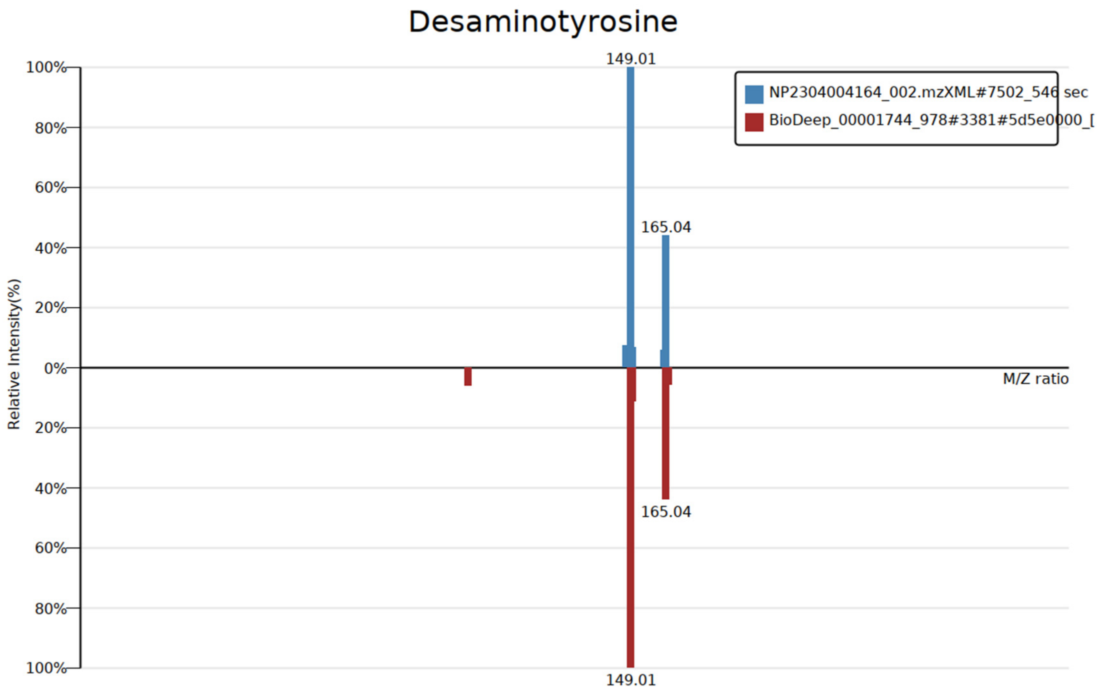

L-Tyrosine

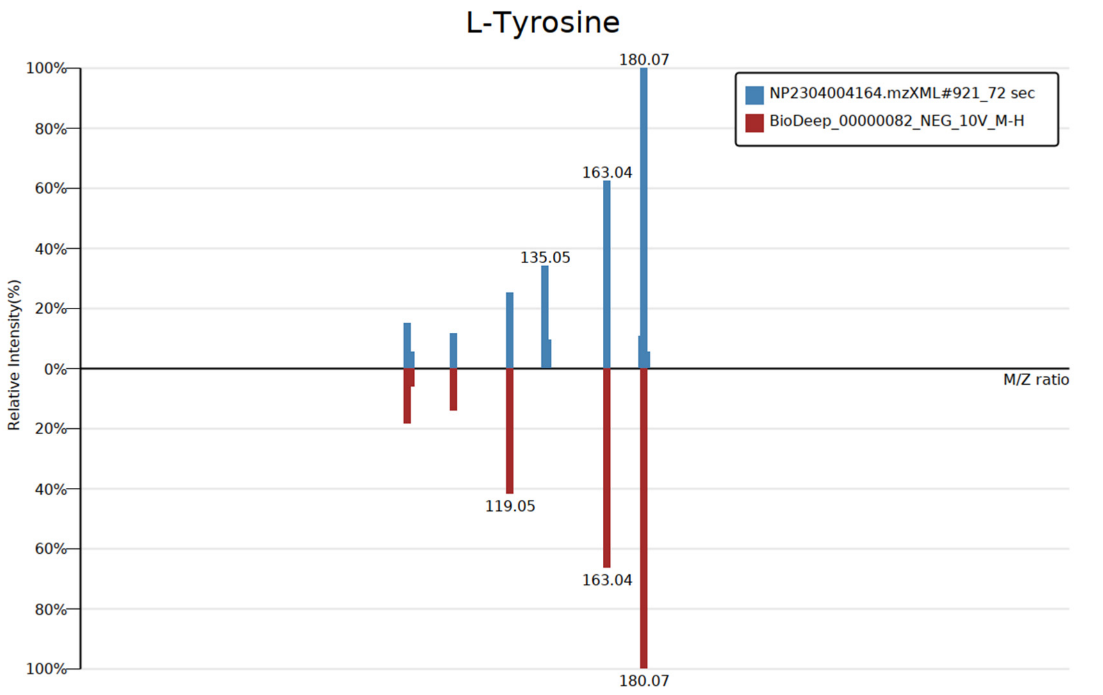

Tetrahydrodipicolinate

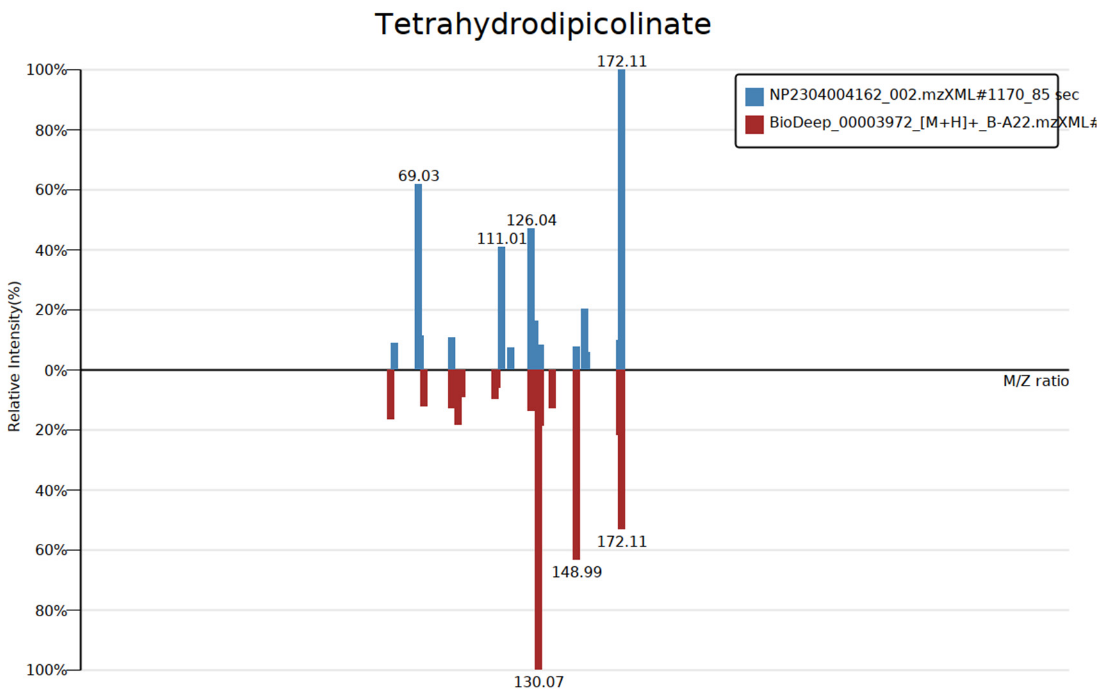

Imidazol-5-yl-pyruvate

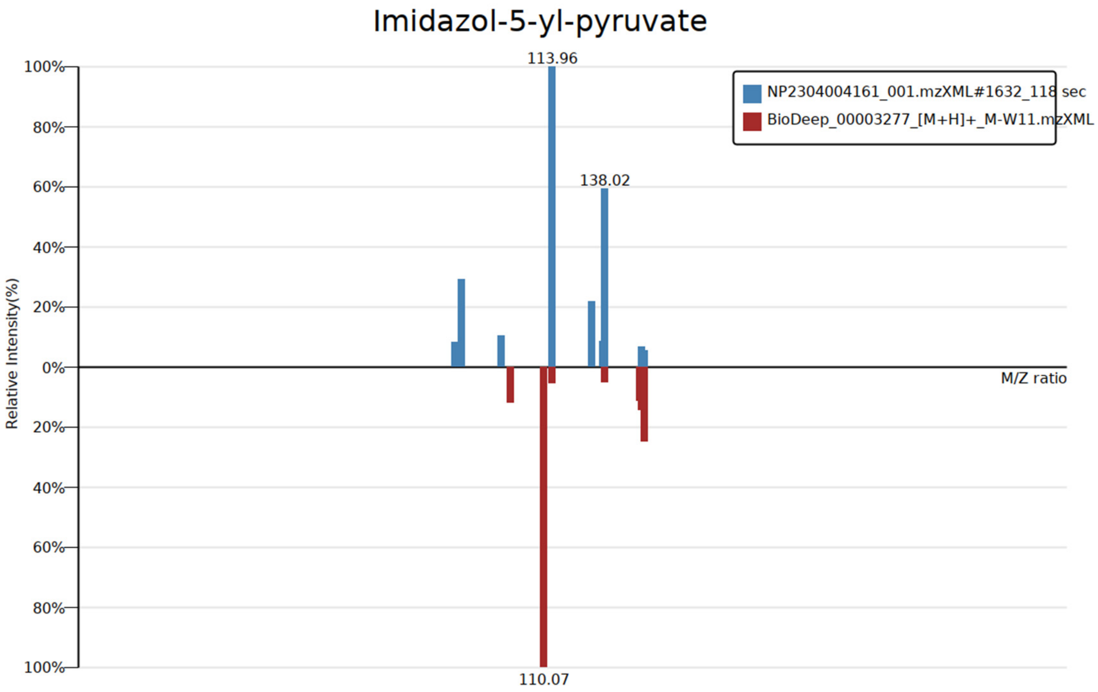

Glutarate semialdehyde

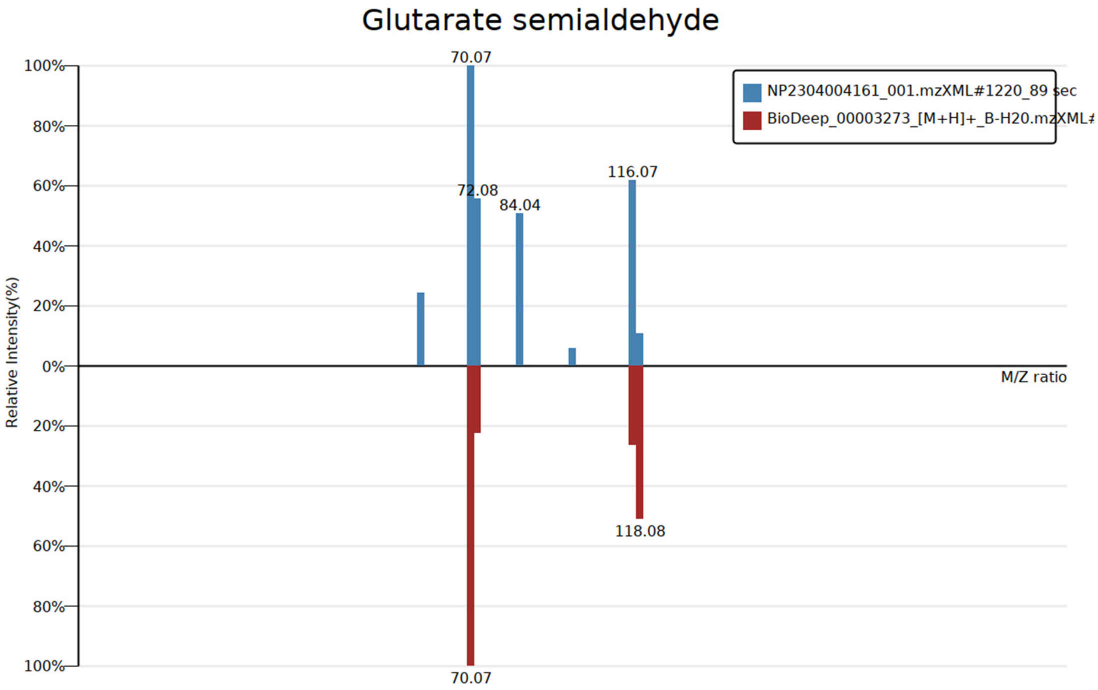

Phenylacetic acid

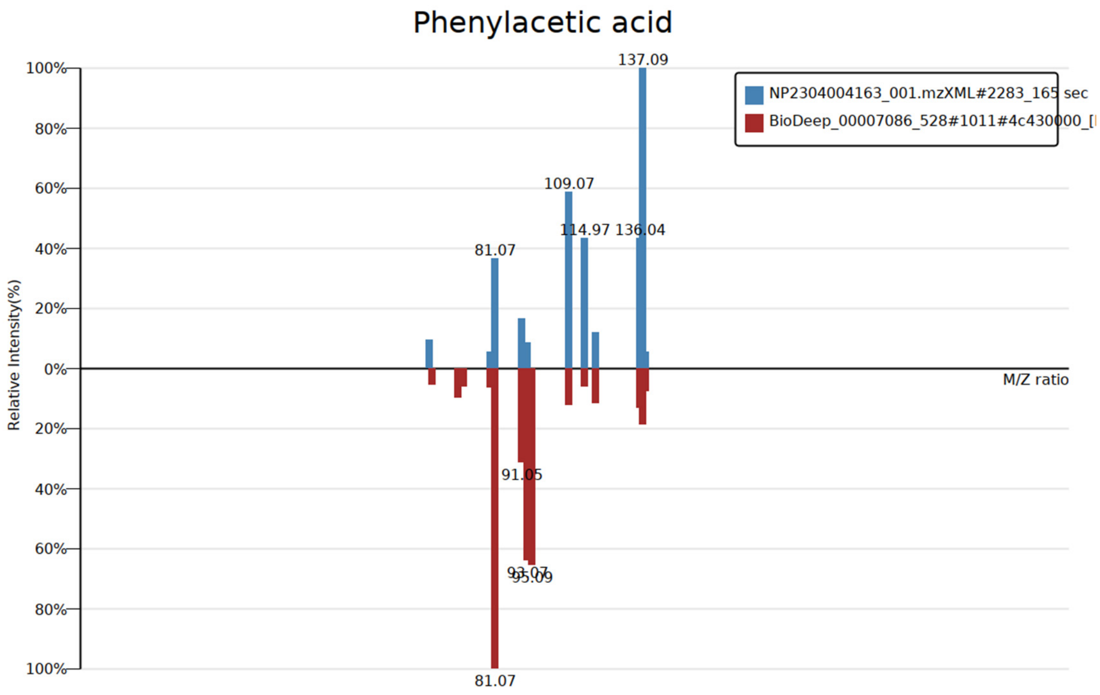

p-Aminobenzoic acid

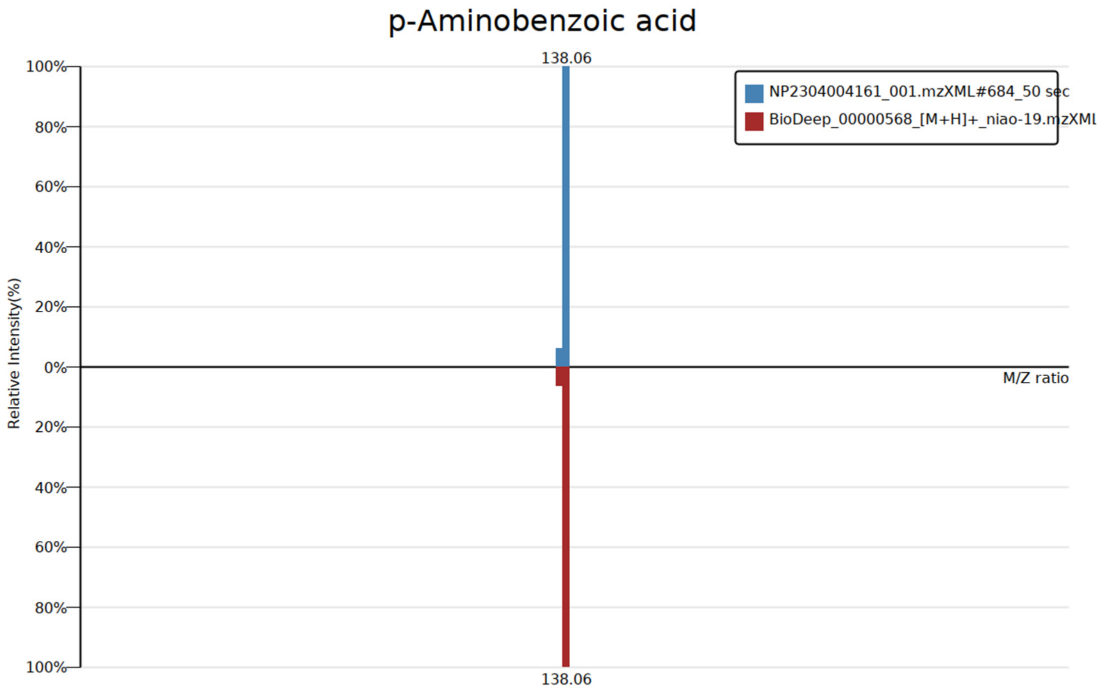

4,5-Dihydroorotic acid

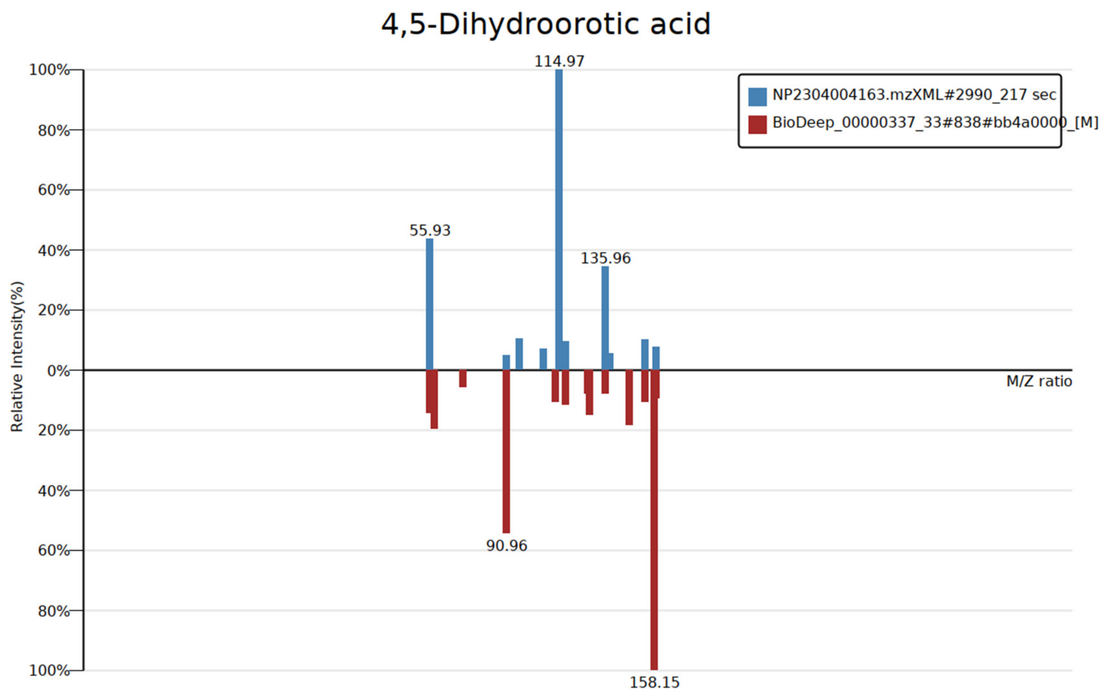

Tropate

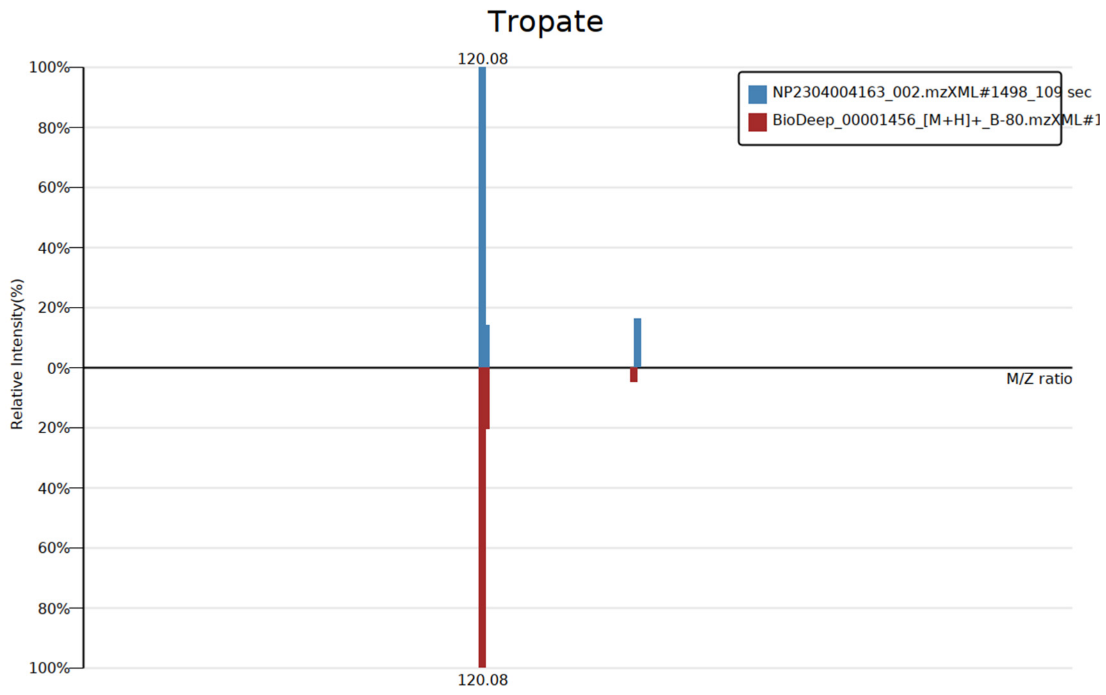

3-Succinoylpyridine

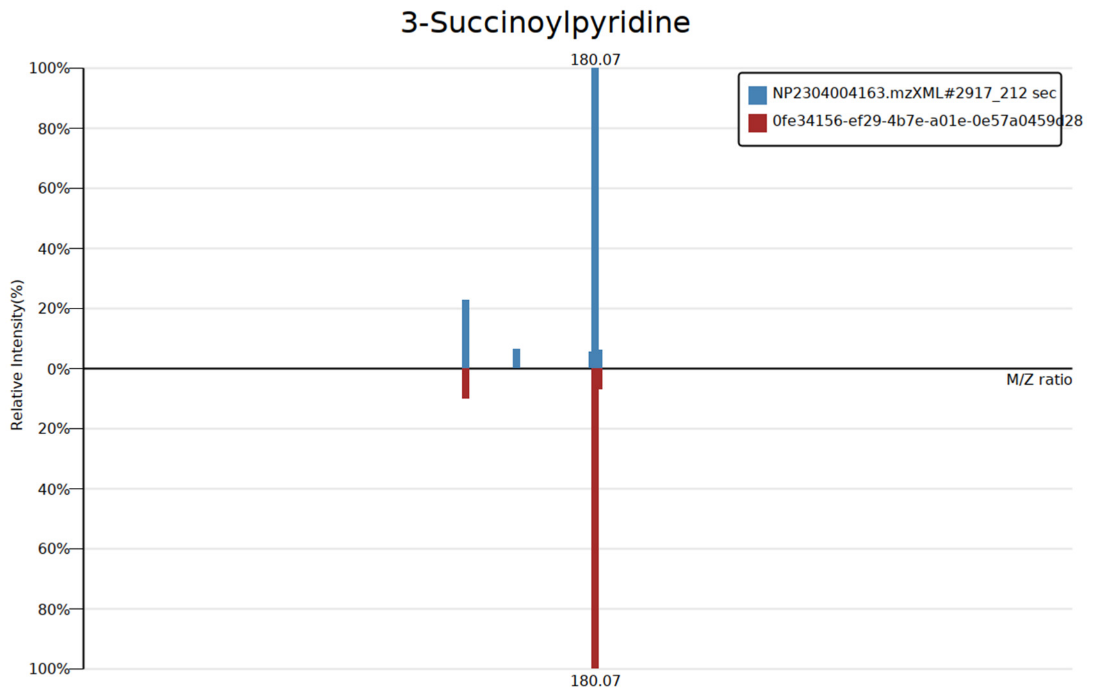

Kynurenic acid

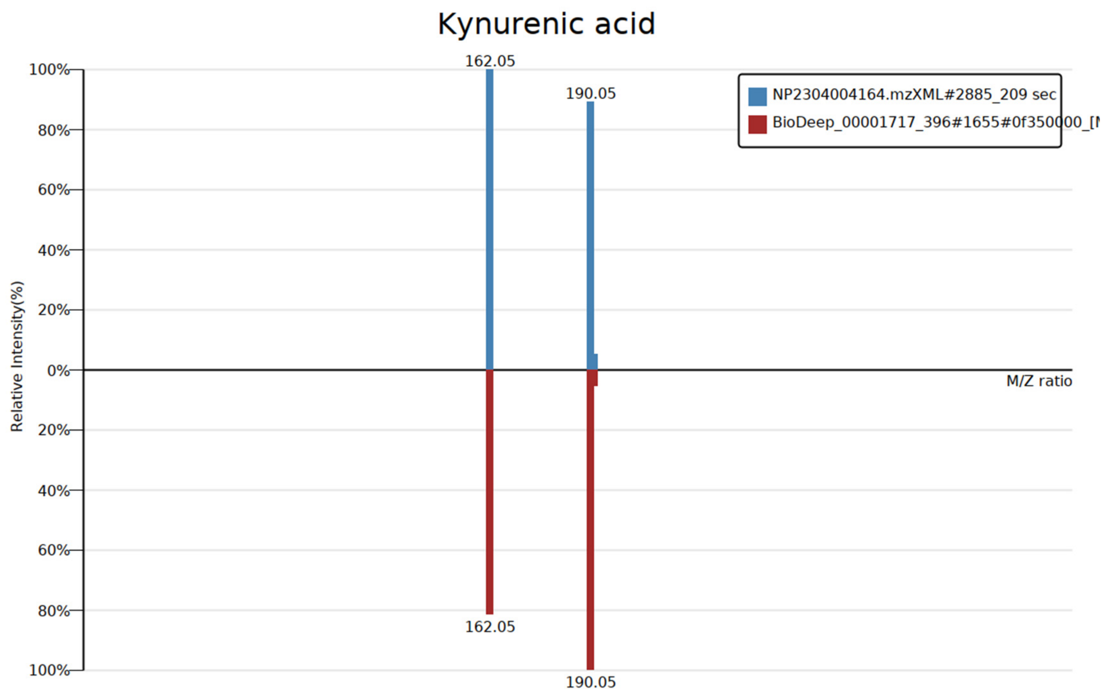

5-Methoxyindoleacetate

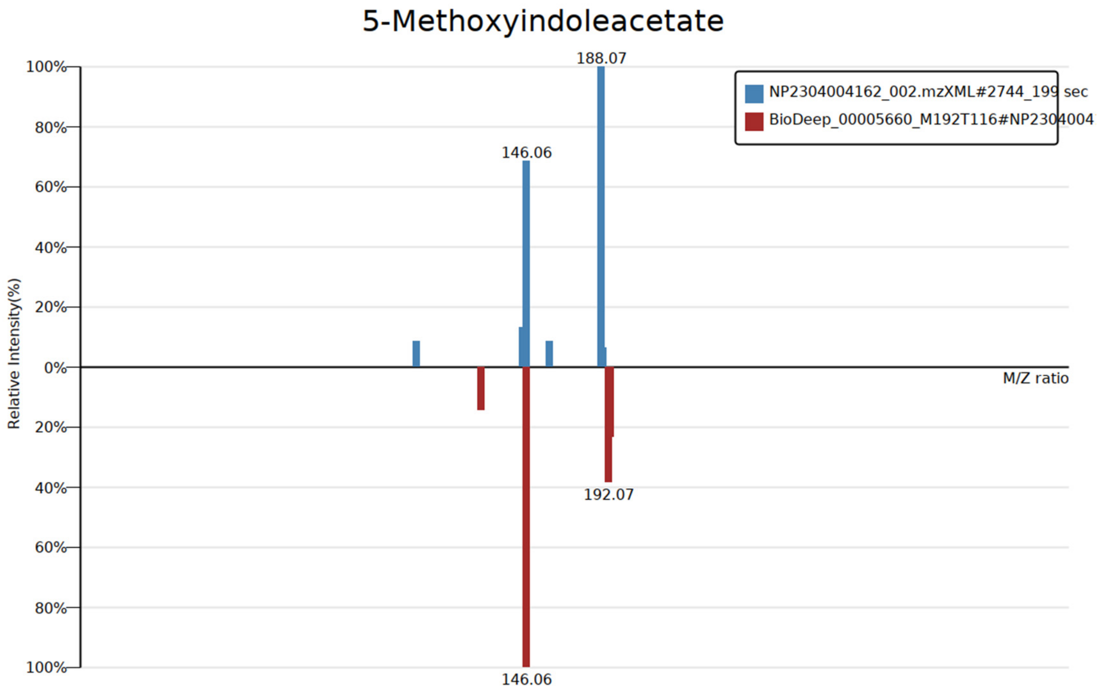

Pantothenic acid

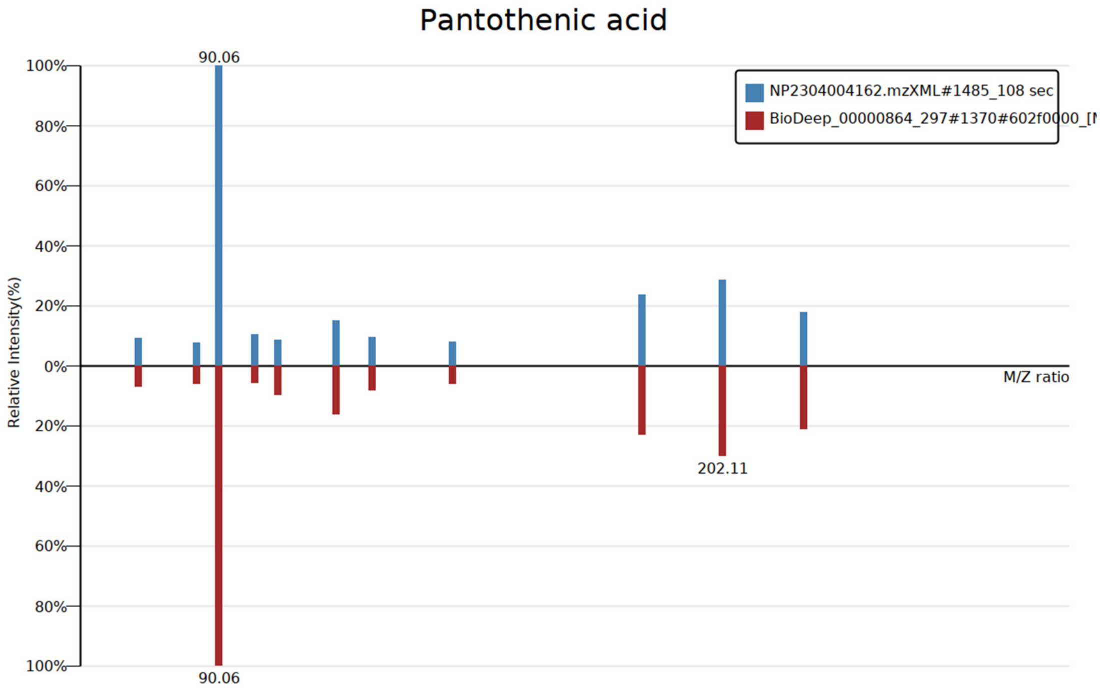

9,10-EOT

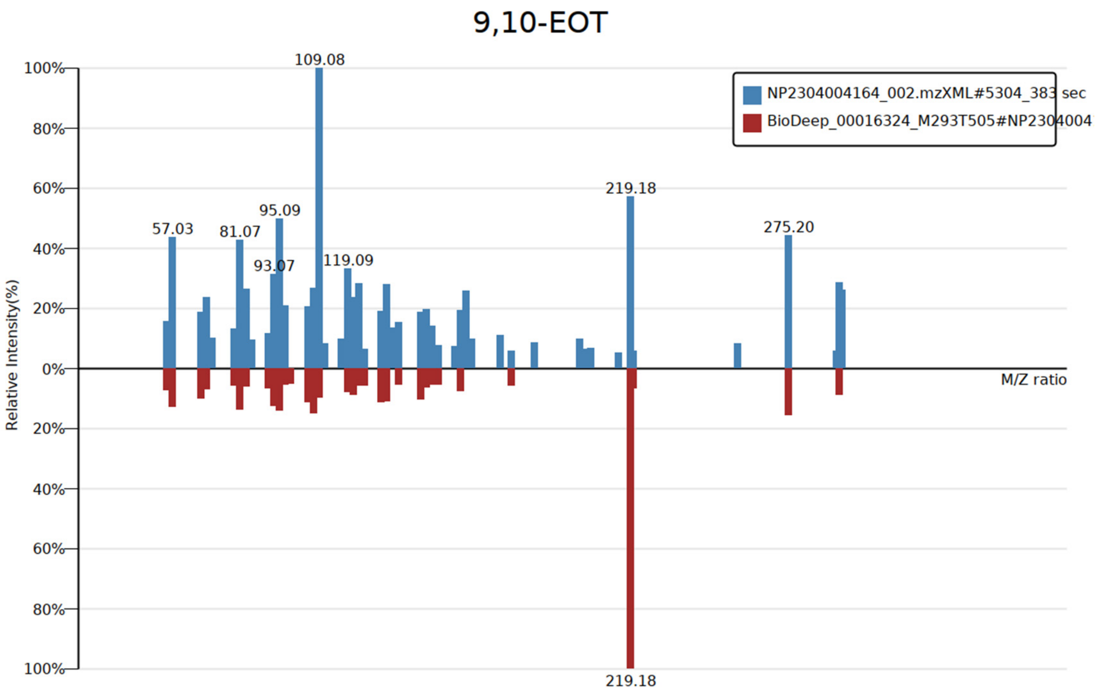

9(S)-HPODE

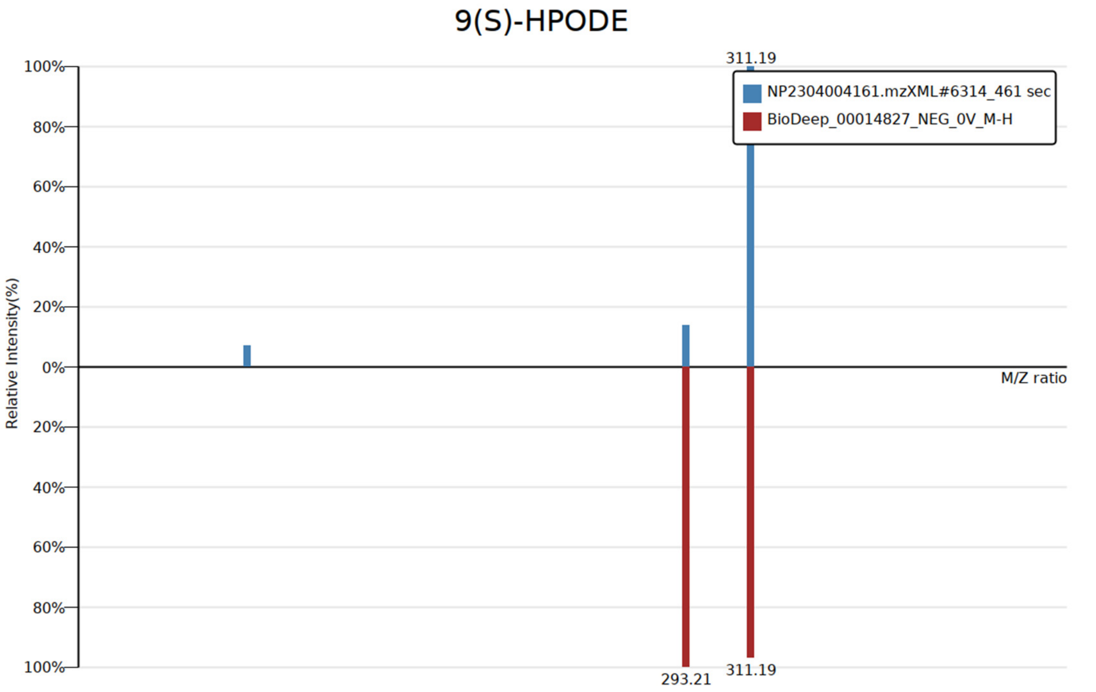

13-L-Hydroperoxylinoleic acid

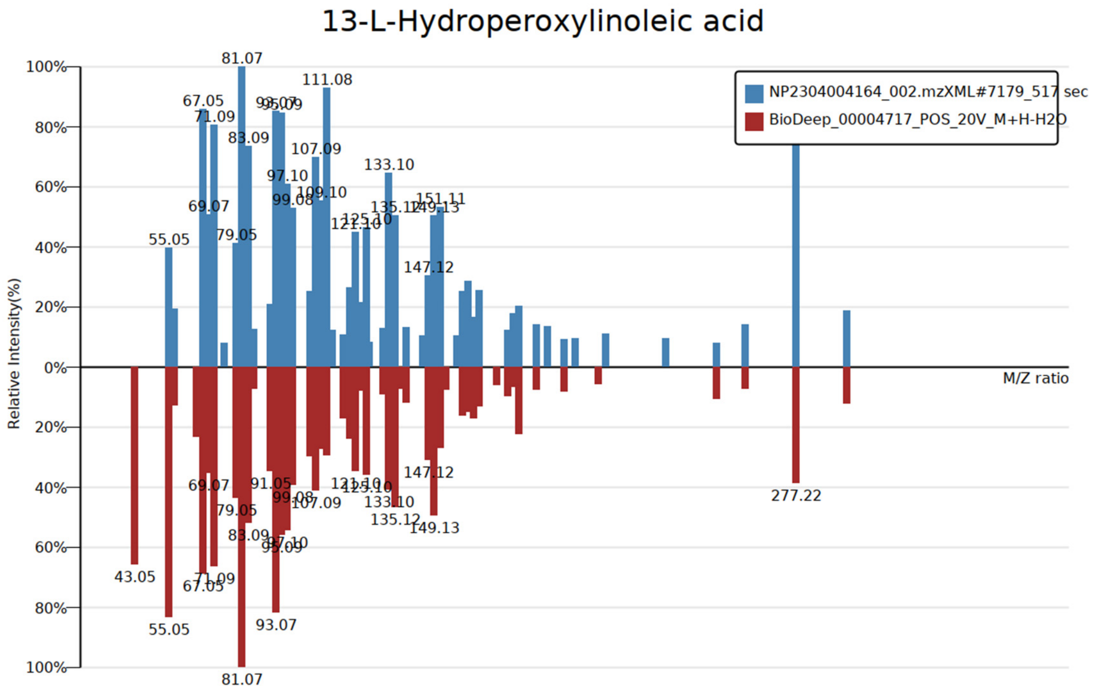

5-Nitro-2-(3-phenylpropylamino)benzoic acid

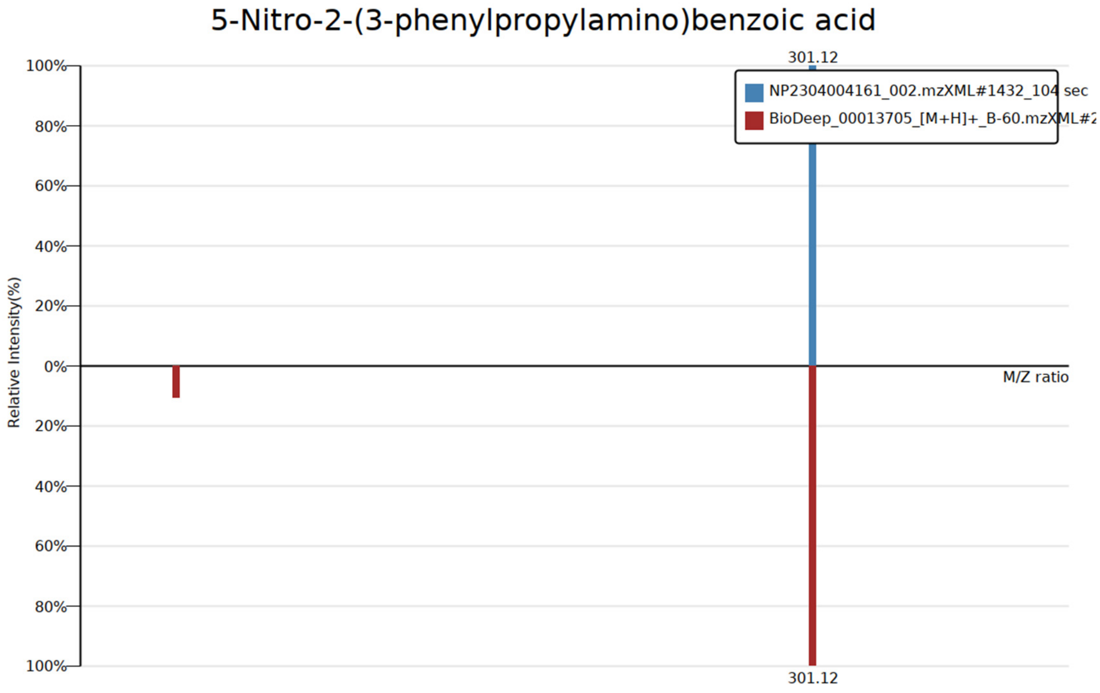

19(R)-HETE

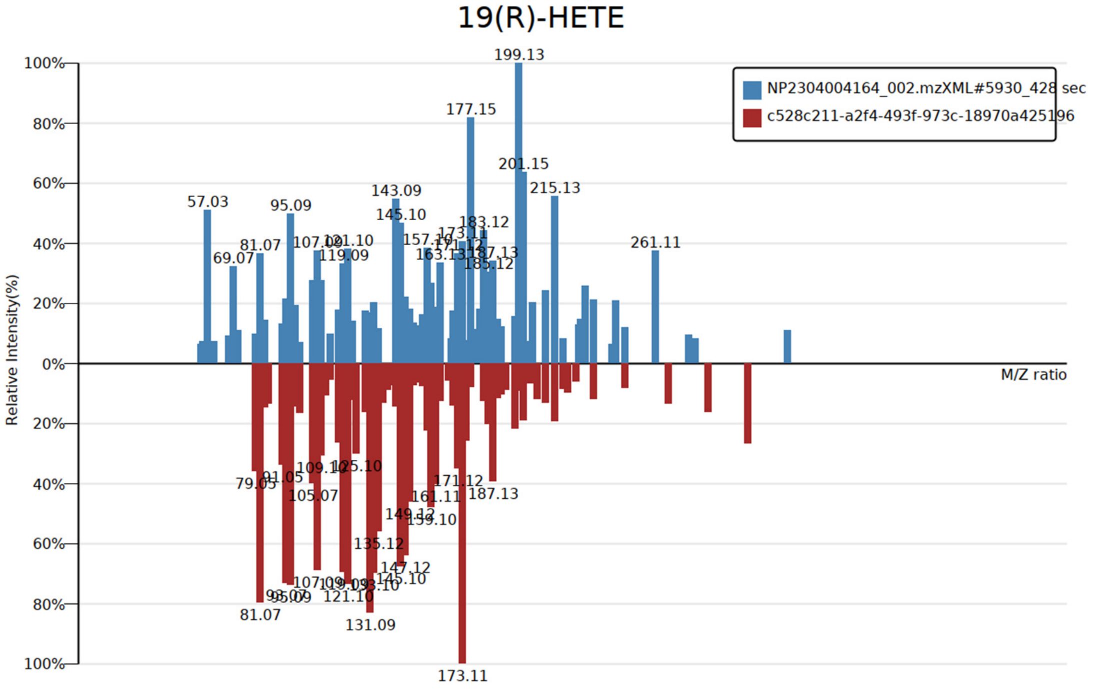

Malonate

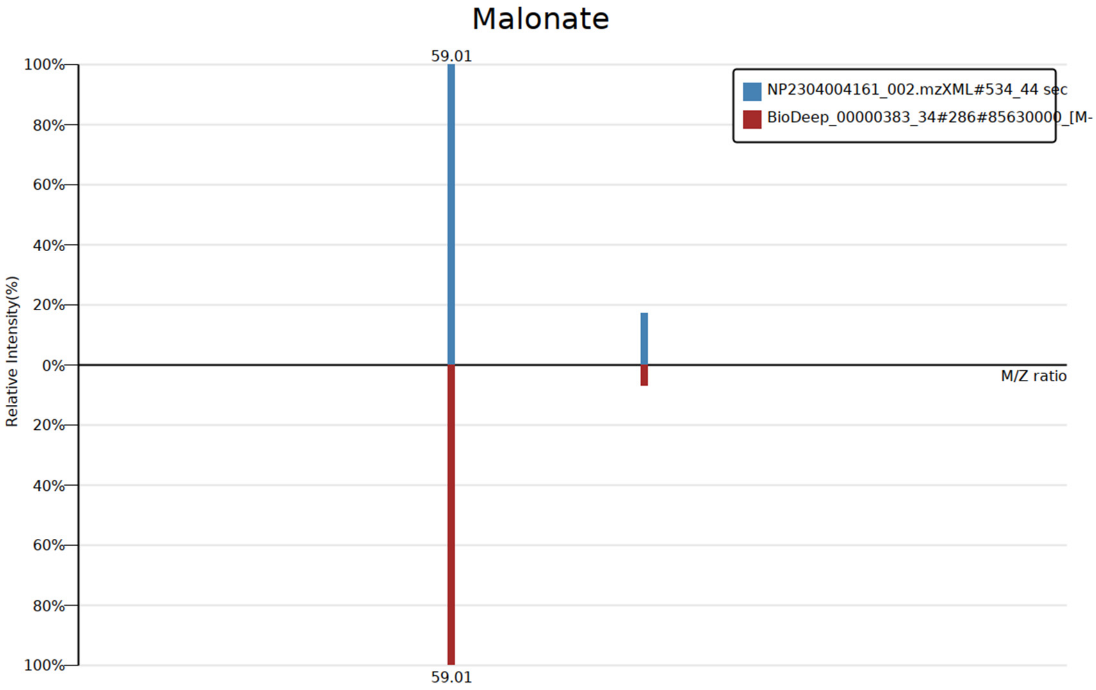

Glyceric acid

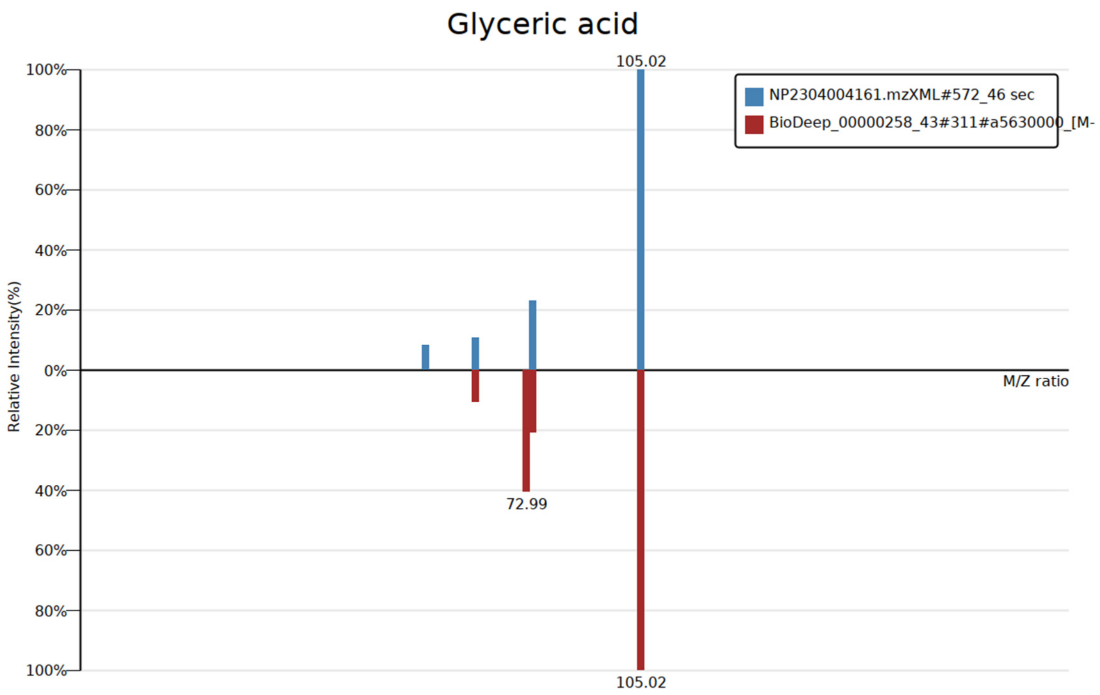

Succinic acid

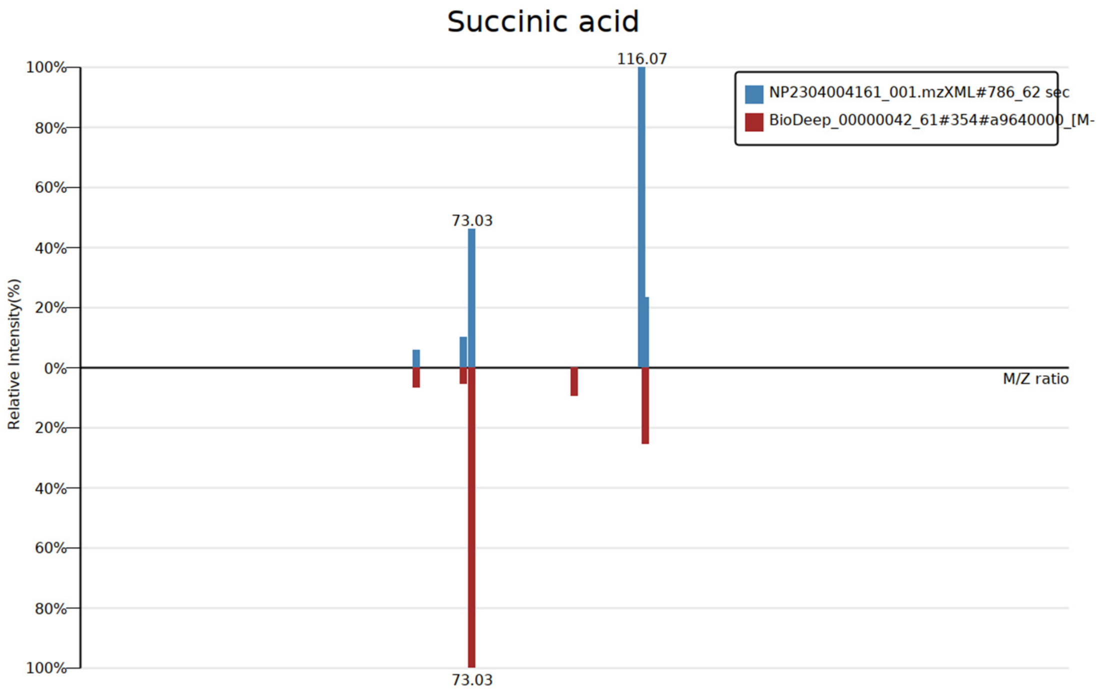

Nicotinic acid

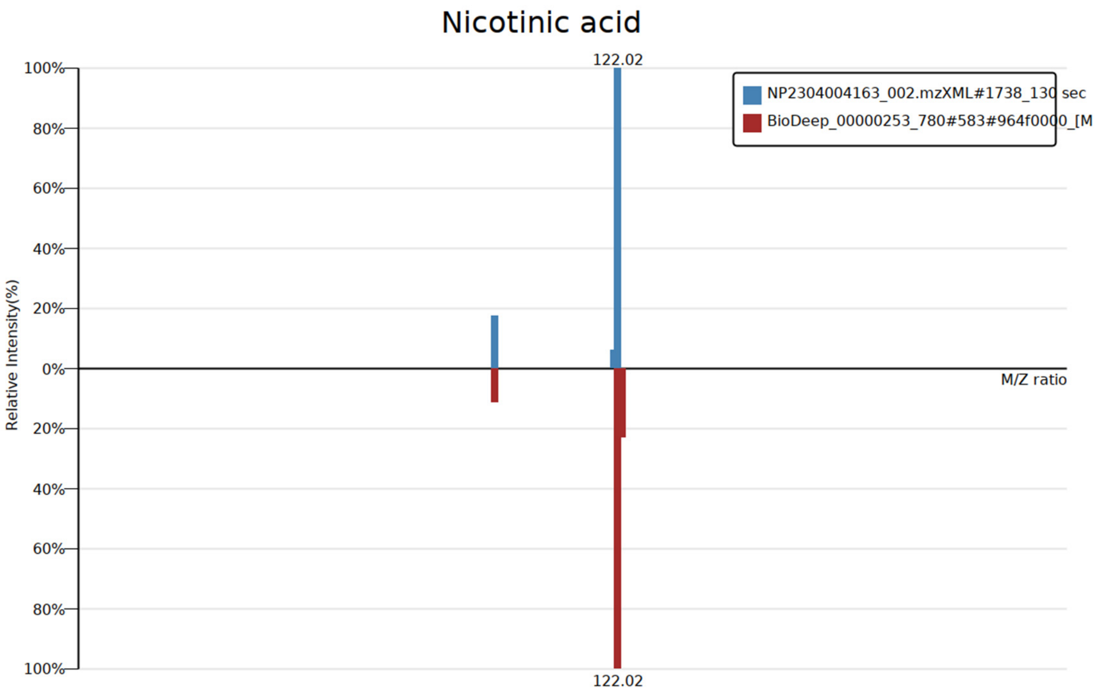

3-Methyl-2-oxovaleric acid

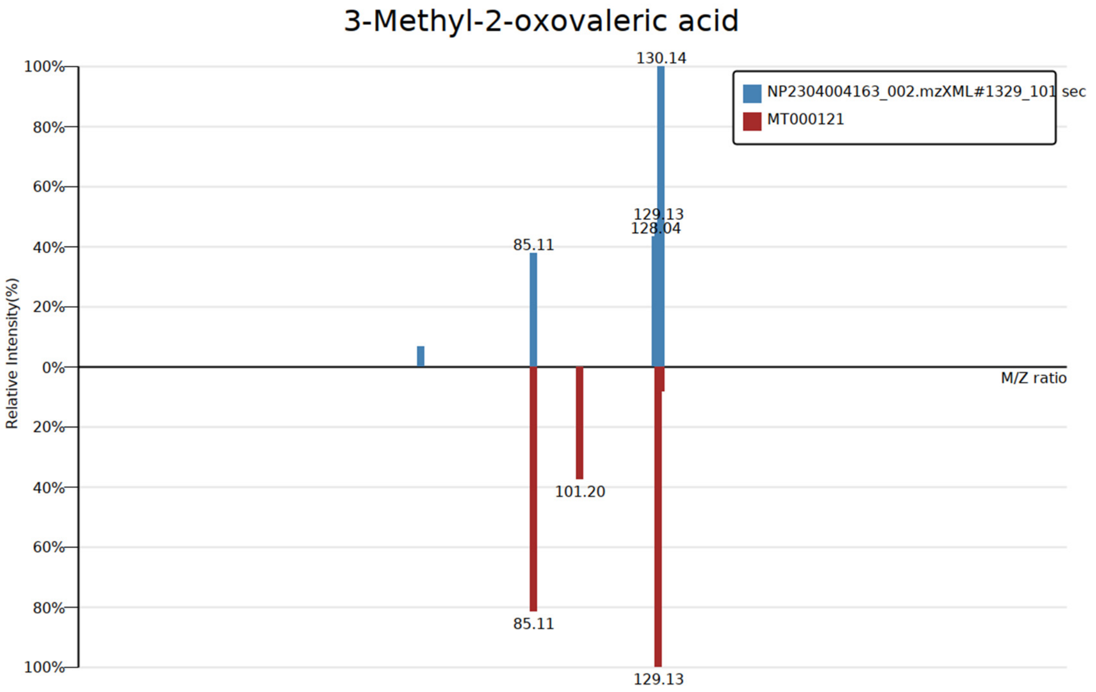

L-Malic acid

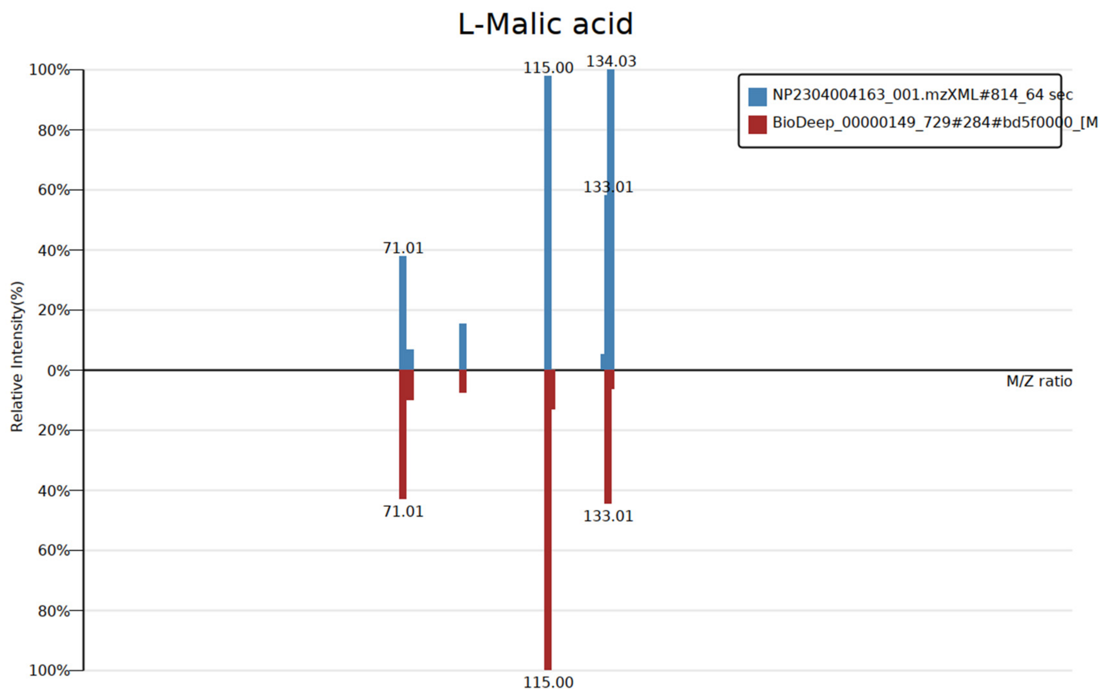

Phenyl acetate

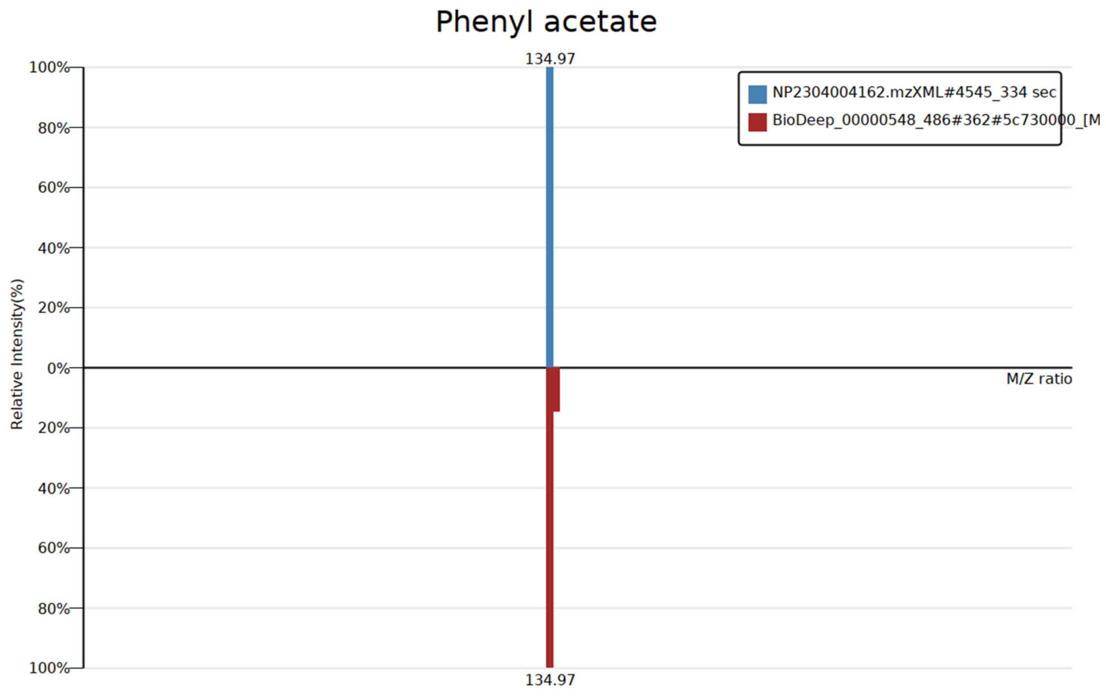

Salicylic acid

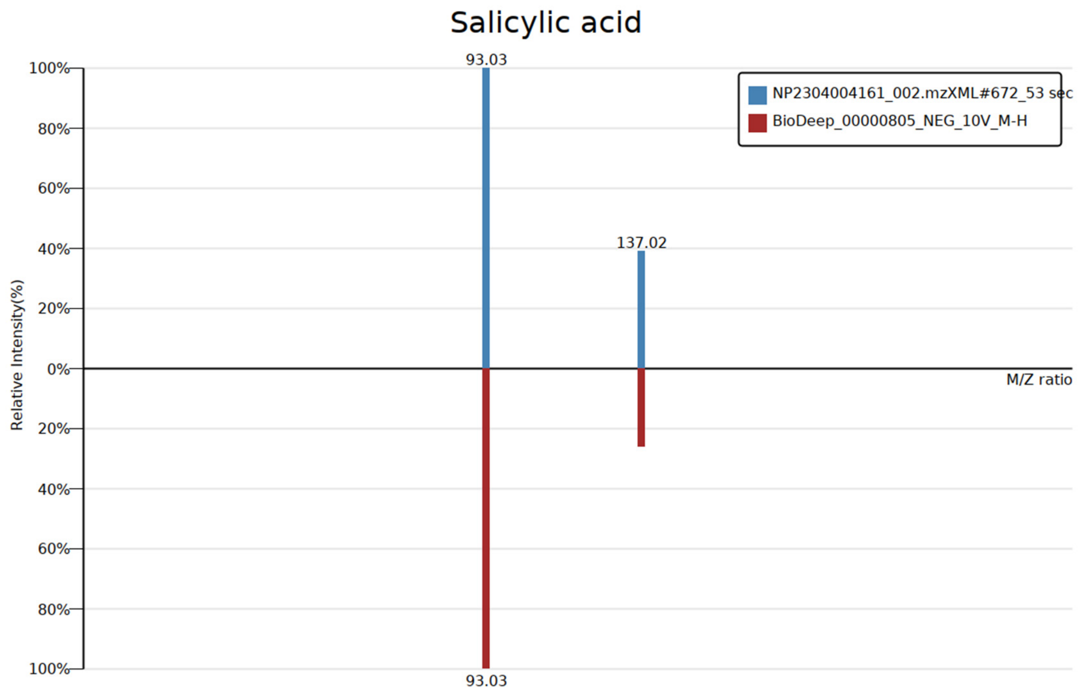

2-Carboxybenzaldehyde

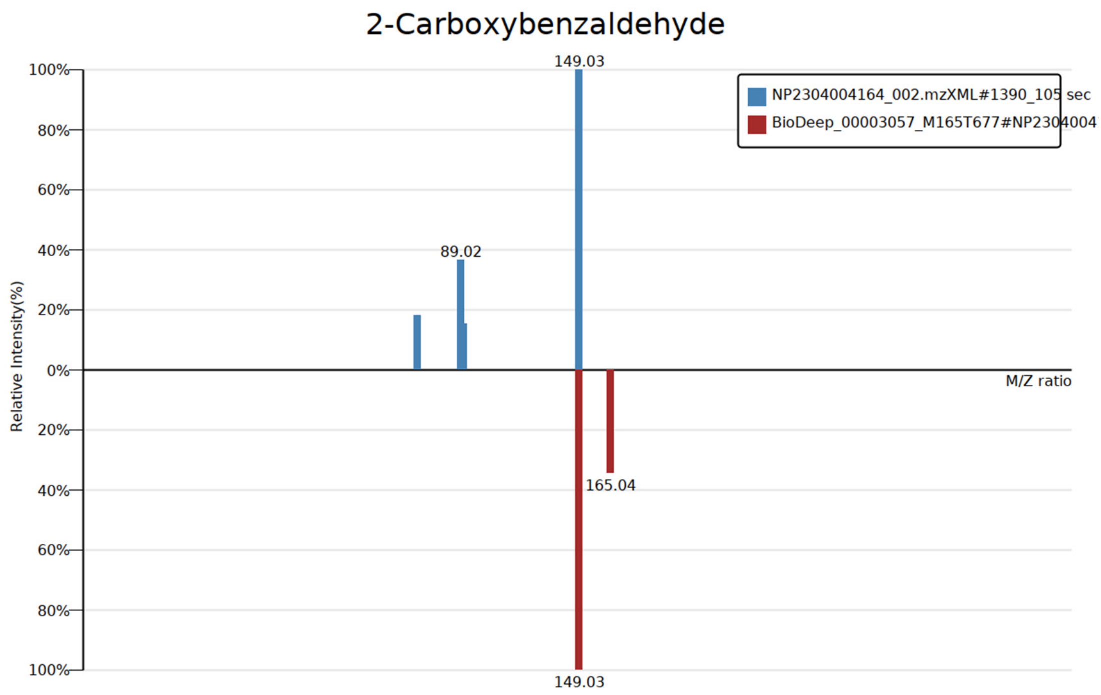

Phthalic acid

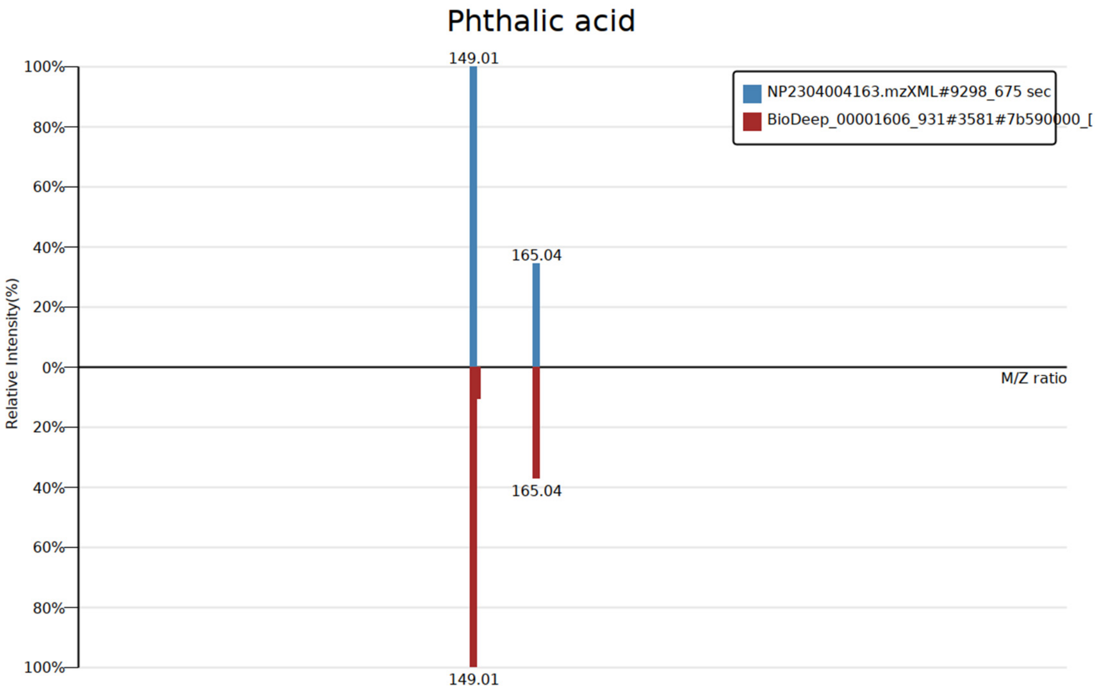

4-Quinolinecarboxylic acid

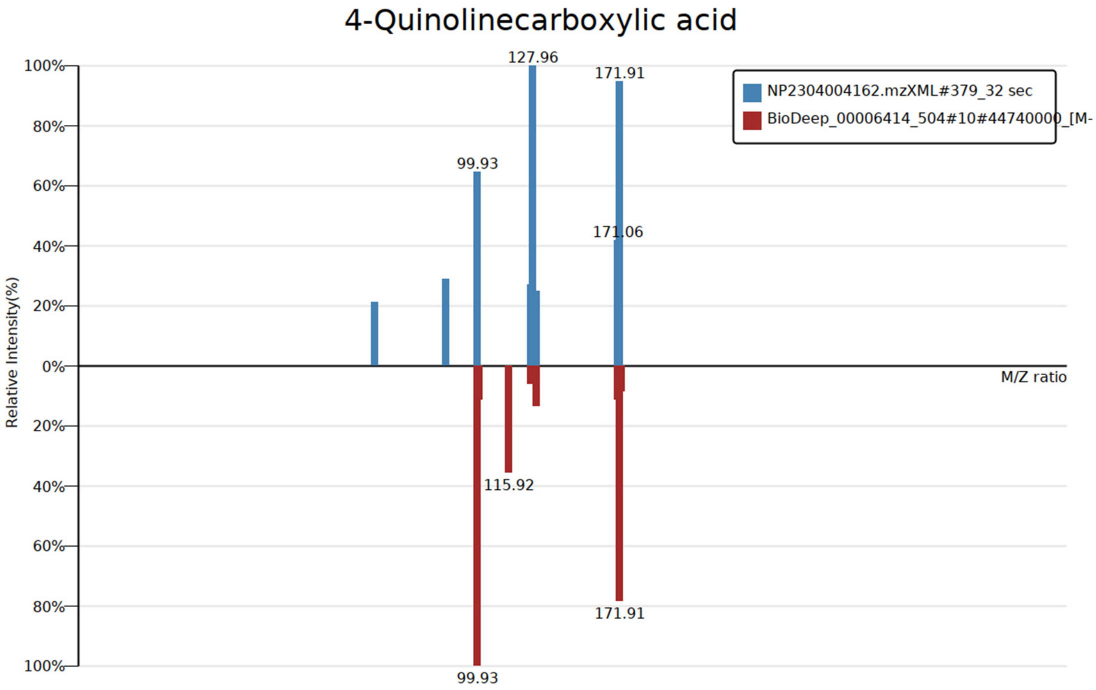

Suberic acid

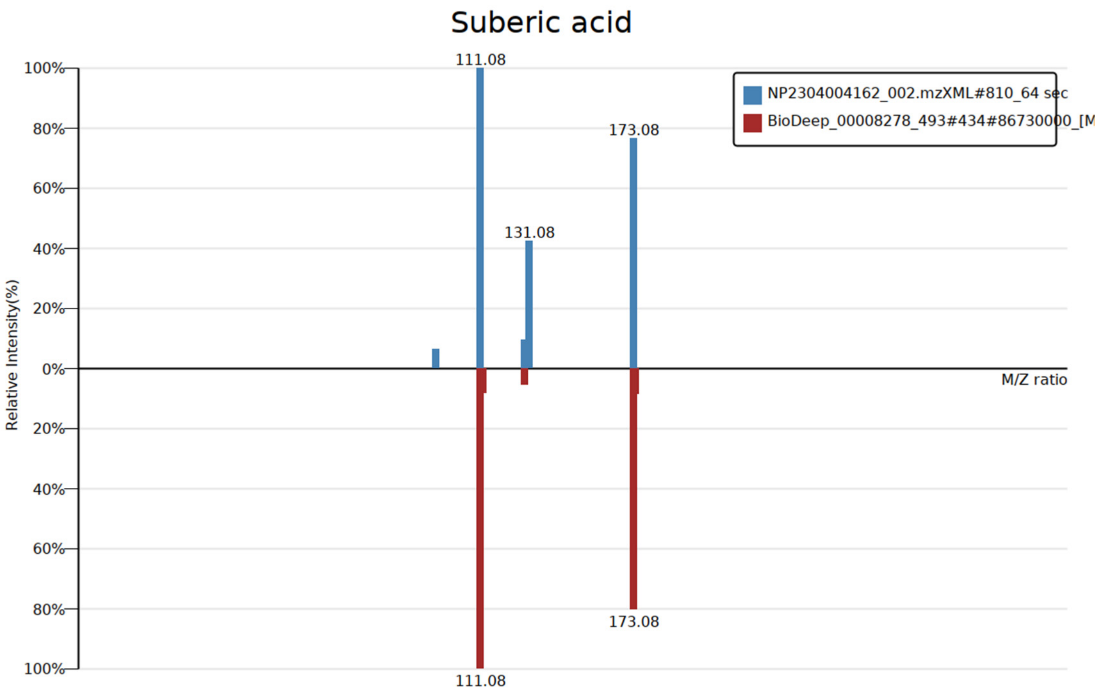

Azelaic acid

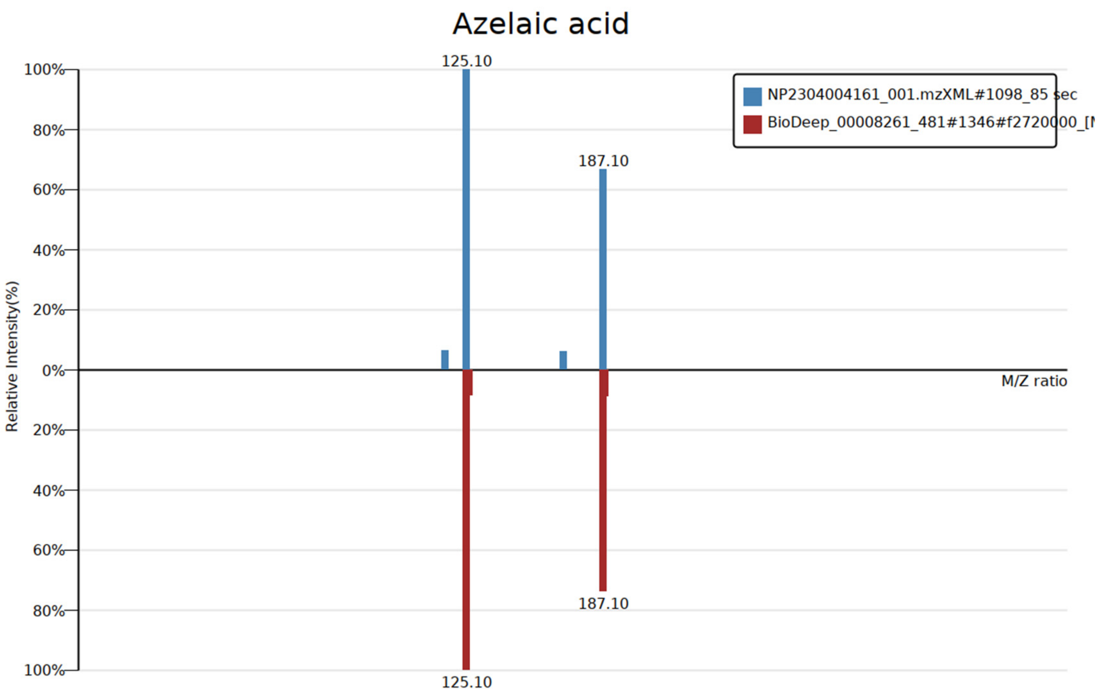

Quinate

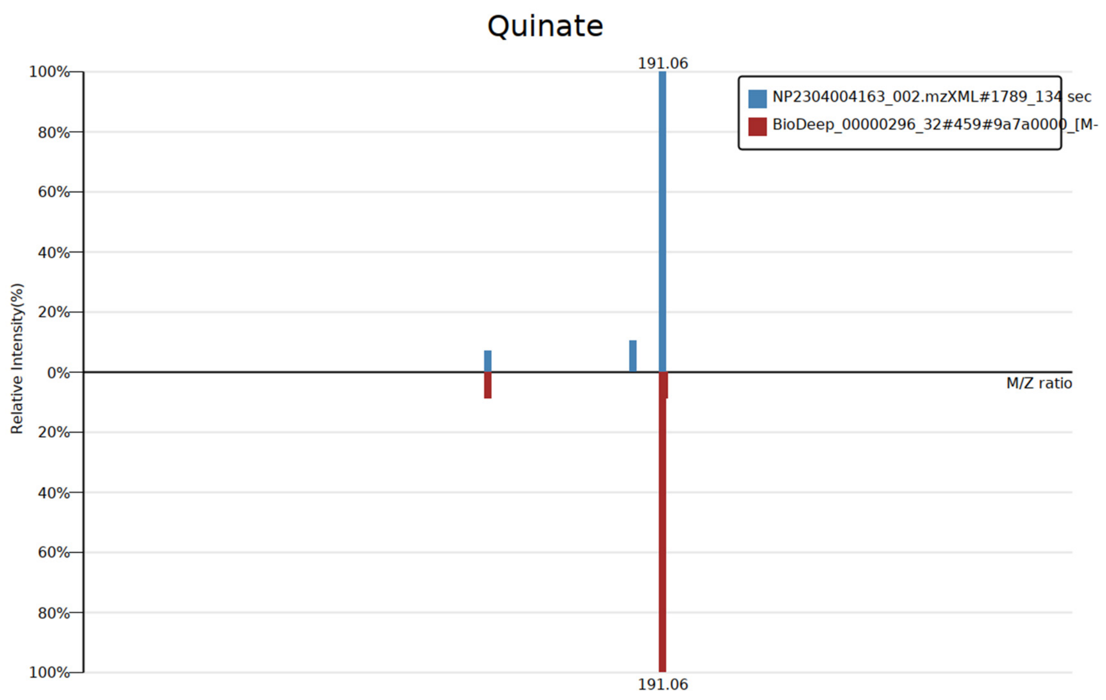

Citric acid

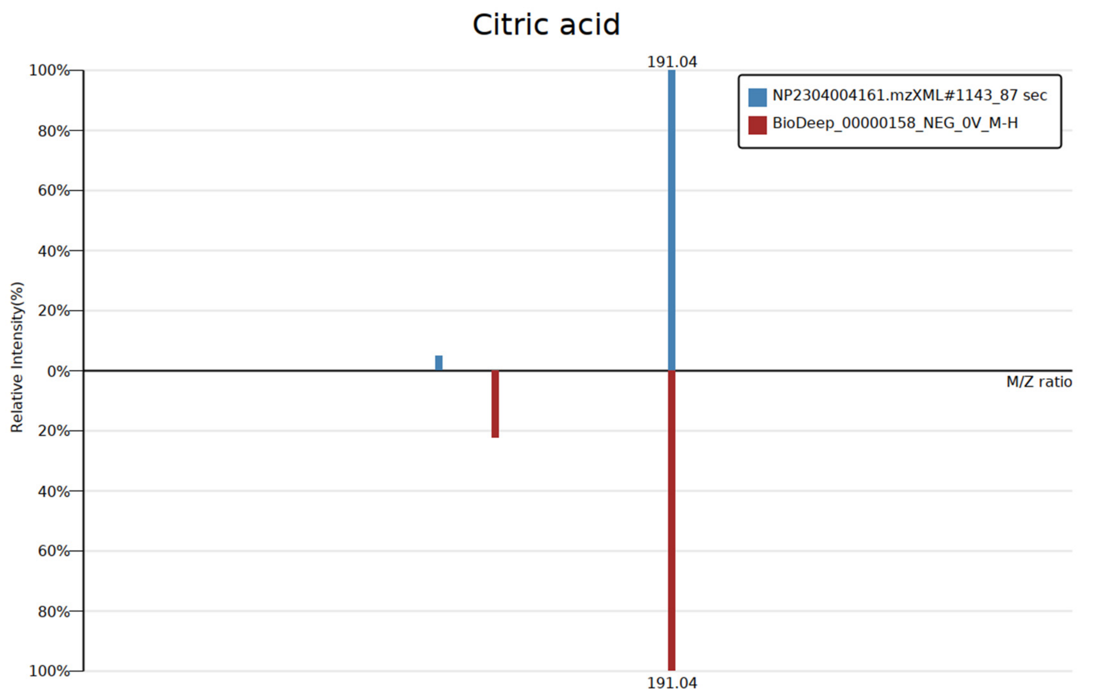

3-Oxalomalate

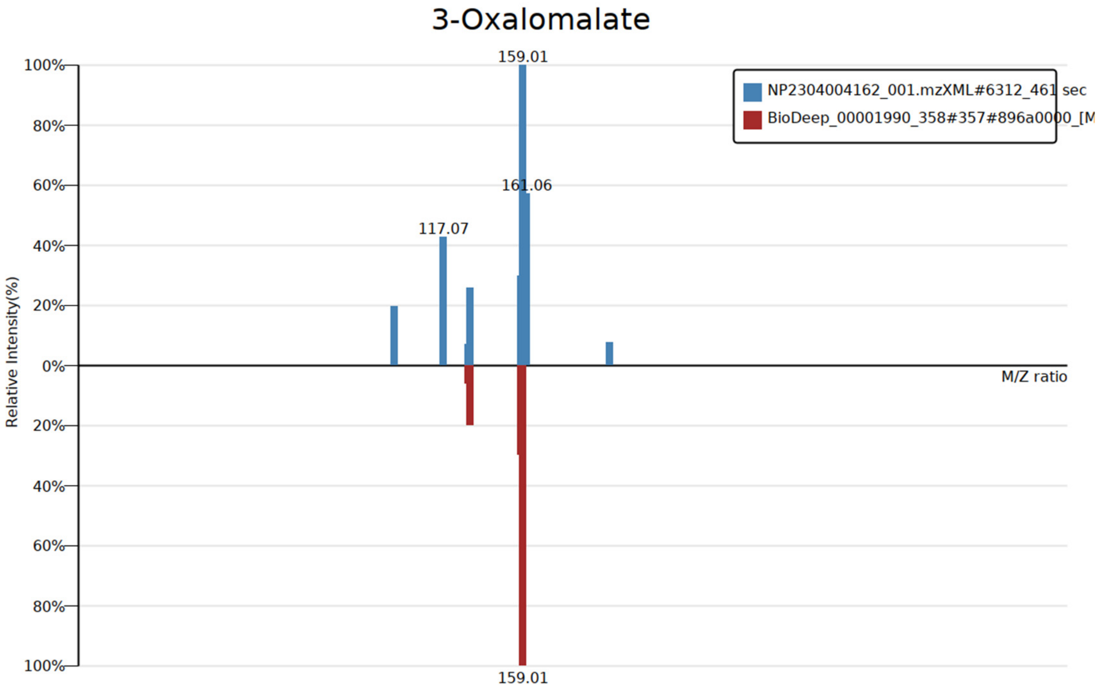

Galactaric acid

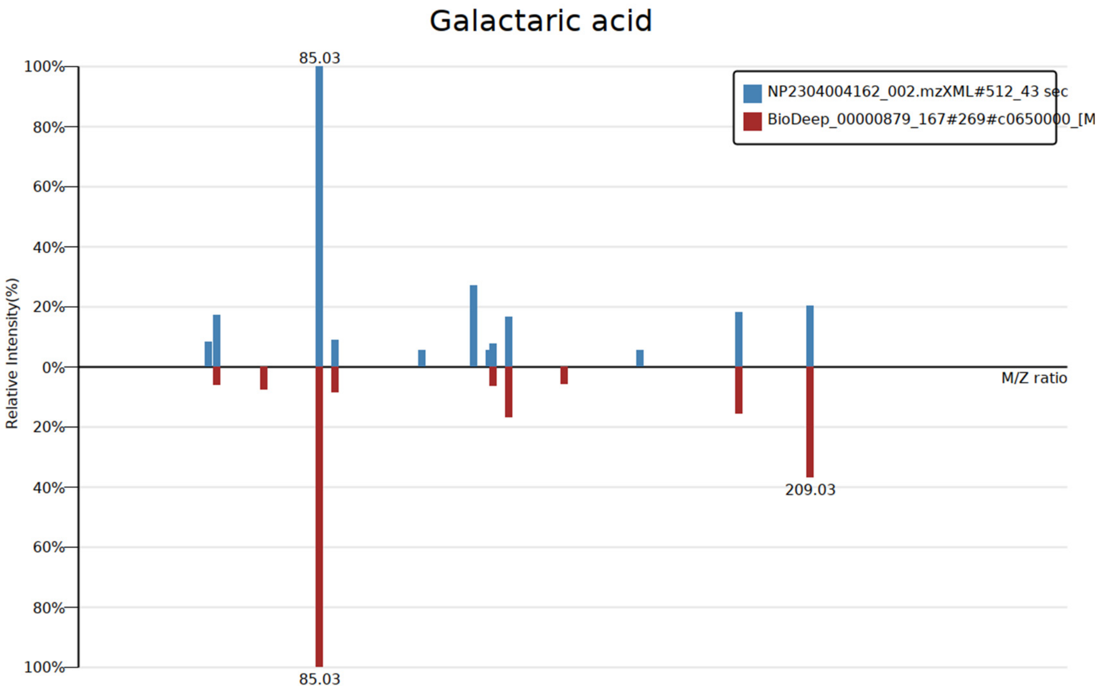

Dodecanedioic acid

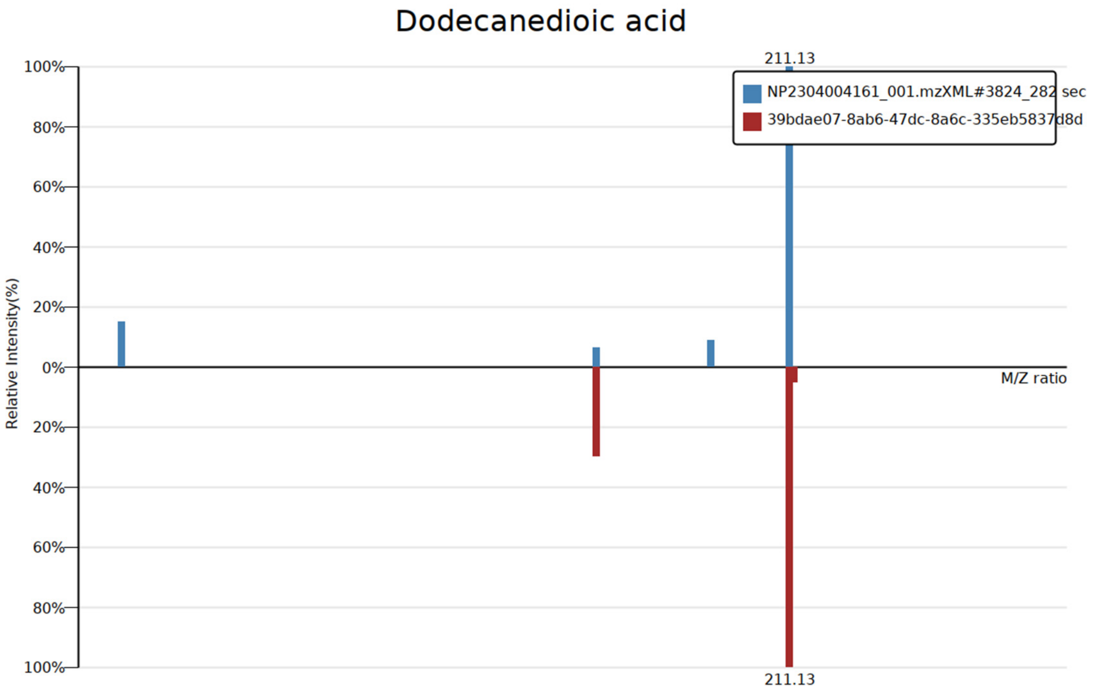

5-Nitro-2-(3-phenylpropylamino)benzoic acid

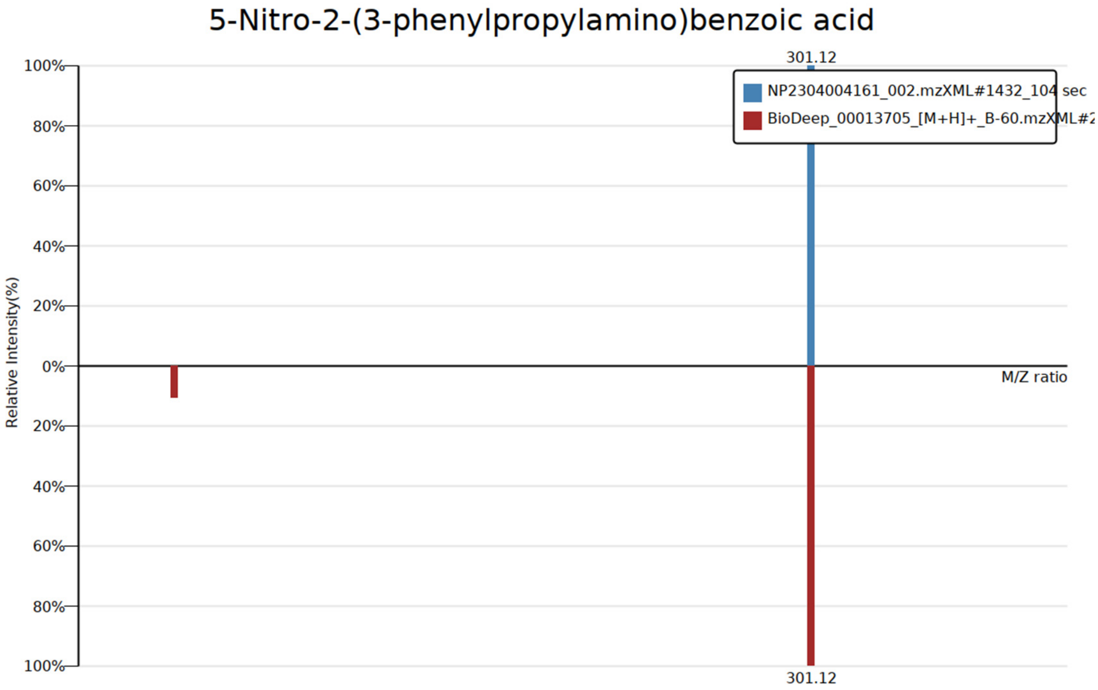

Phenylpyruvic acid

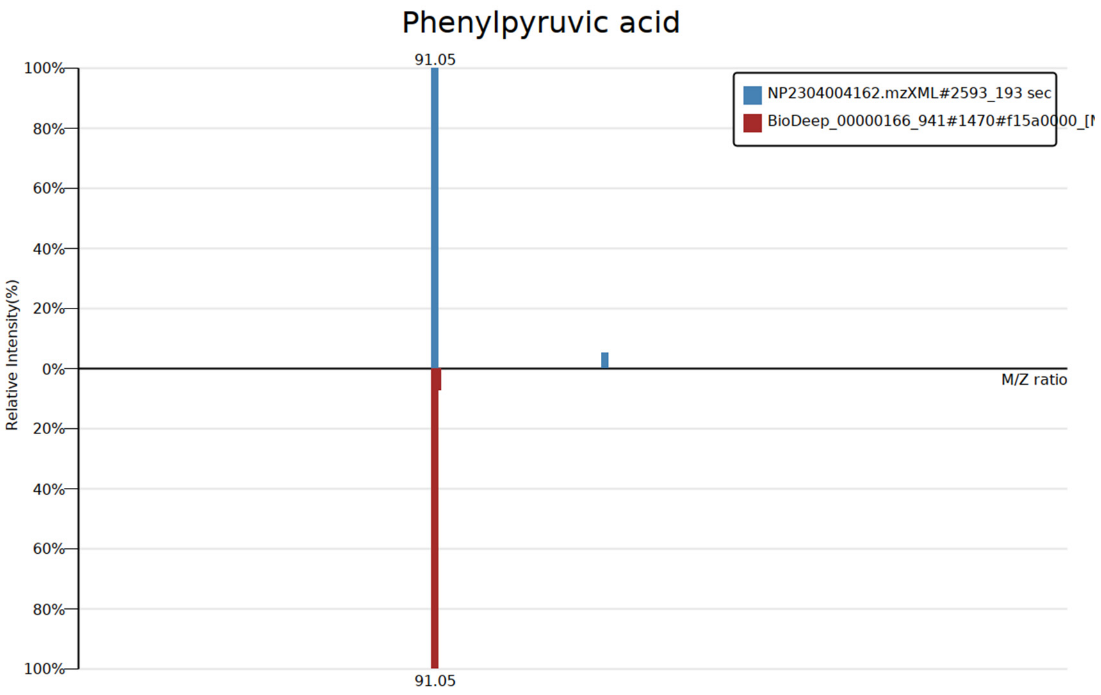

2-Carboxybenzaldehyde

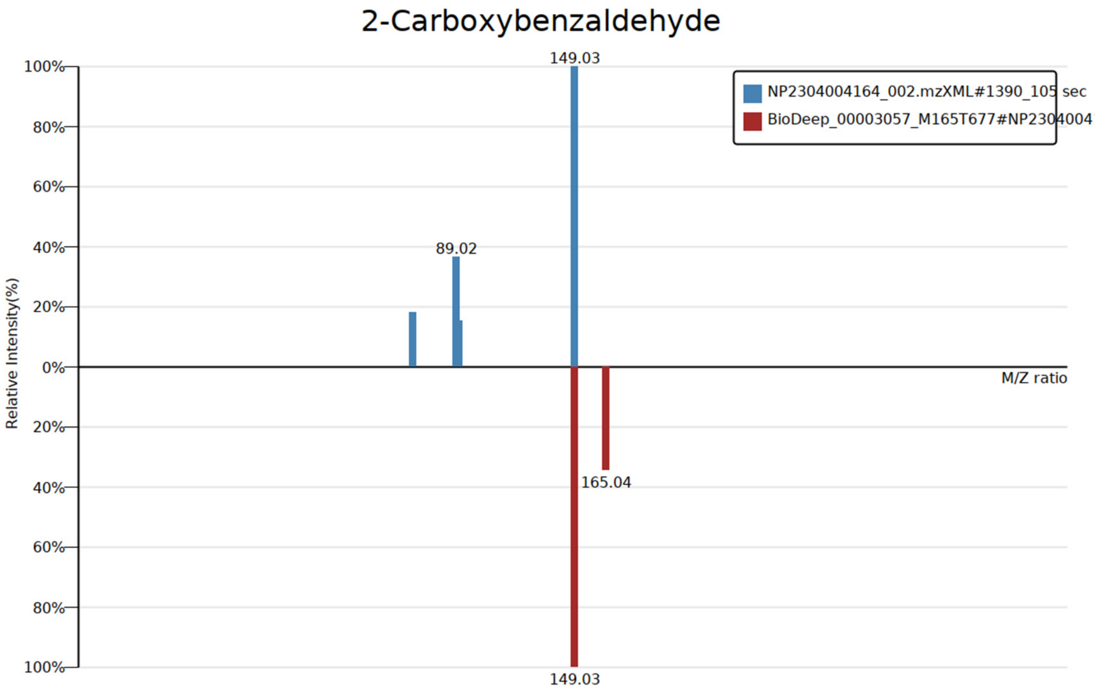

9,10-Epoxyoctadecenoic acid

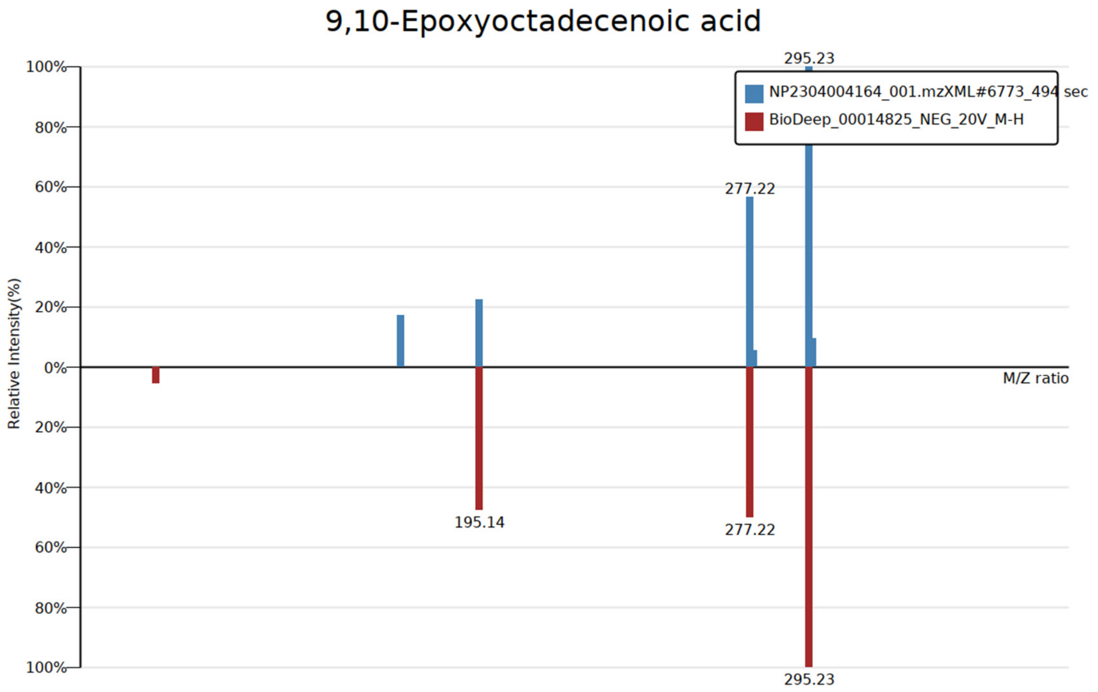

8(R)-Hydroperoxylinoleic acid

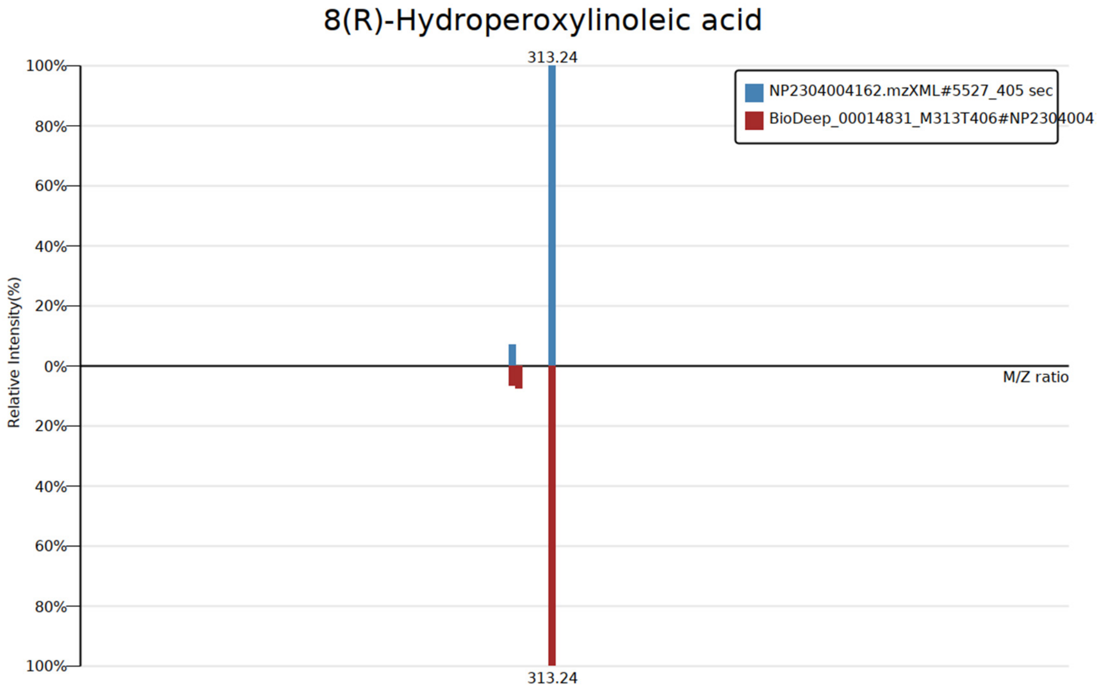

11,12-DiHETrE

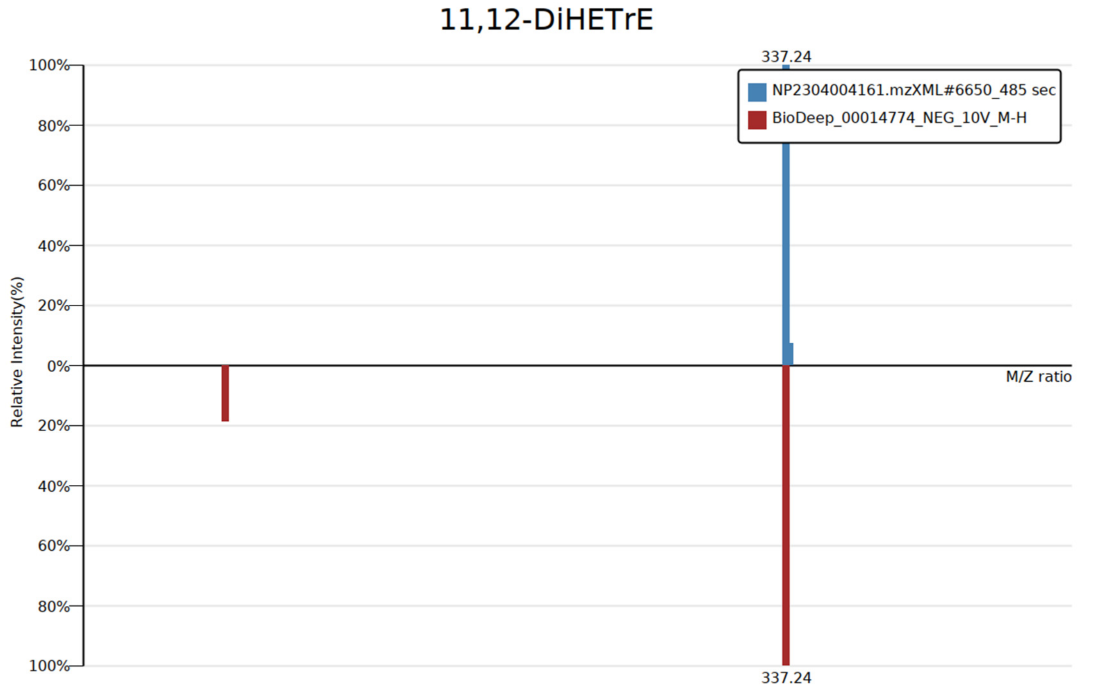

Pelargonic acid

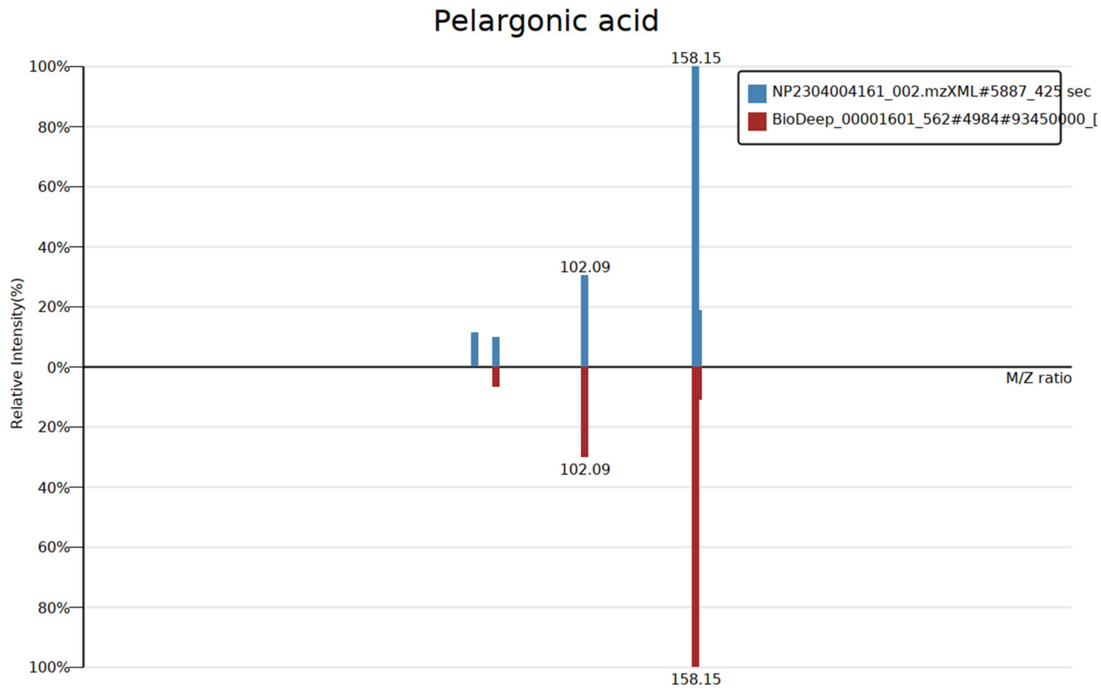

Undecanoic acid

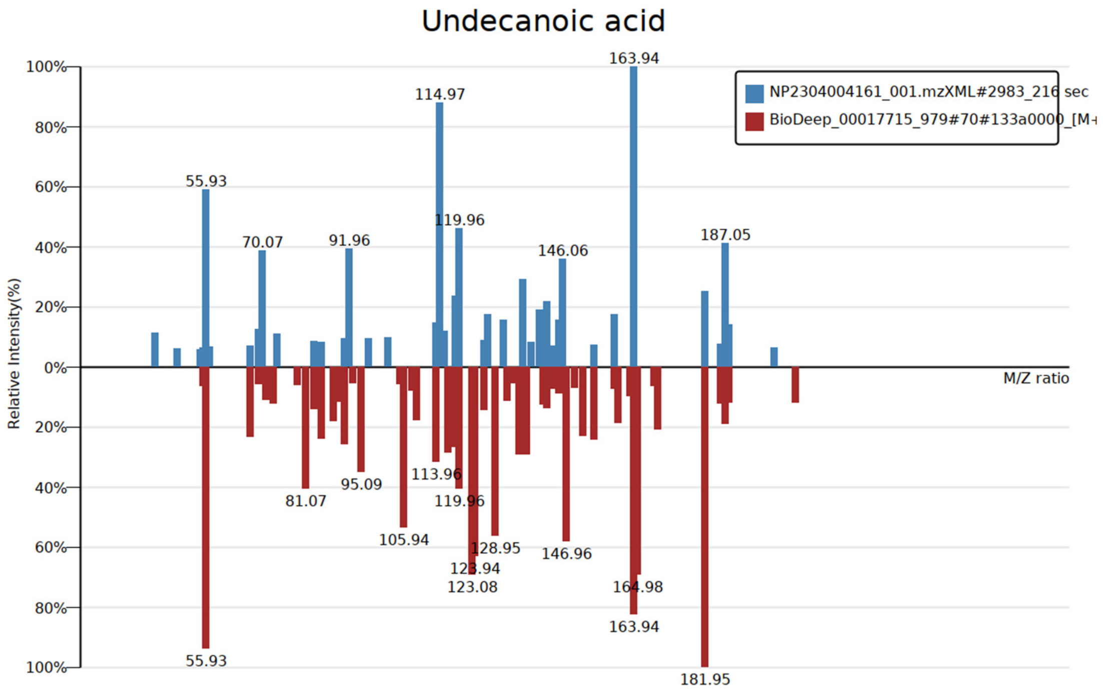

Vaccenic acid

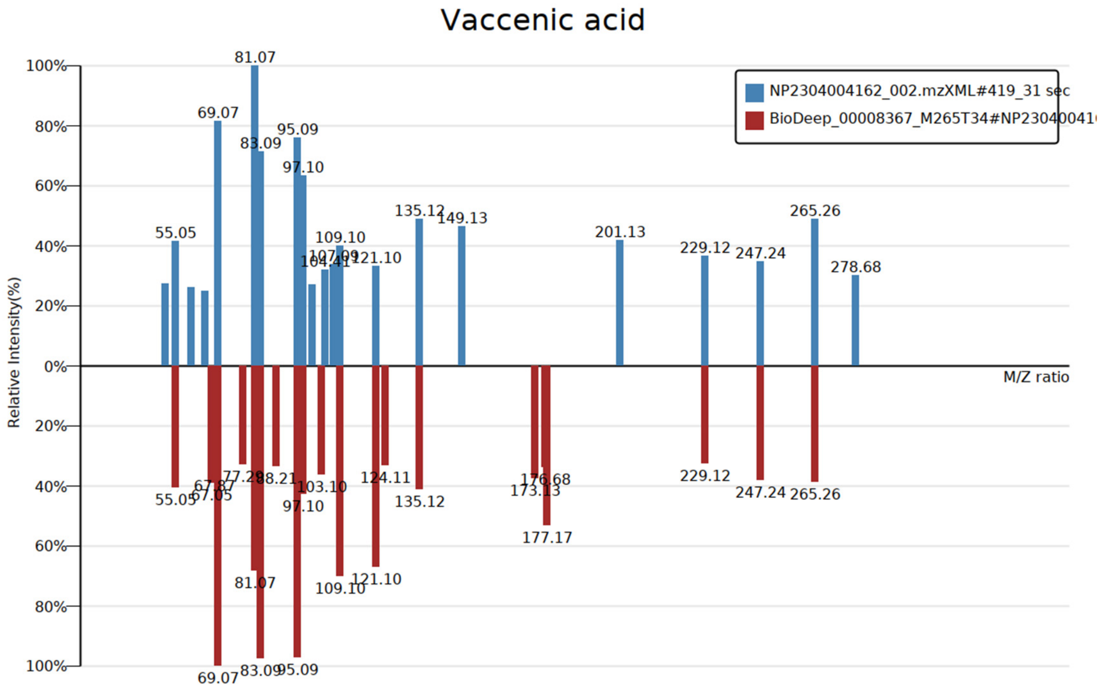

Palmitic acid

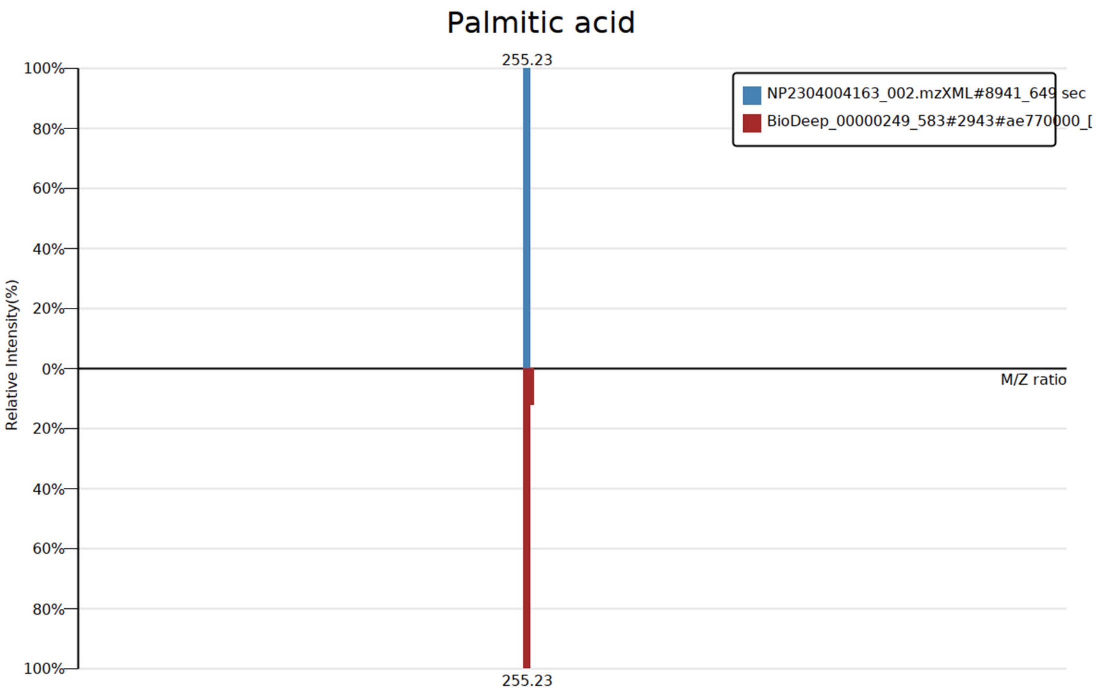

16-Hydroxy hexadecanoic acid

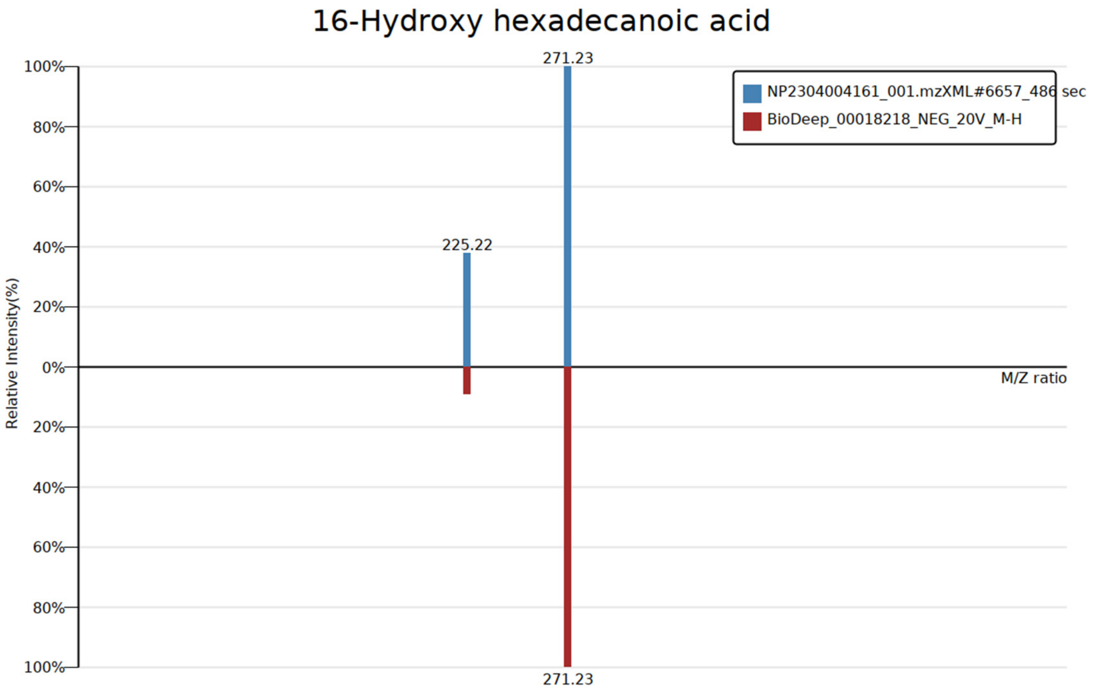

Gamma-Linolenic acid

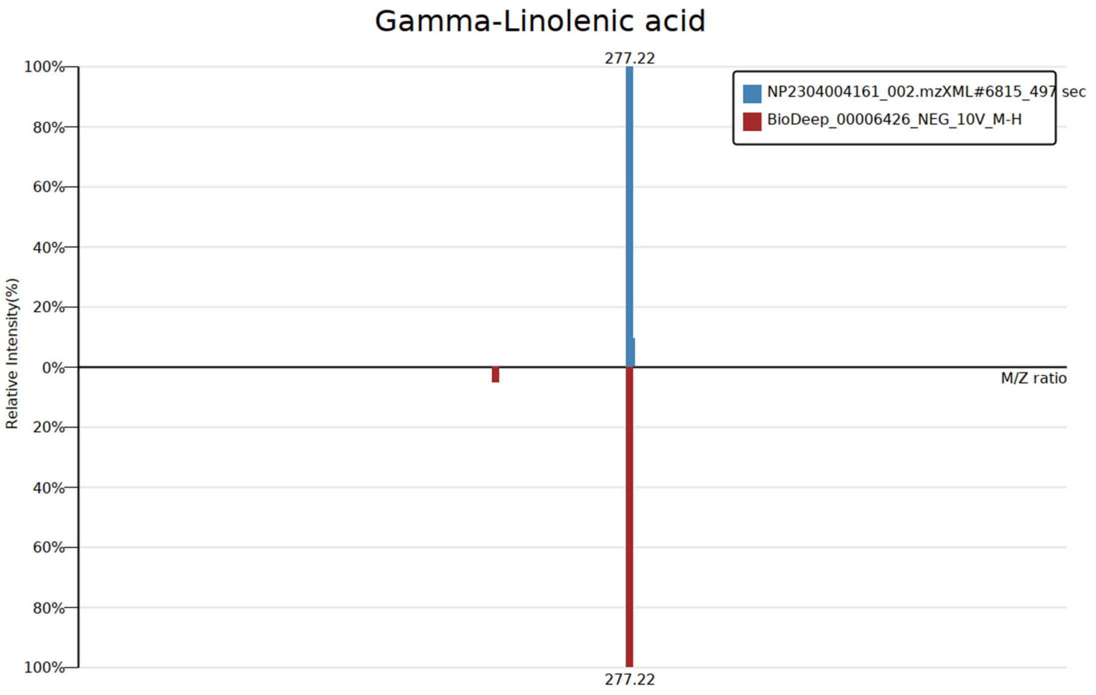

8,11,14-Eicosatrienoic acid

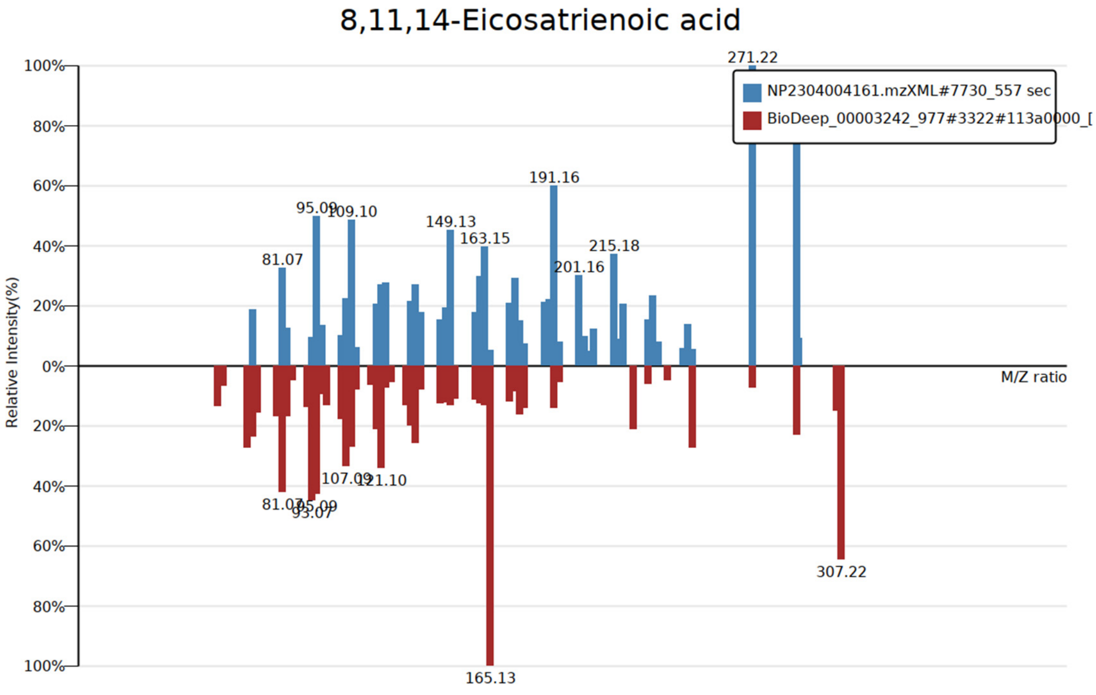

Arachidic acid

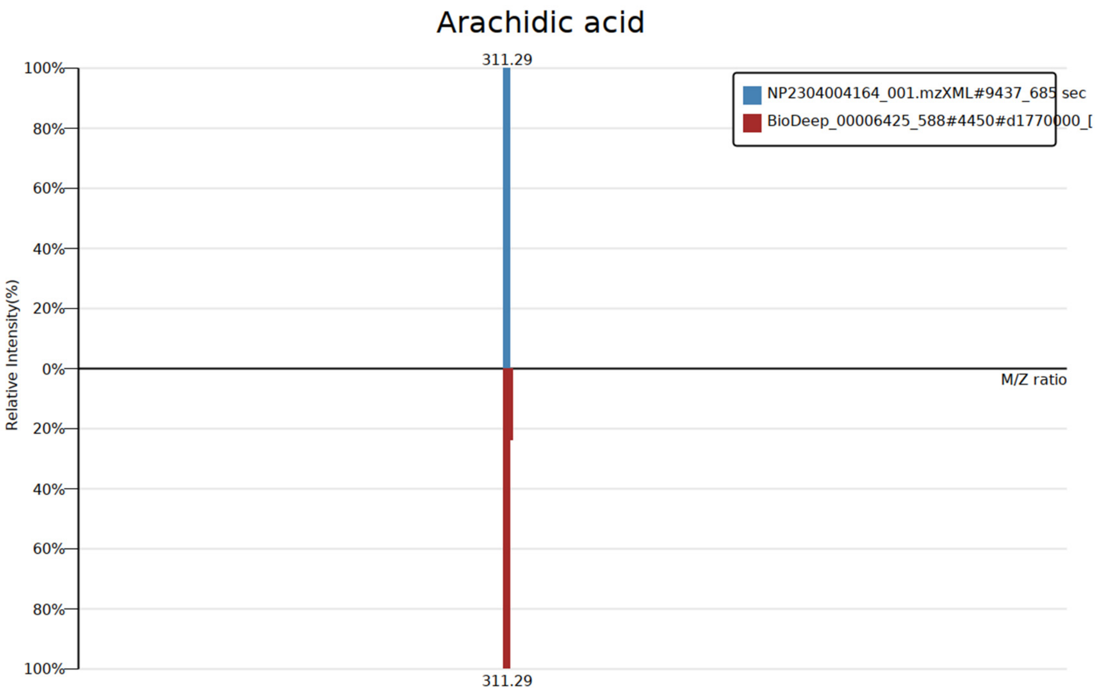

3-Ketosphingosine

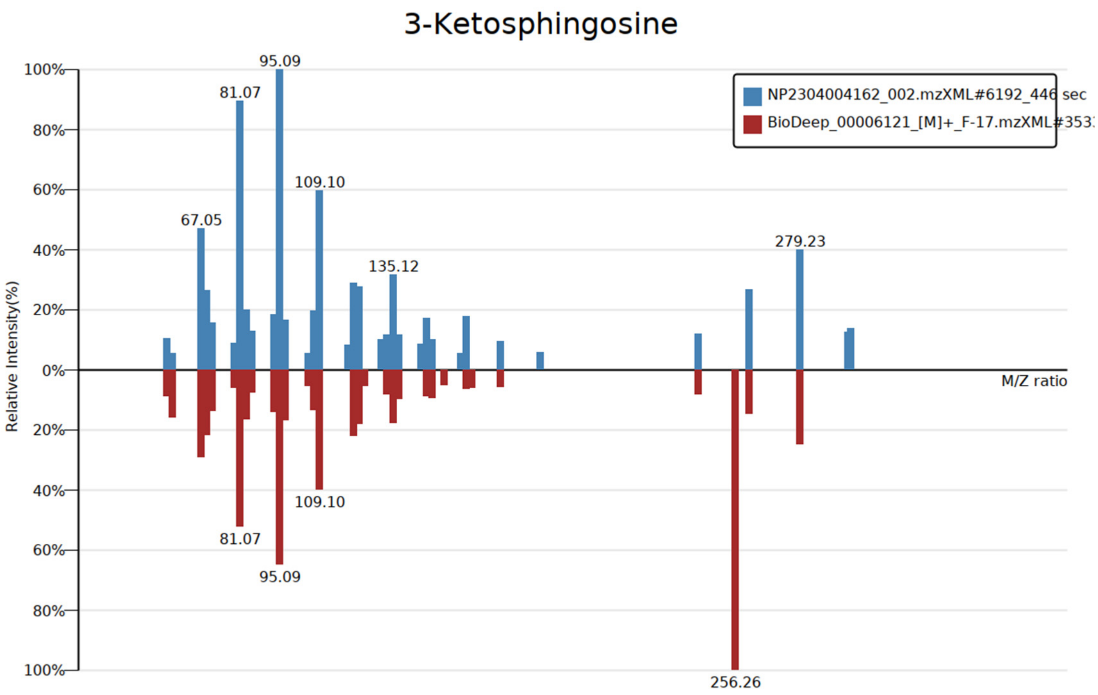

Oleoylethanolamide

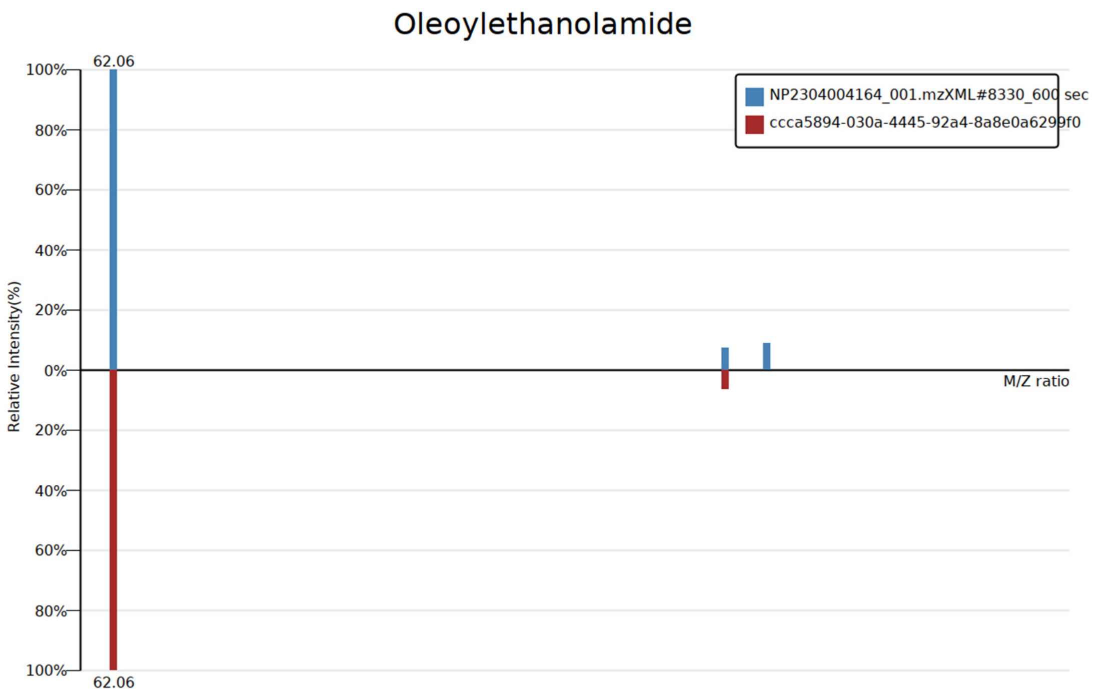

Perillyl alcohol

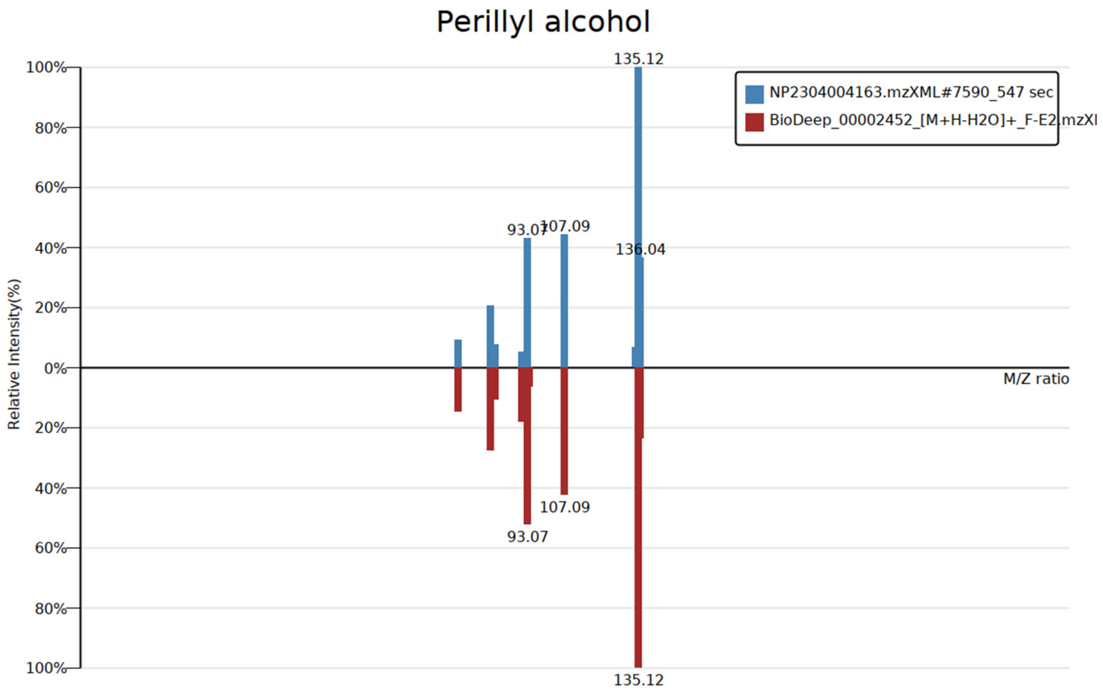

Geraniol

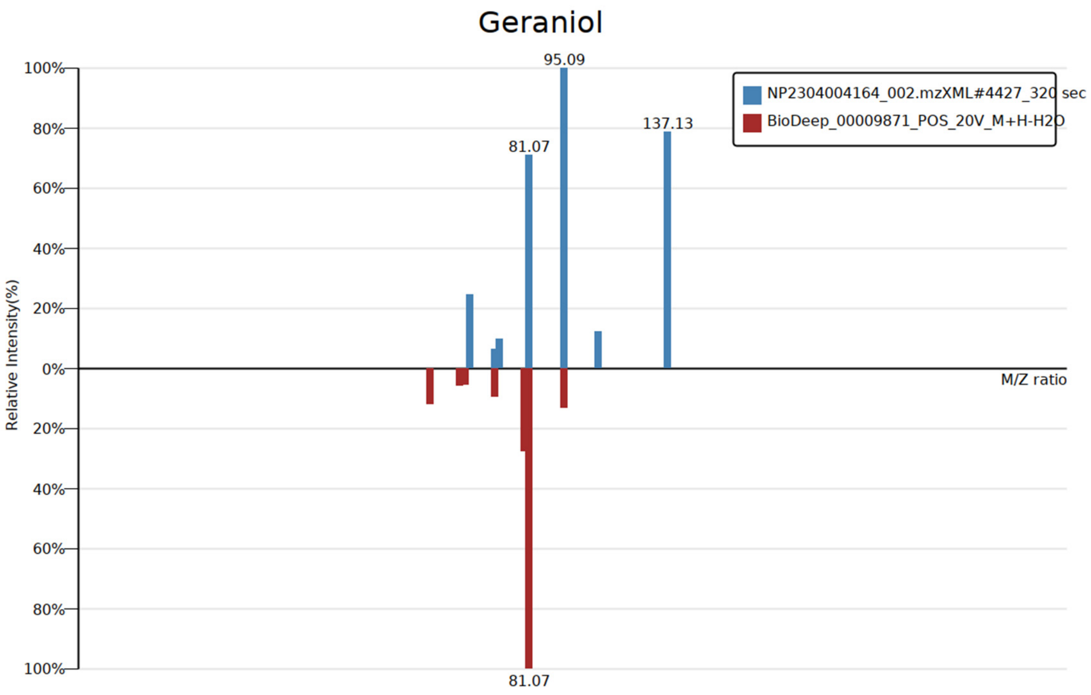

Gamma-terpinene

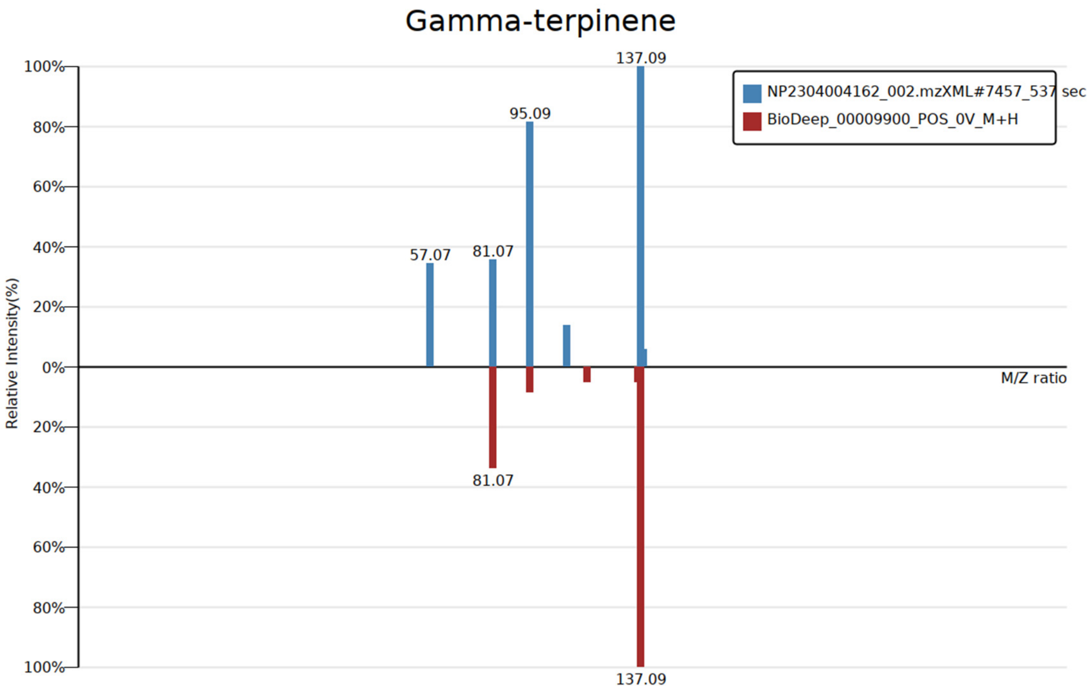

(1S,4R)-1-Hydroxy-2-oxolimonene

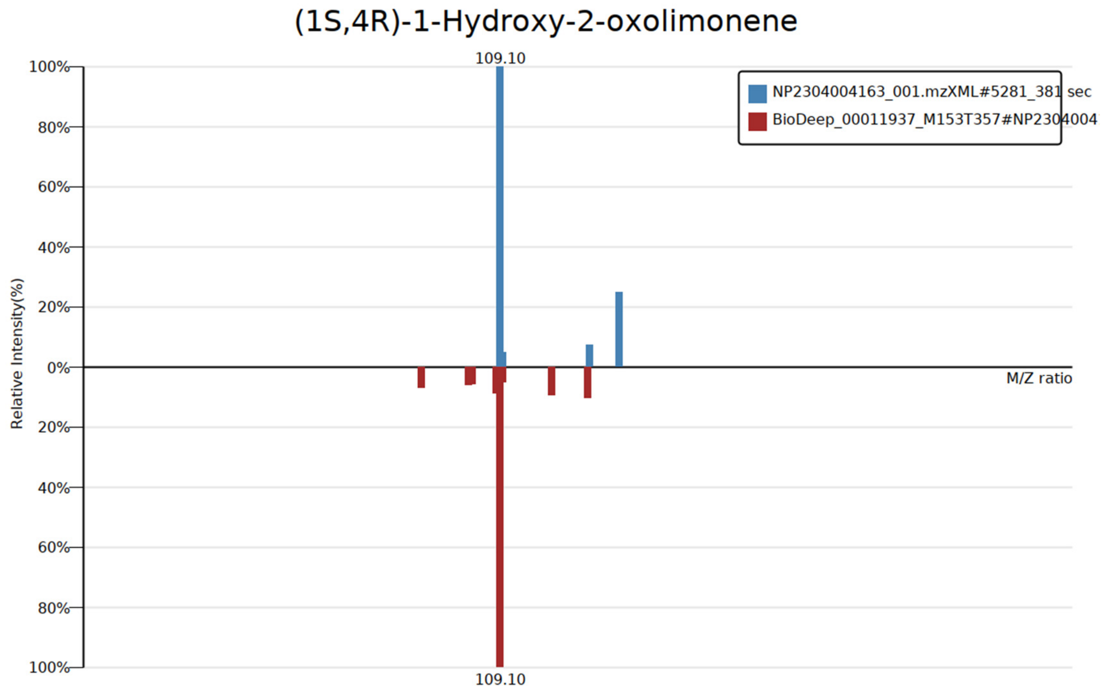

alpha-Cadinene

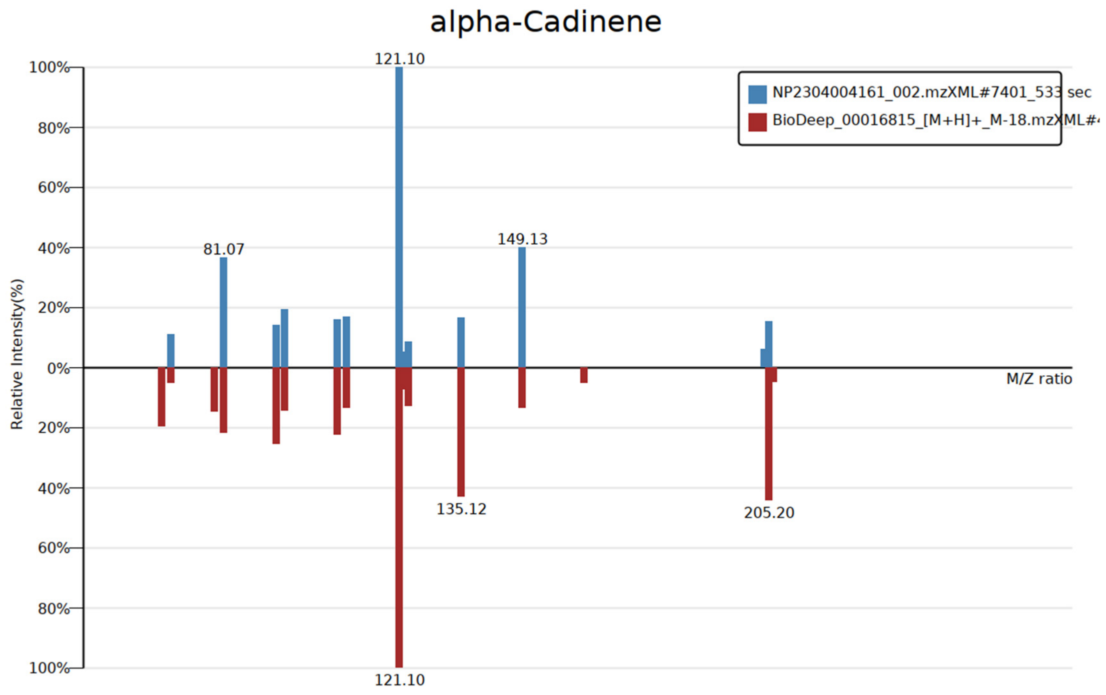

Valeranone

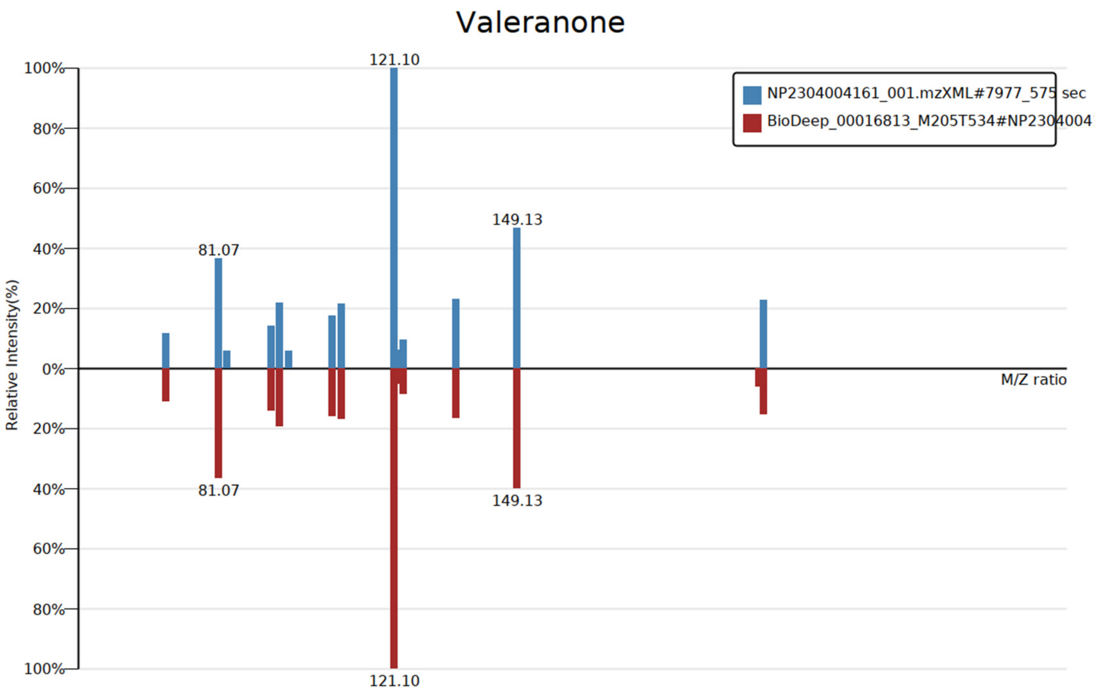

Capsidiol

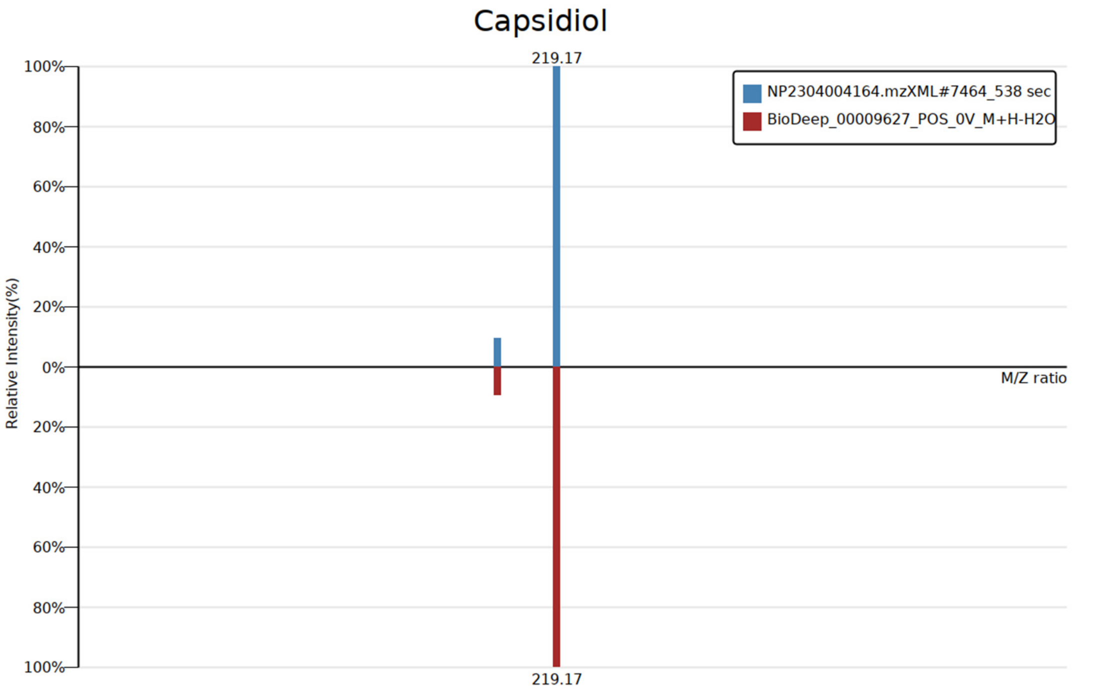

Caryophyllene alpha-oxide

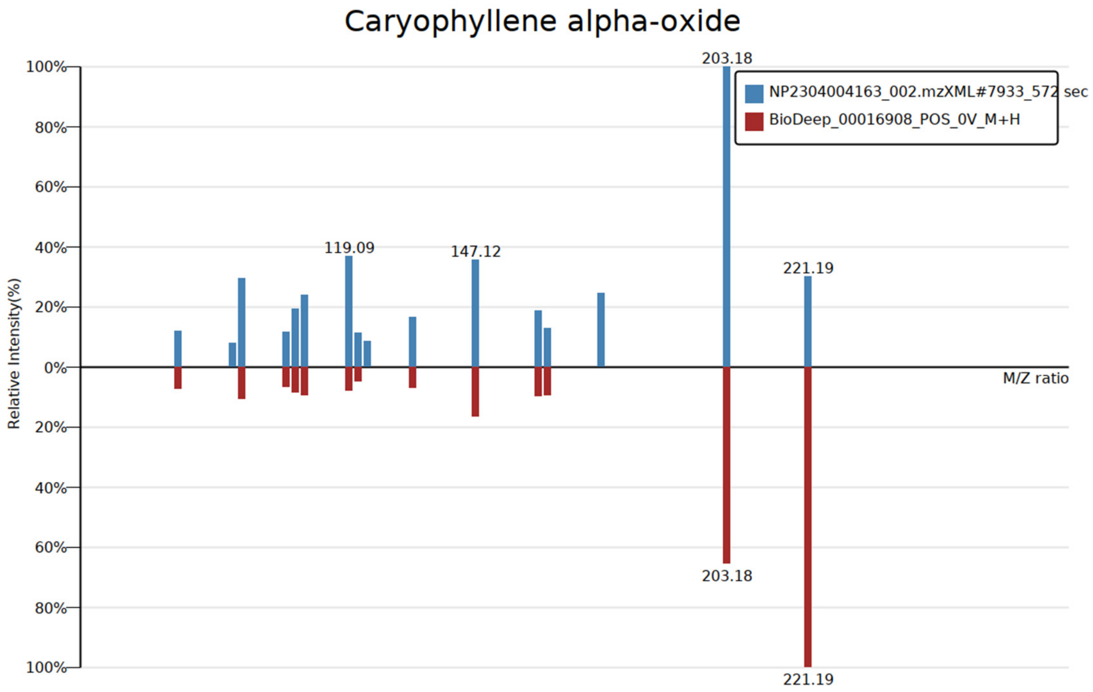

Costunolide

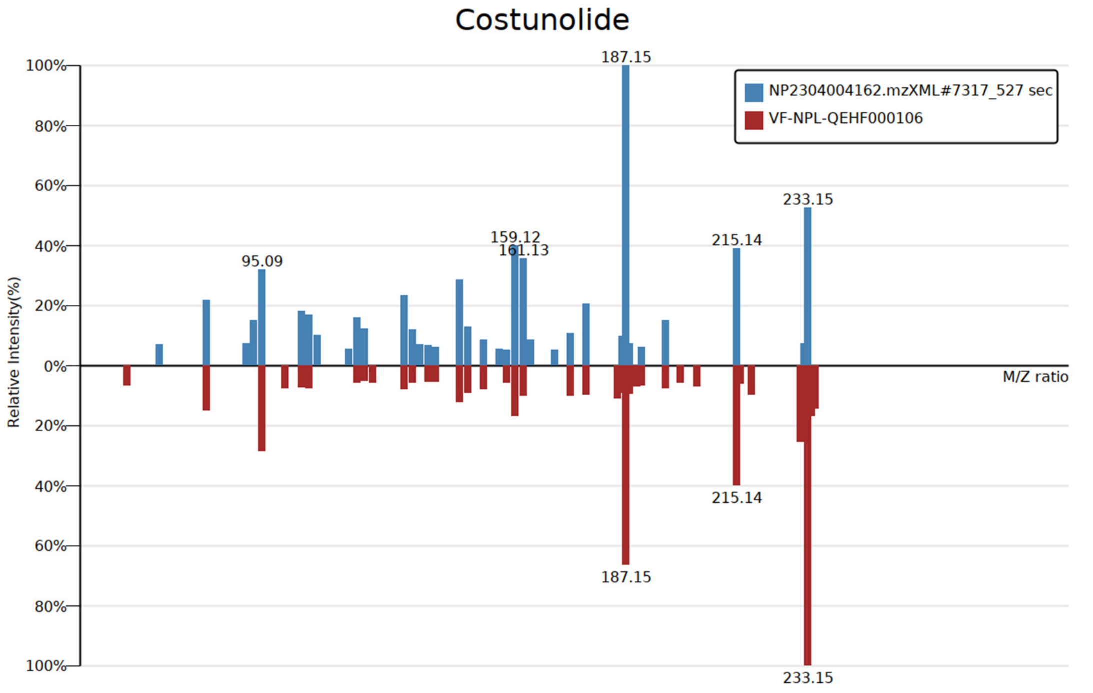

Geranyl diphosphate

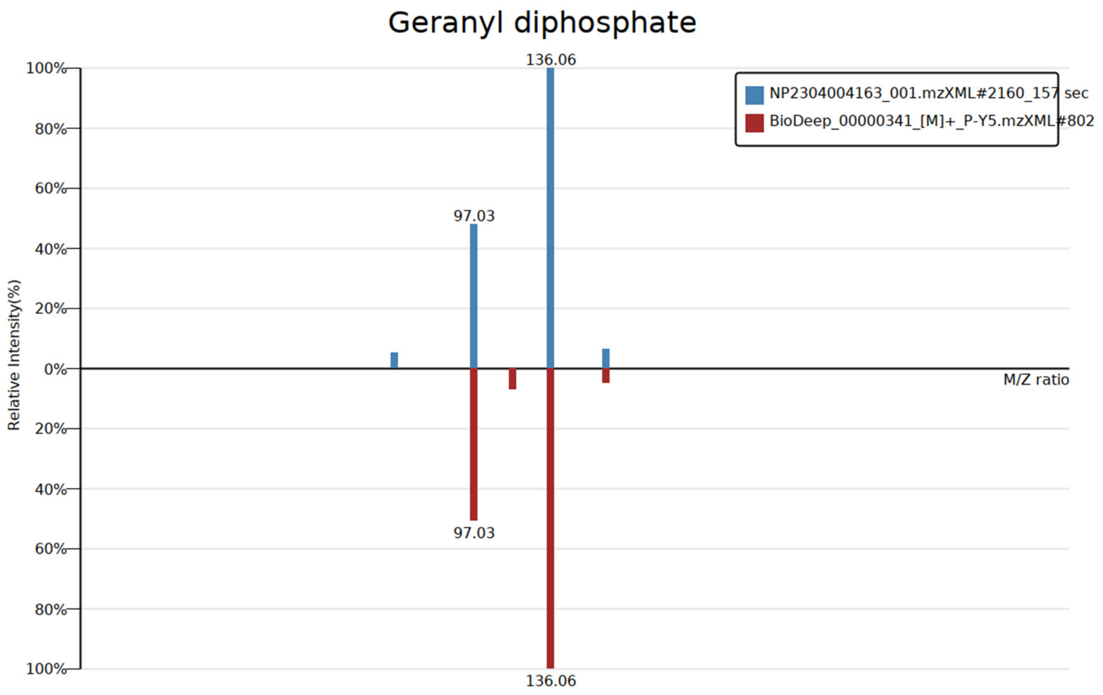

Gibberellin A7

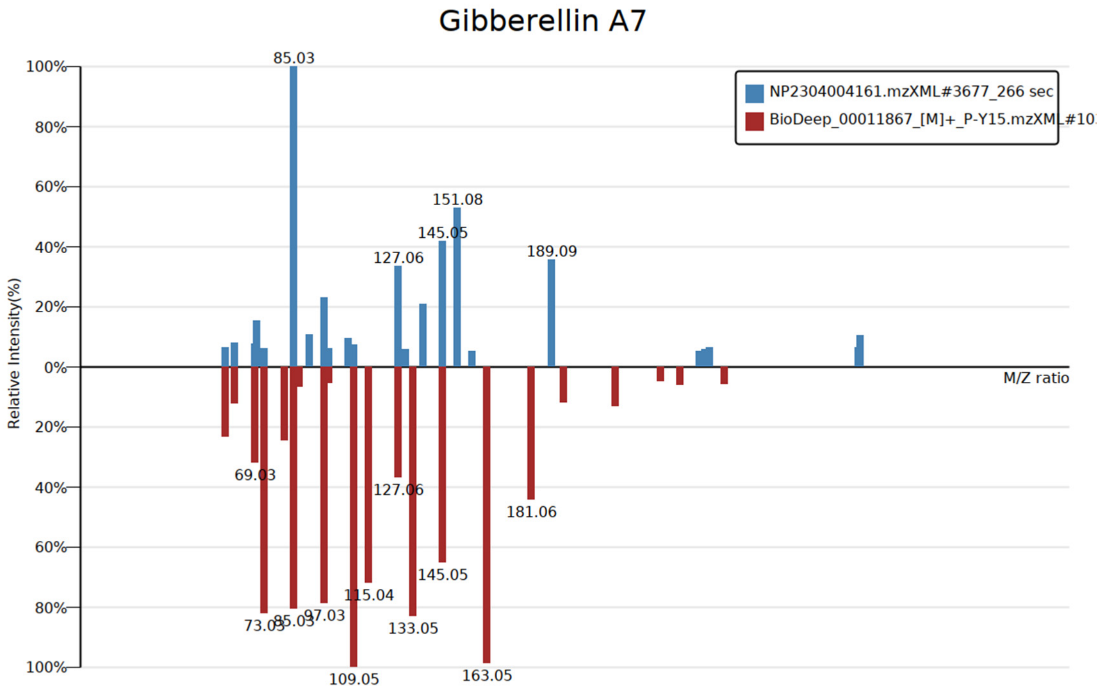

Aucubin

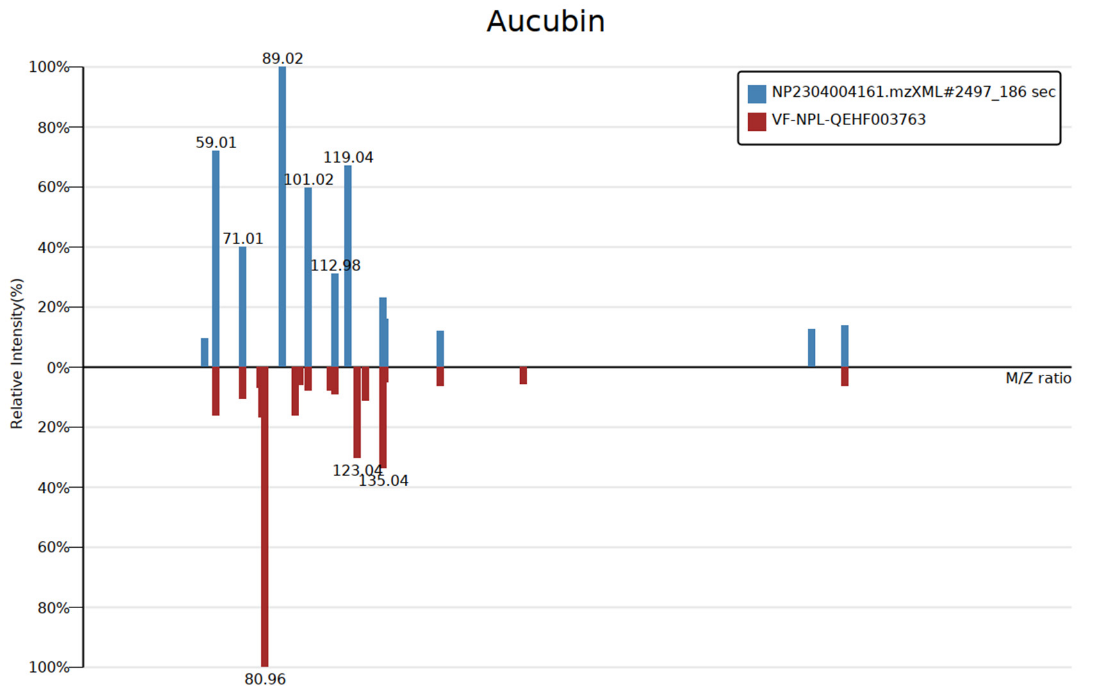

(S)-Abscisic acid

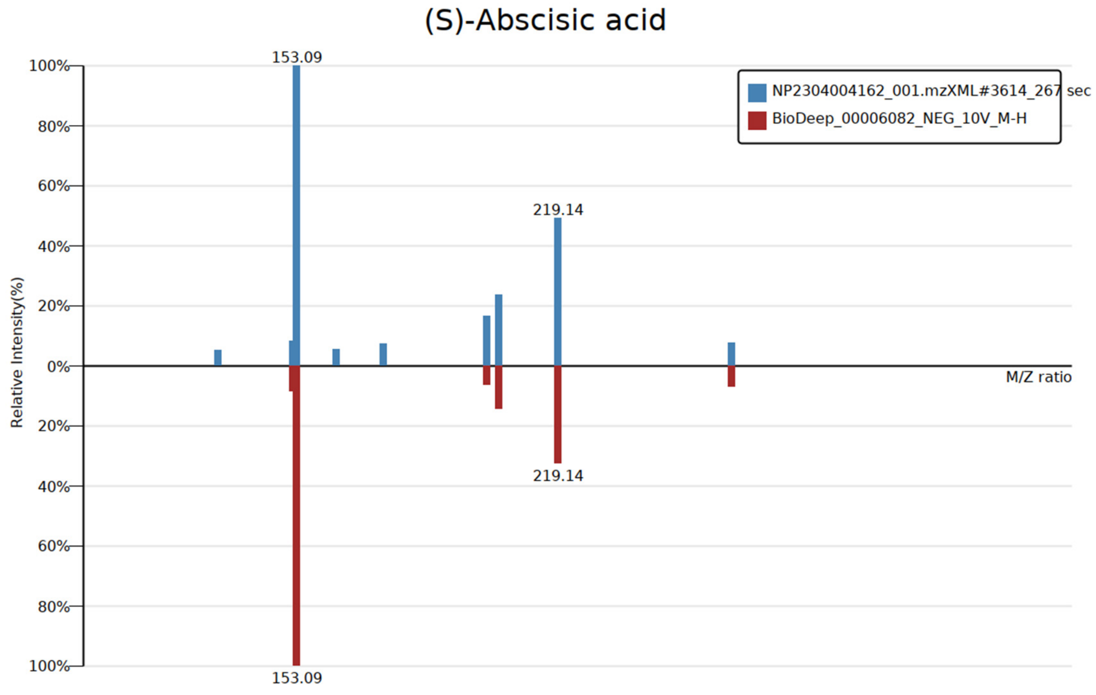

Campesterol

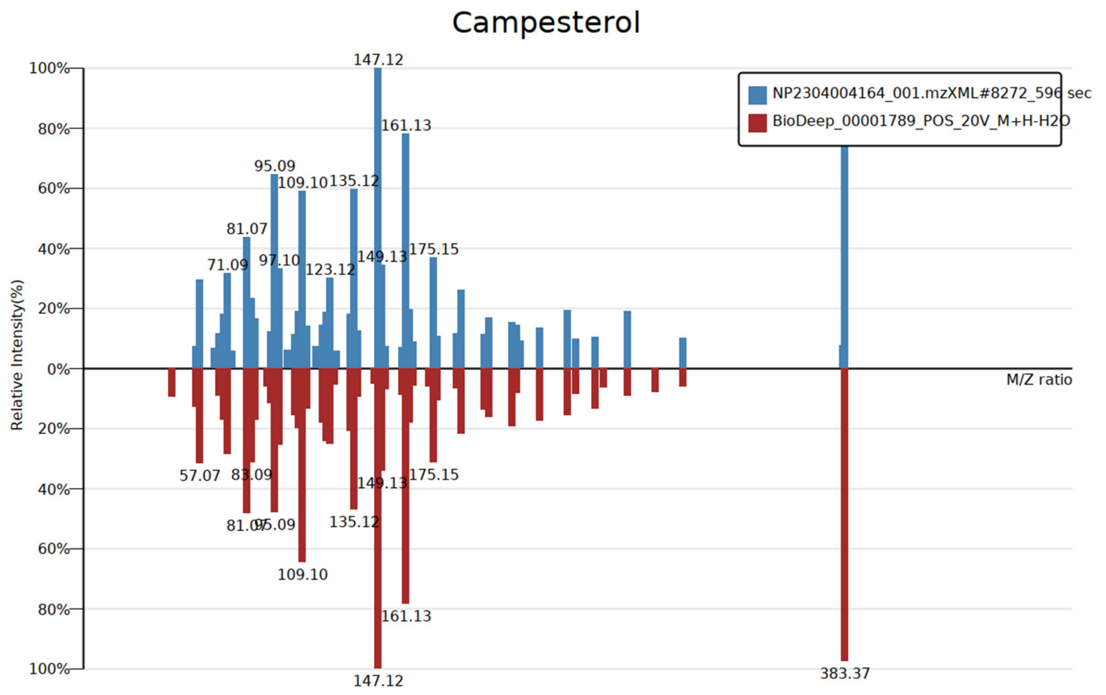

Ergosta-5,7,22,24(28)-tetraen-3beta-ol

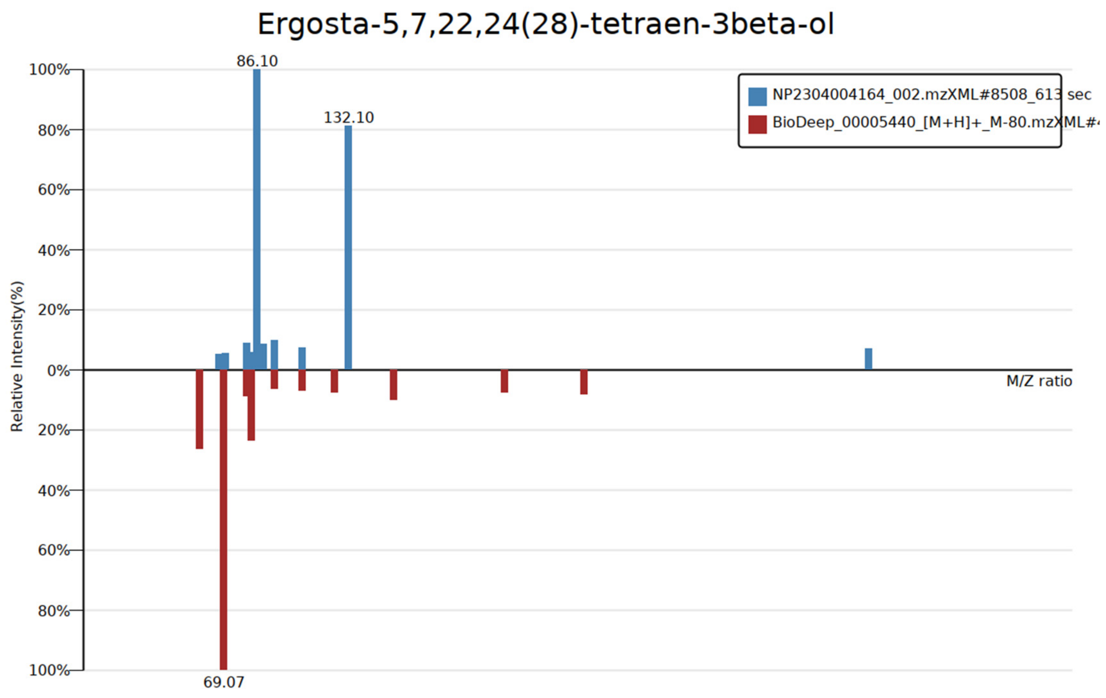

Stigmasterol

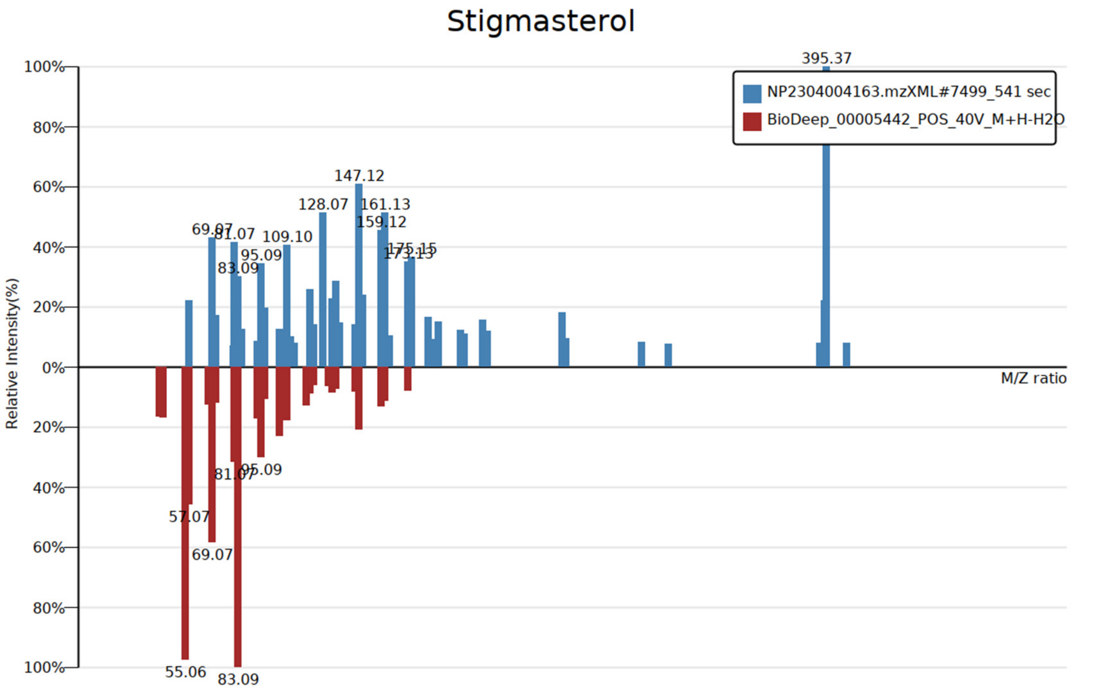

Hellebrigenin

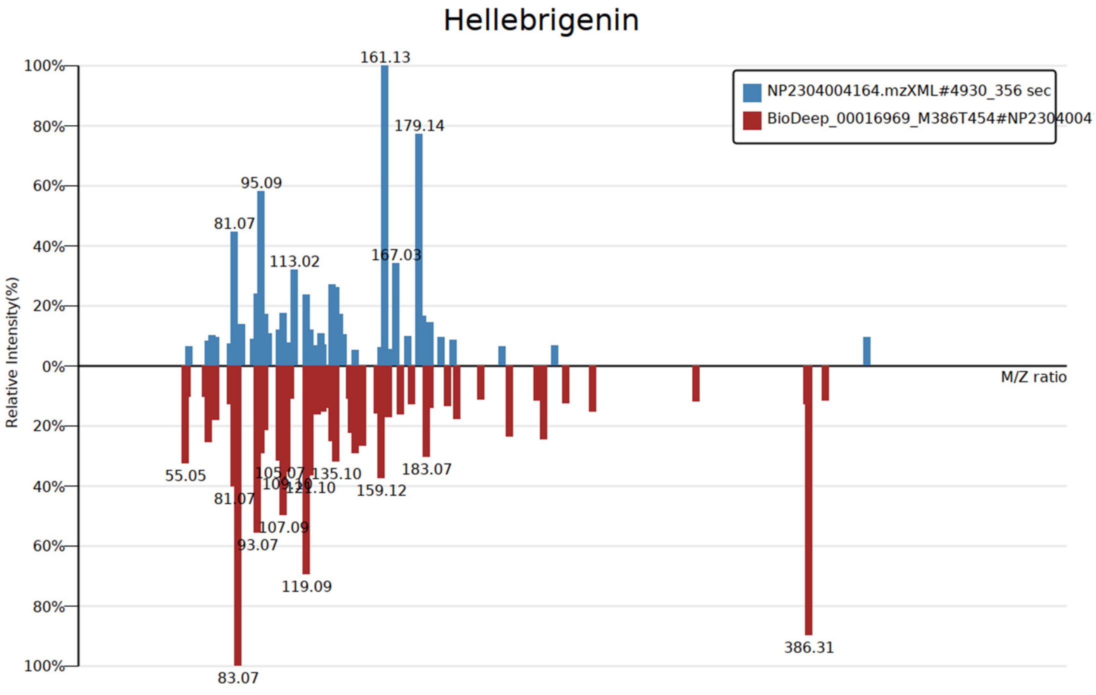

Dihydrouracil

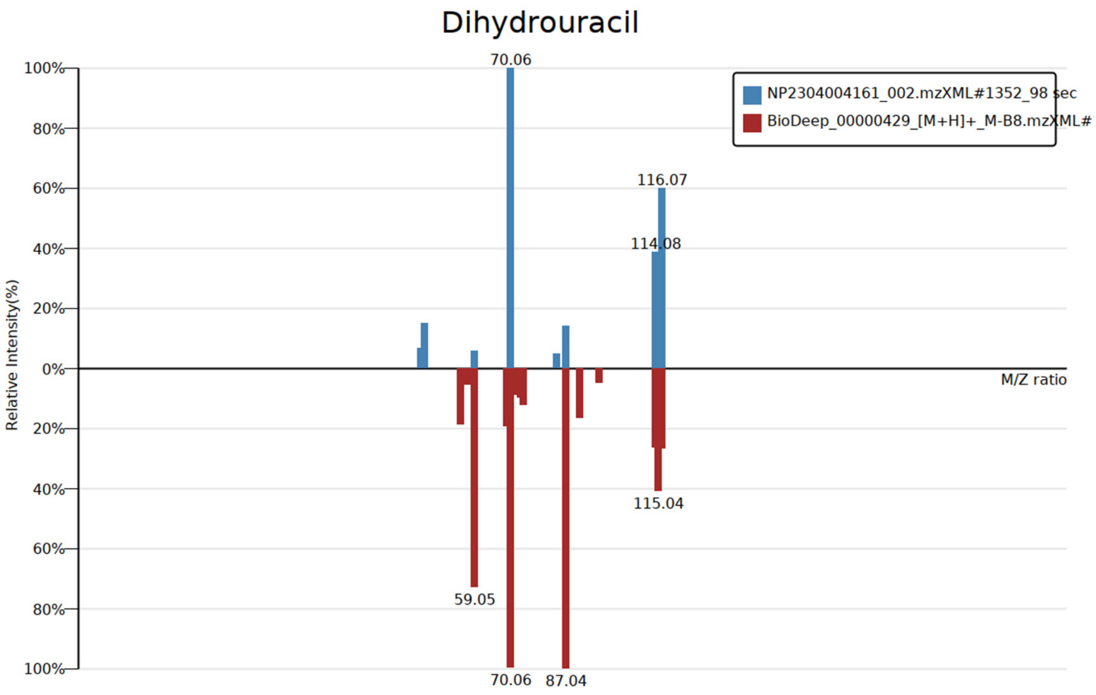

Thymine

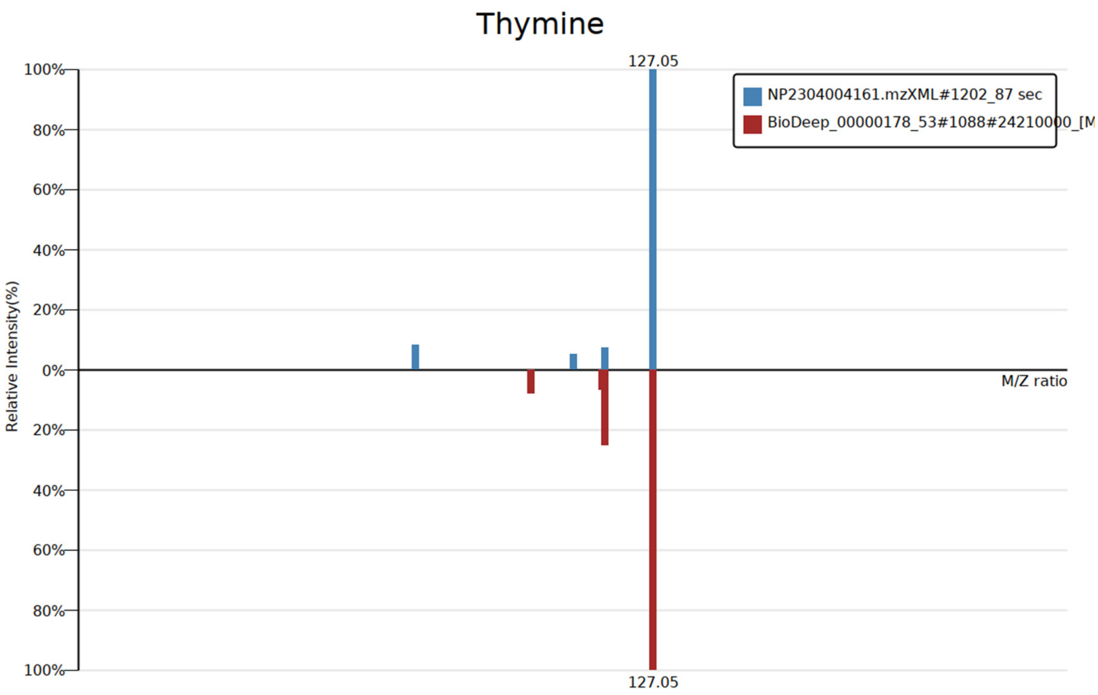

3-Methyladenine

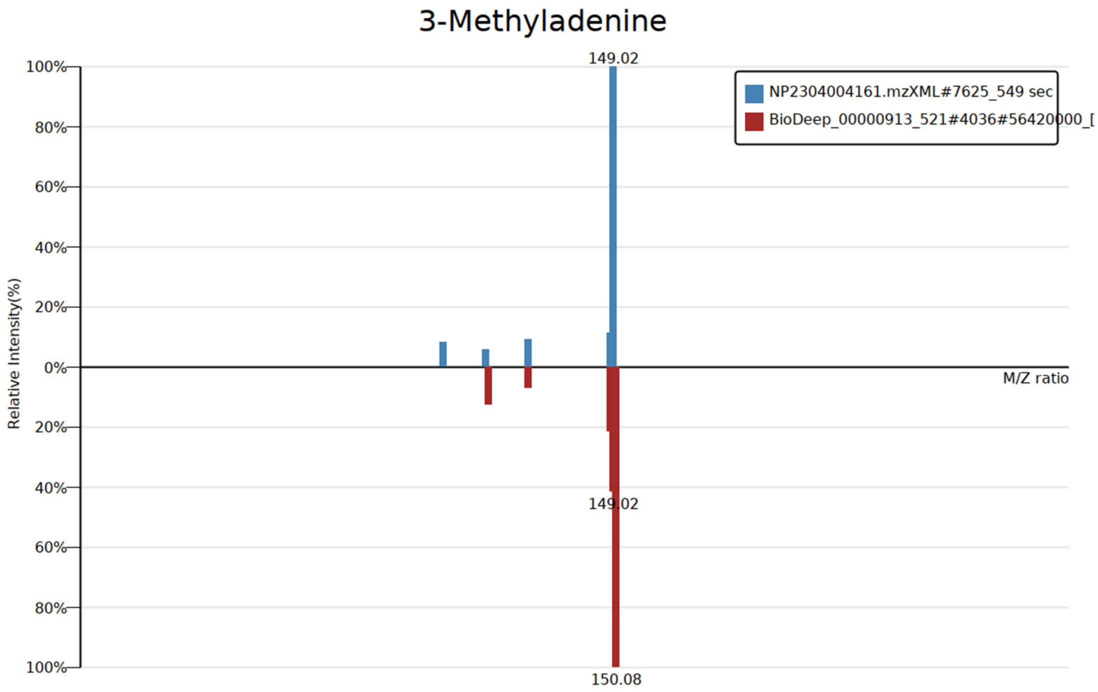

Guanine

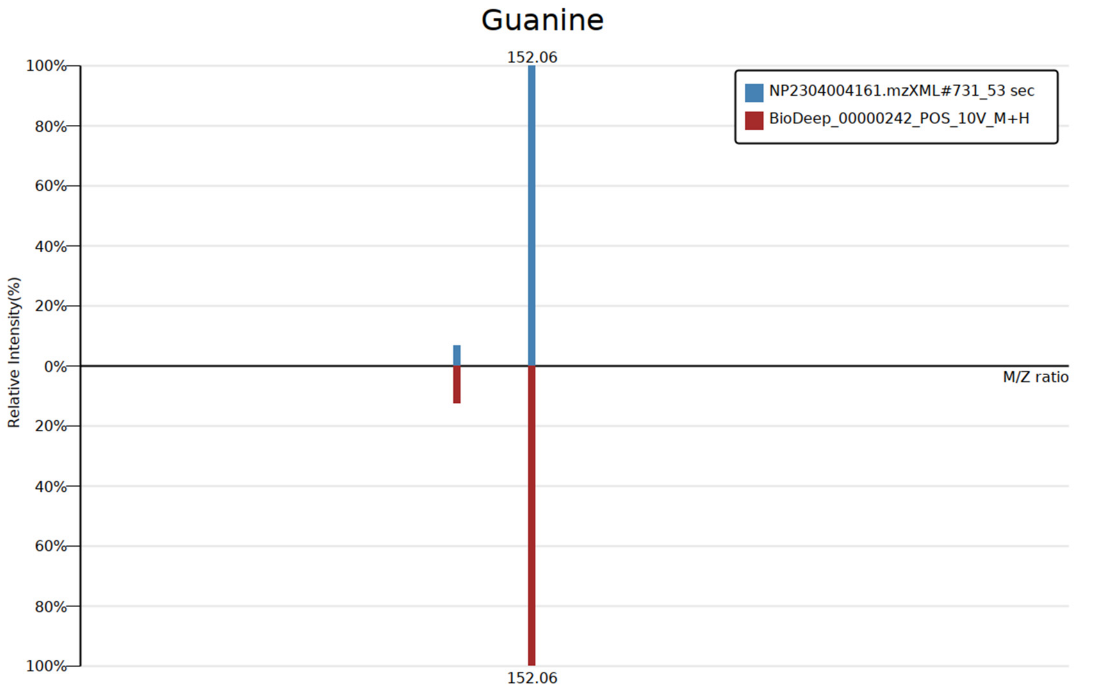

Cytidine

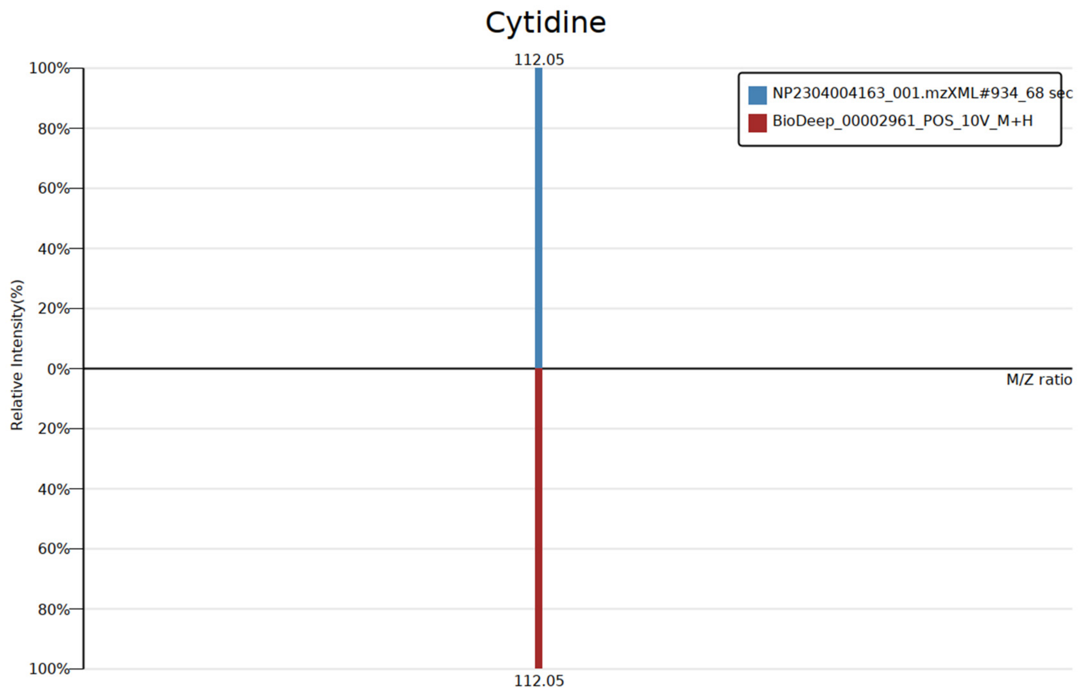

Uracil

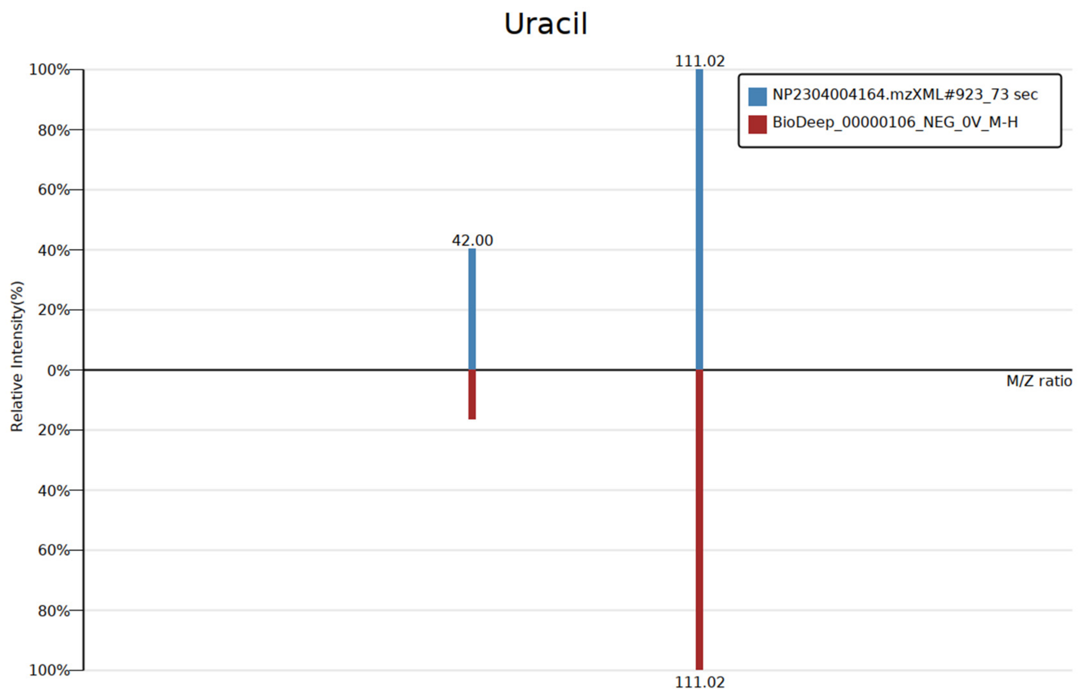

Adenine

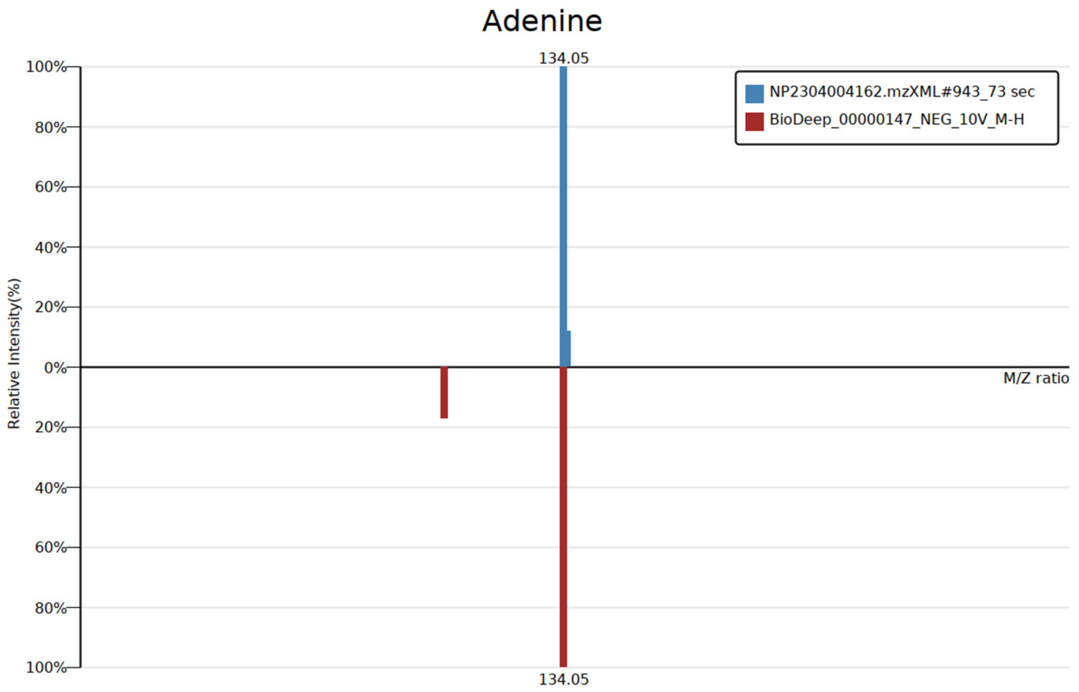

Cyclic AMP

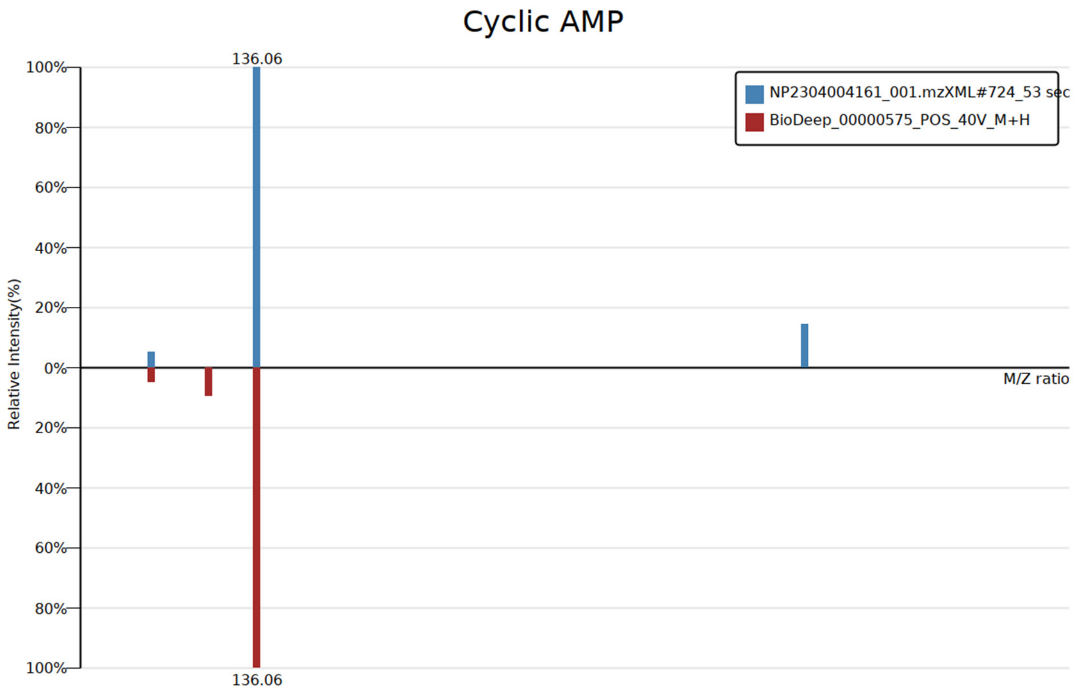

UMP

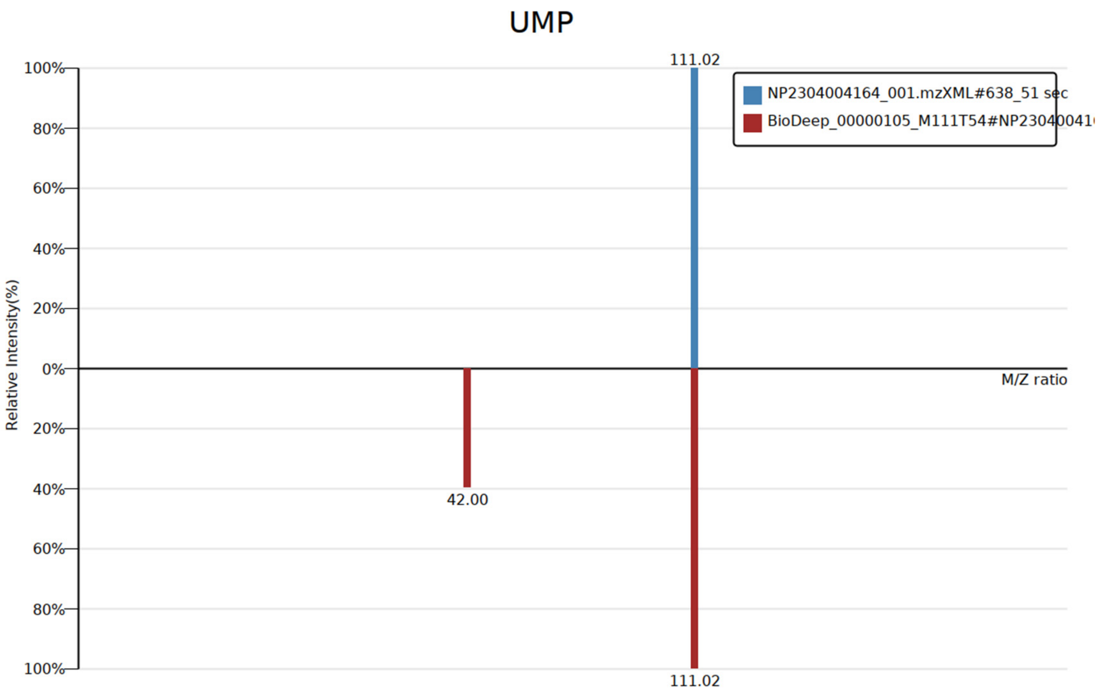

D-Xylitol

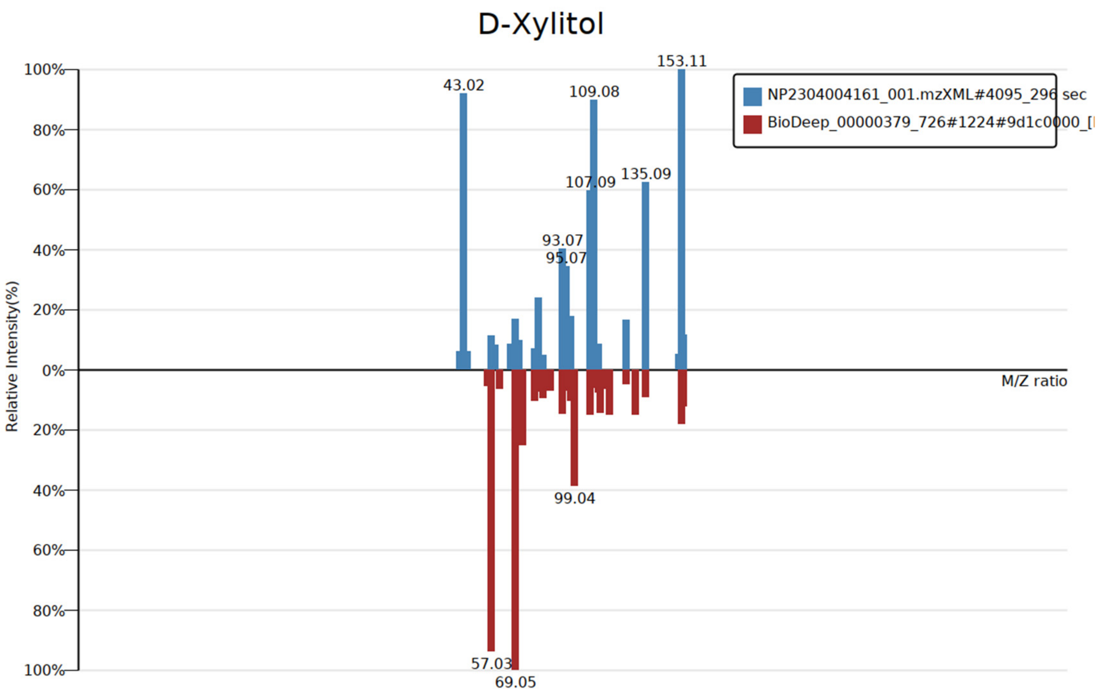

Sorbitol

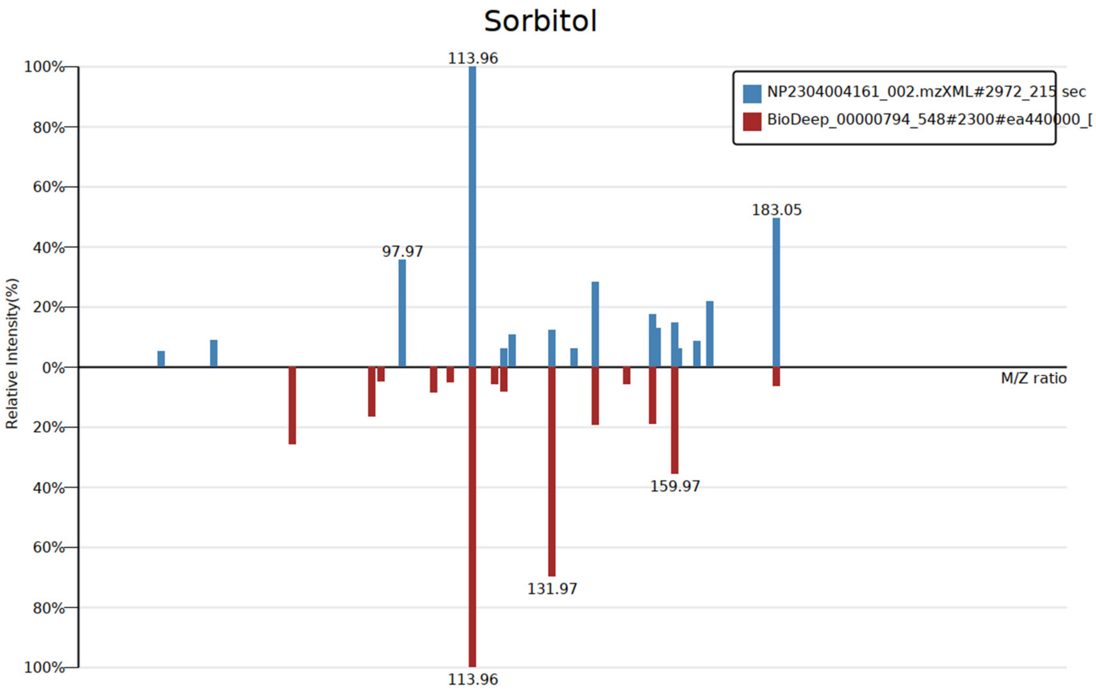

Ribitol

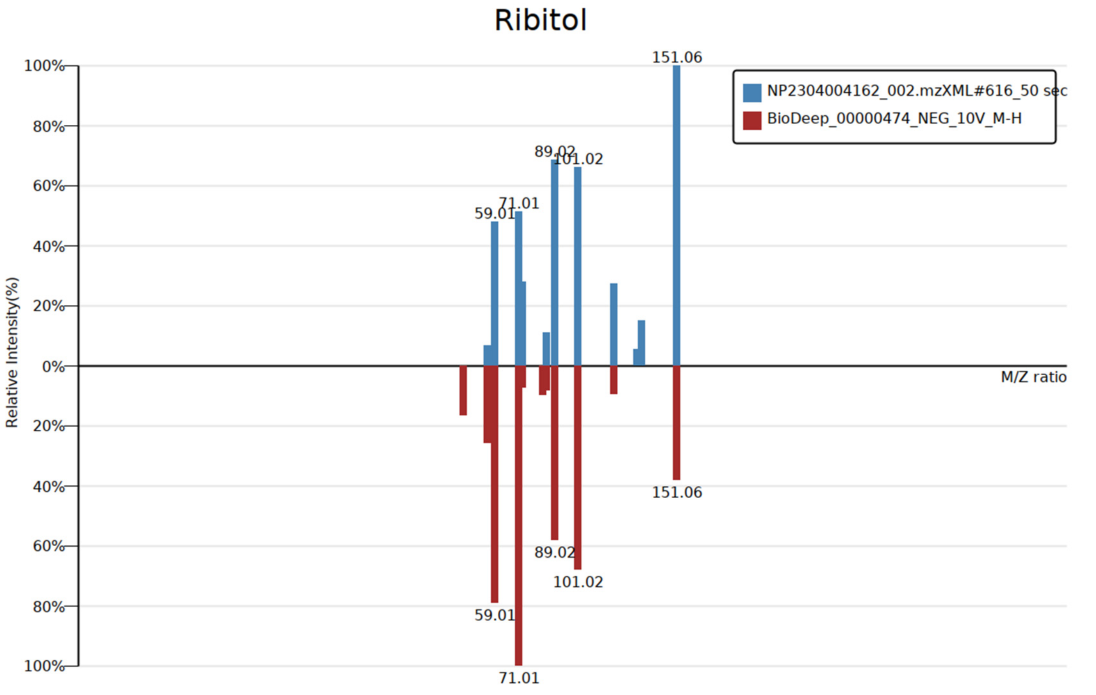

D-Fructose

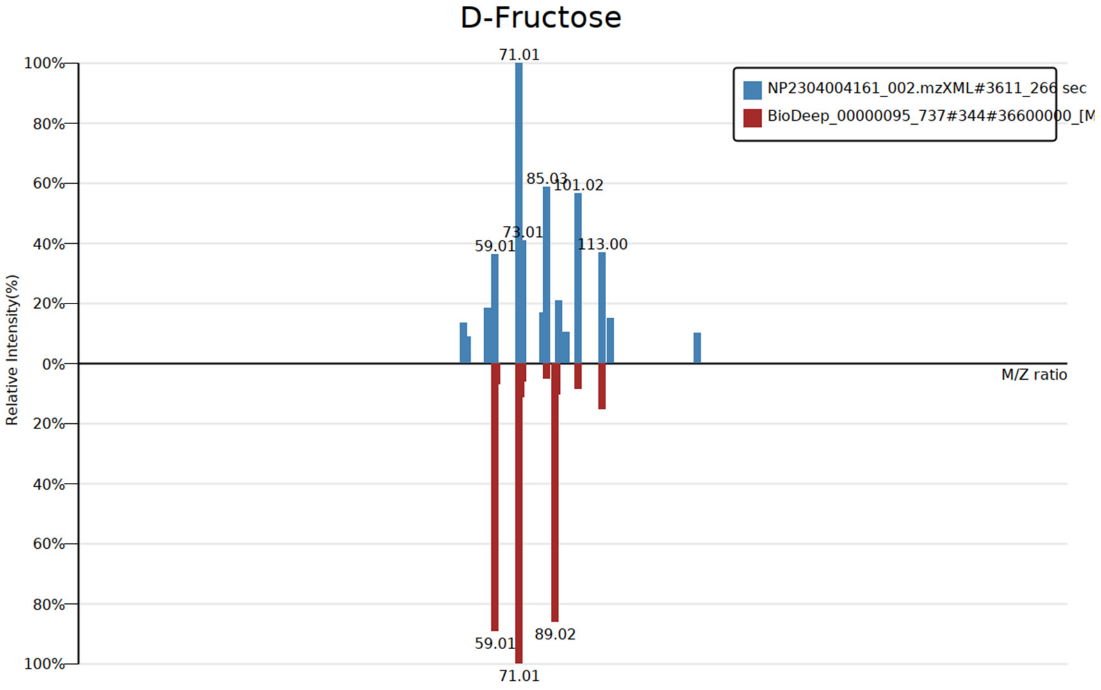

Fructose-1P

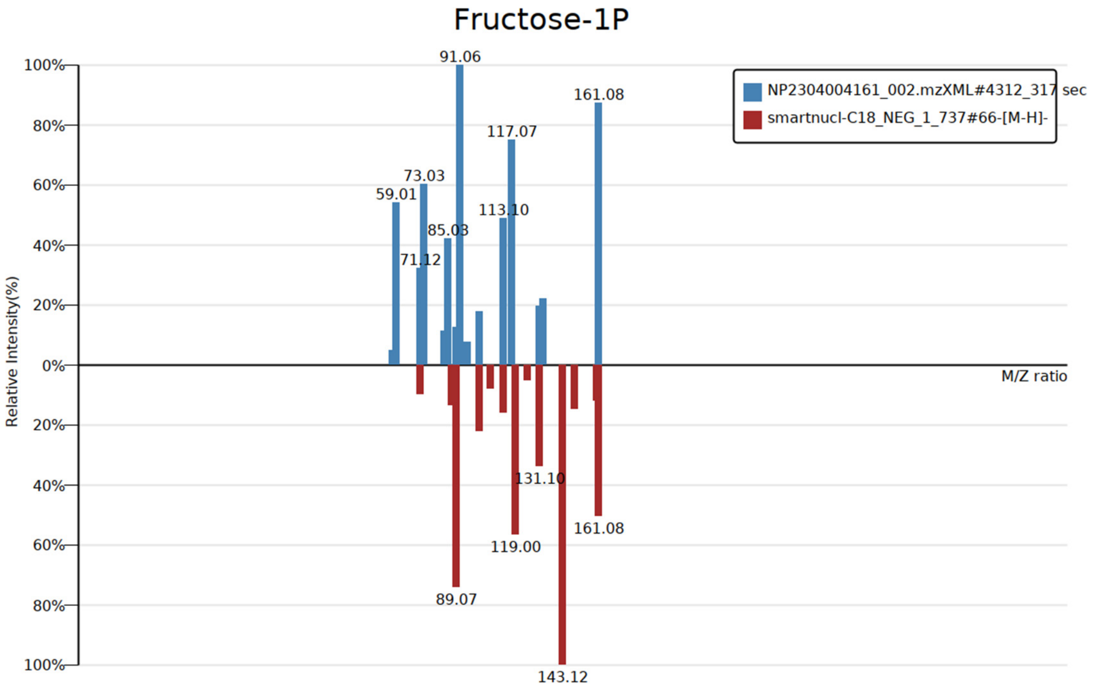

myo-Inositol

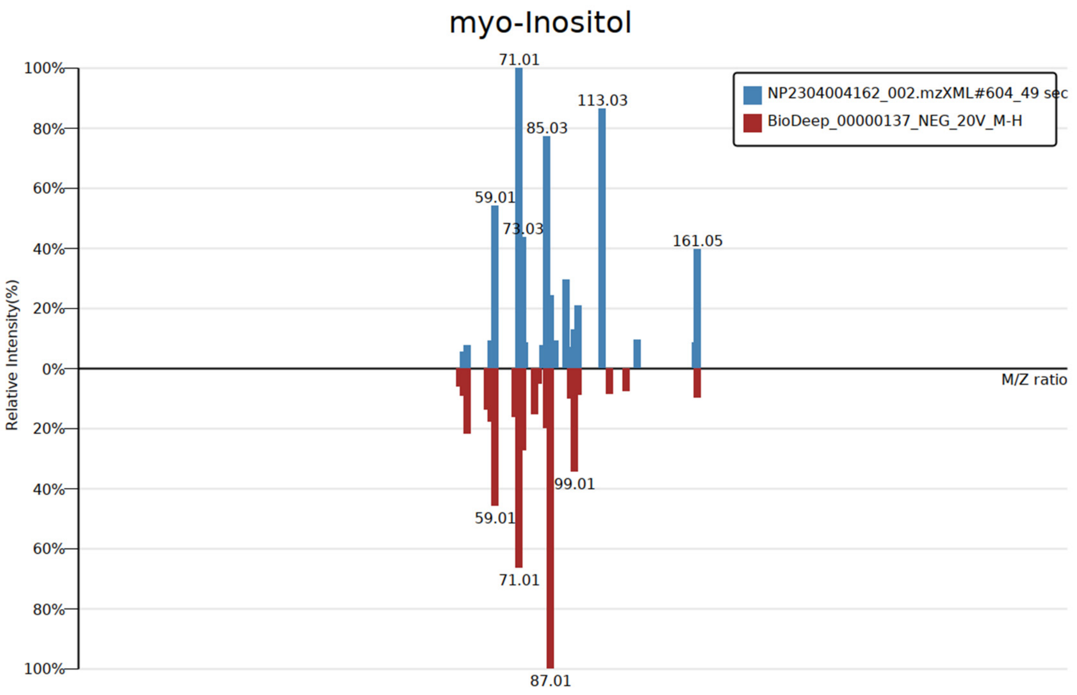

(+)-Demethoxyaschantin

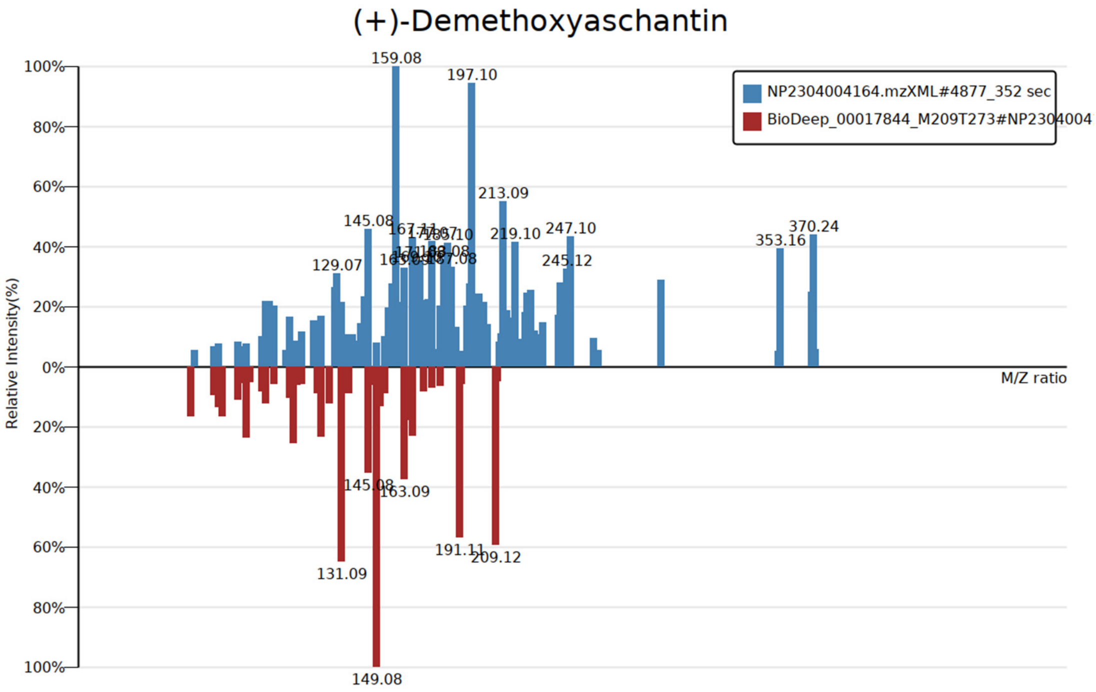

Coumarin

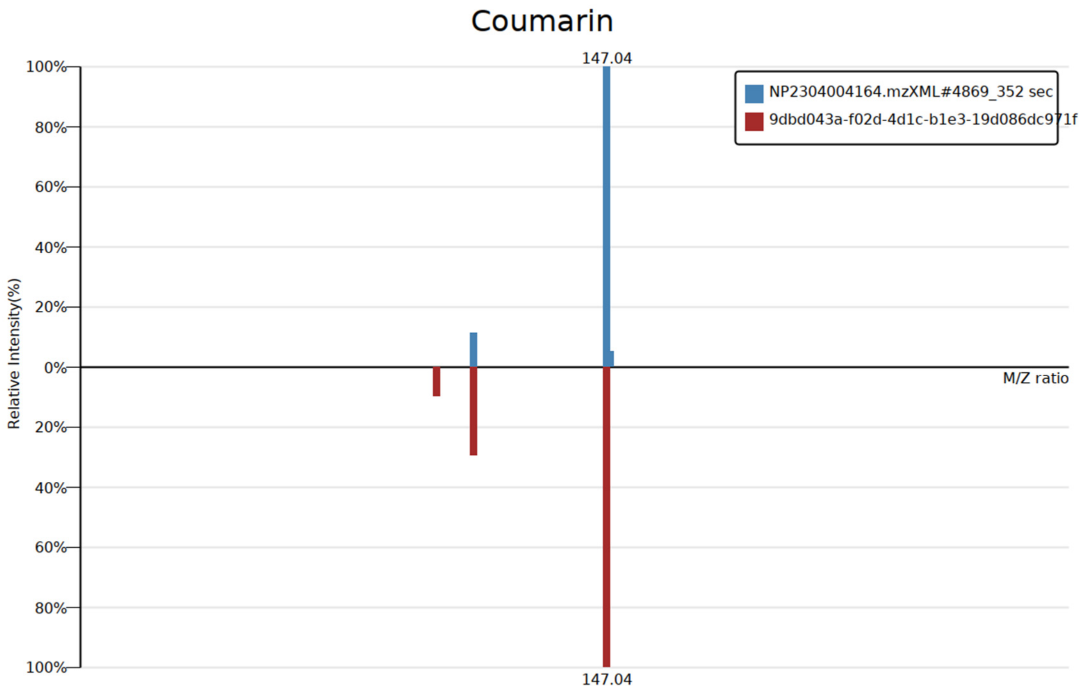

4-Hydroxycoumarin

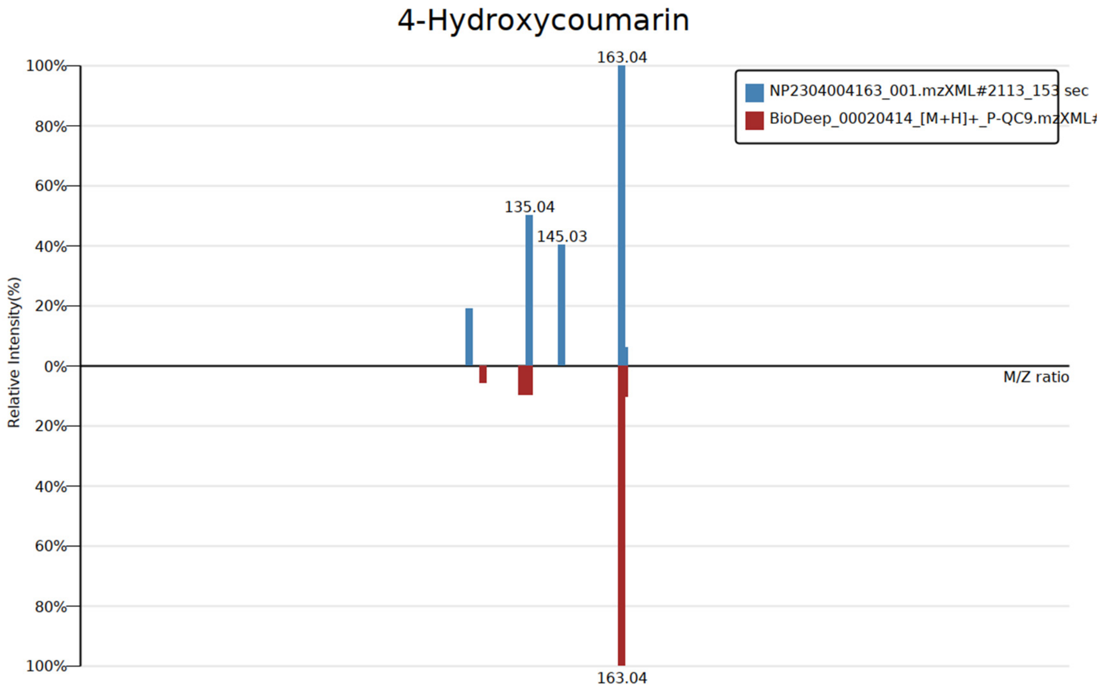

Neocnidilide

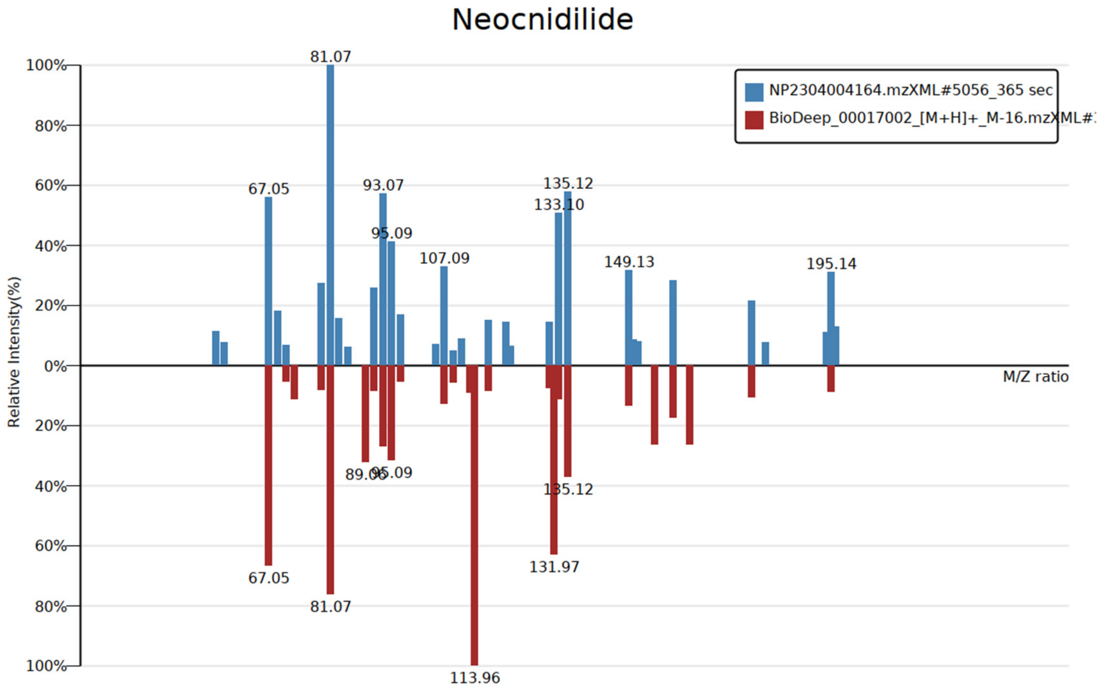

Anabasine

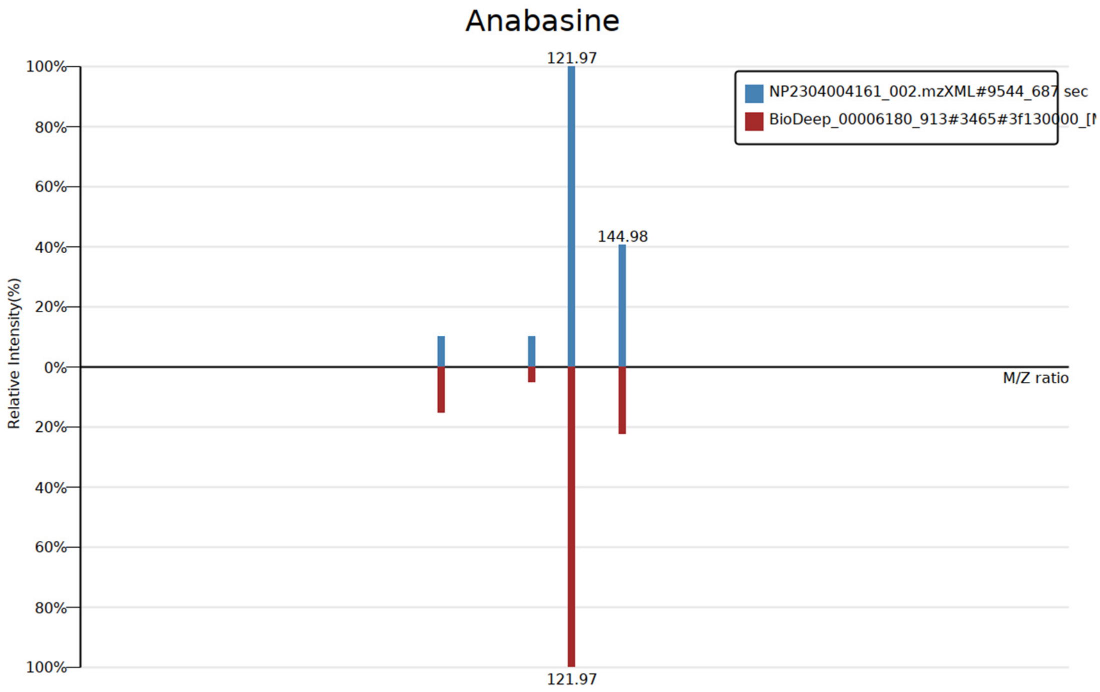

17-O-Acetylnorajmaline

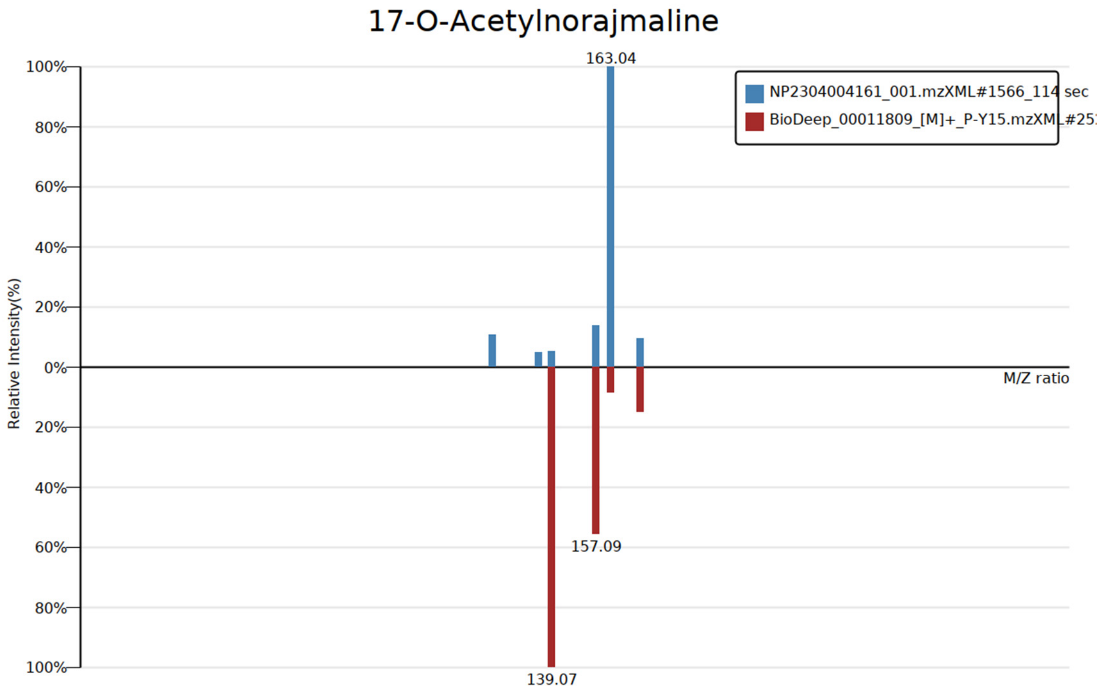

Isoandrocymbine

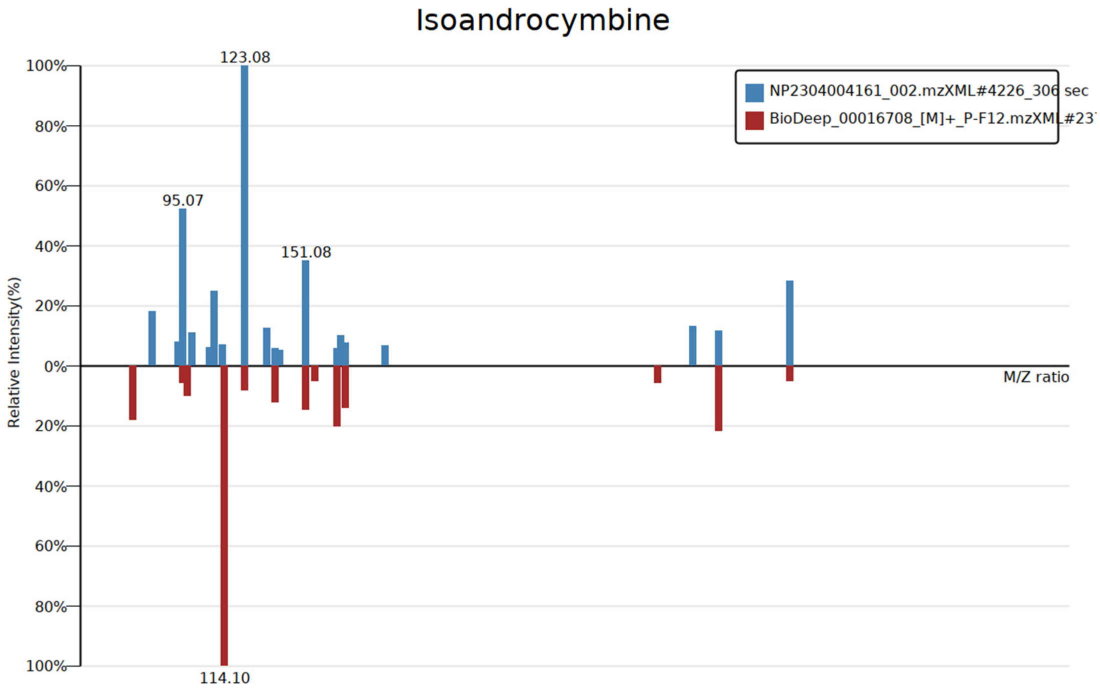

Xanthoxic acid

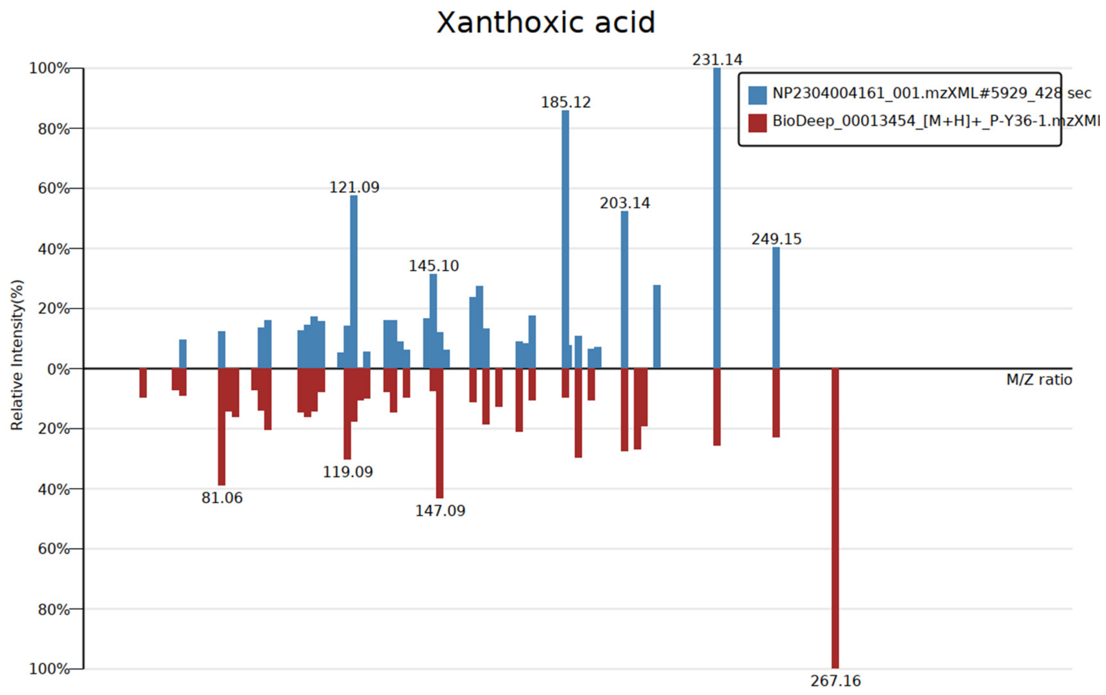

Xanthoxin

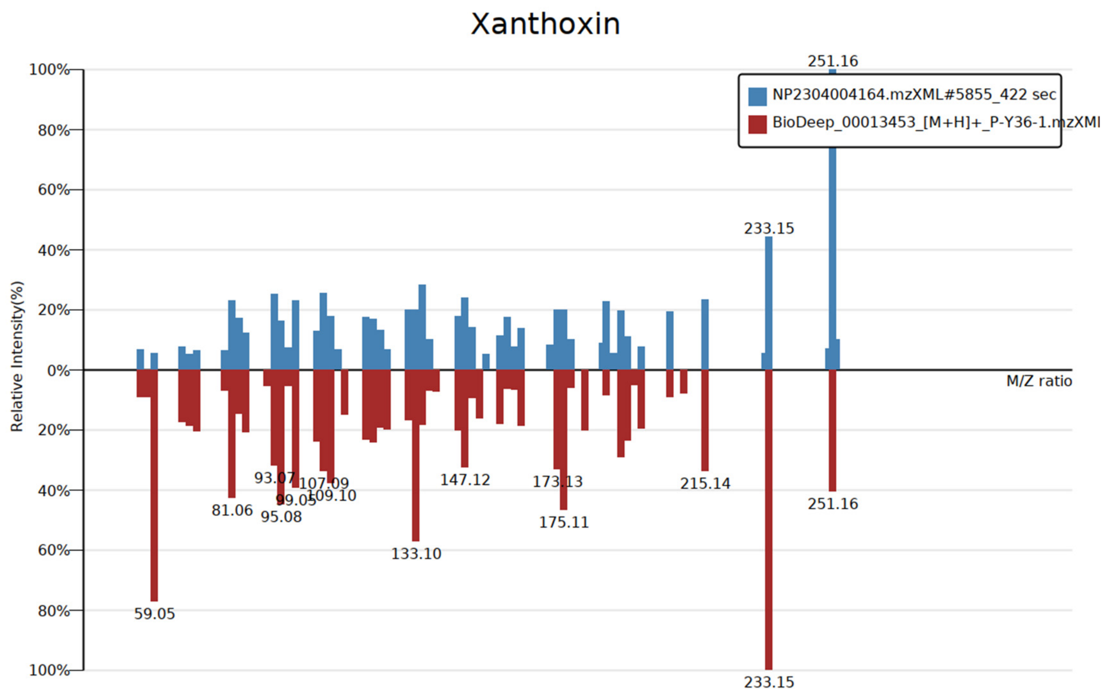

Methyl cinnamate

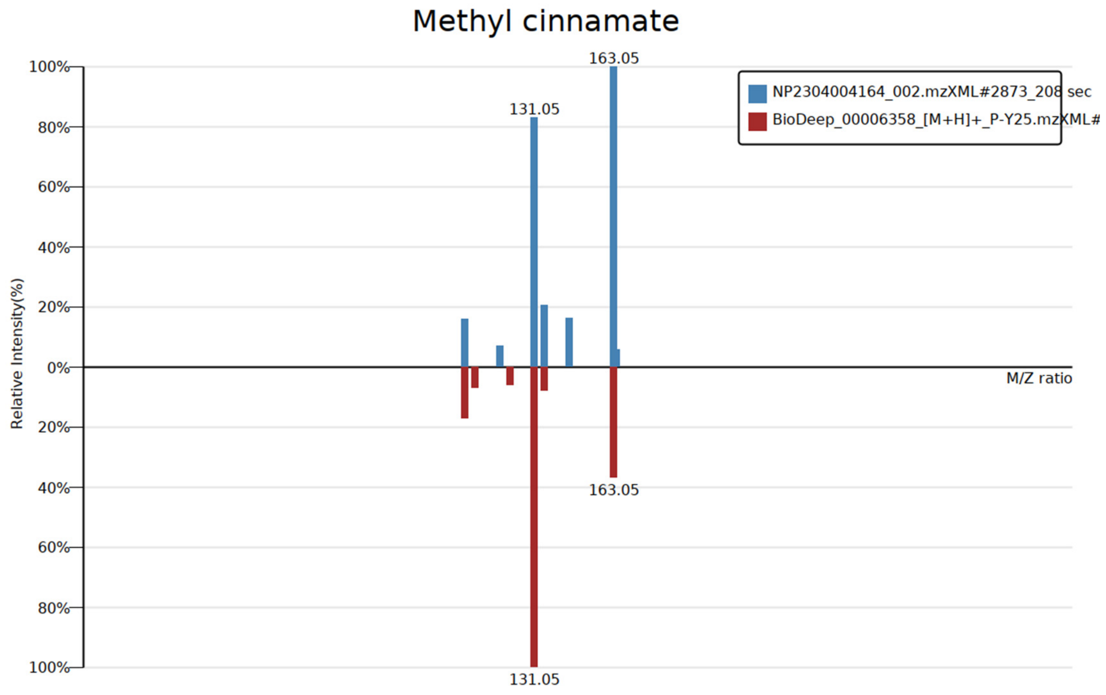

N-Butyryl-L-homoserine lactone

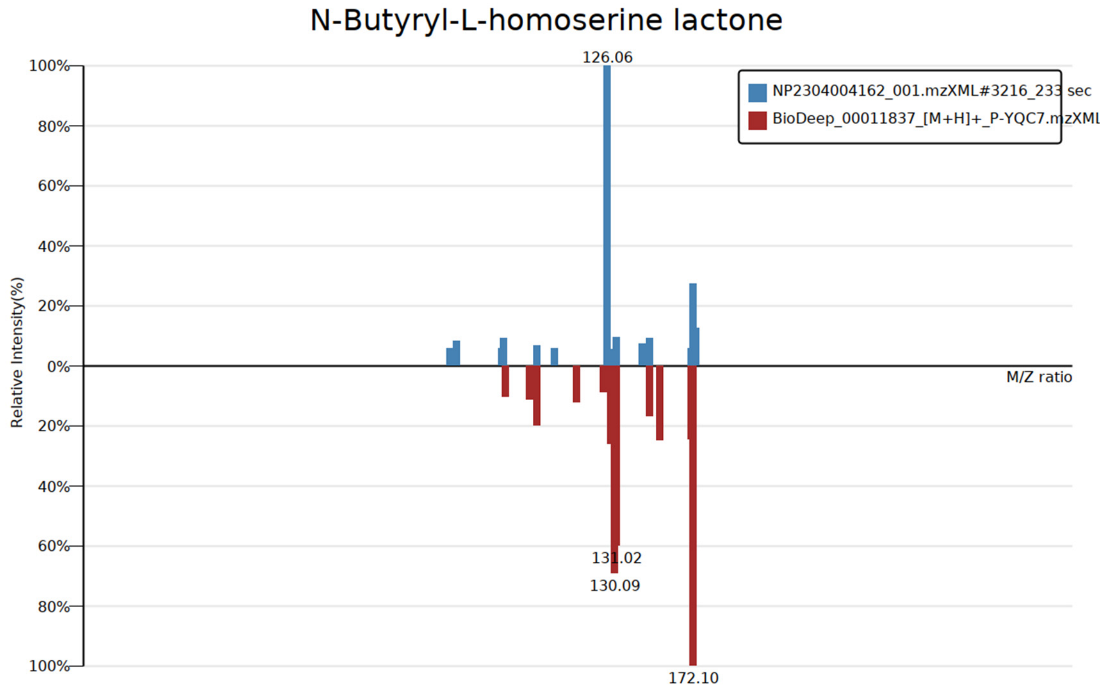

Demethylated antipyrine

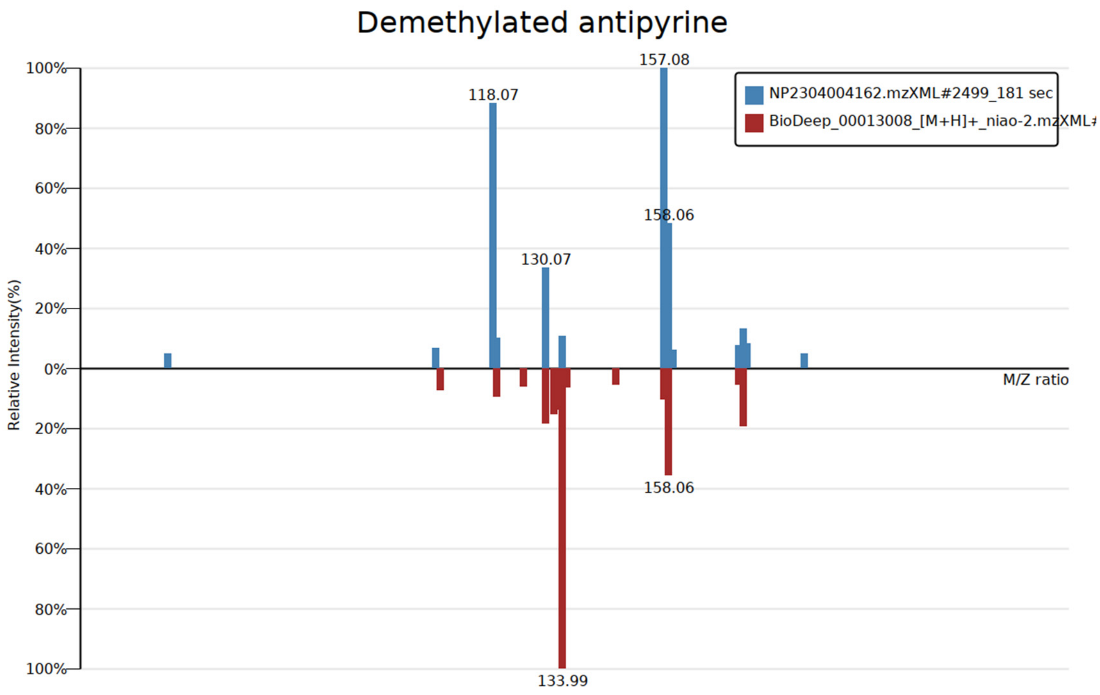

Methyleugenol

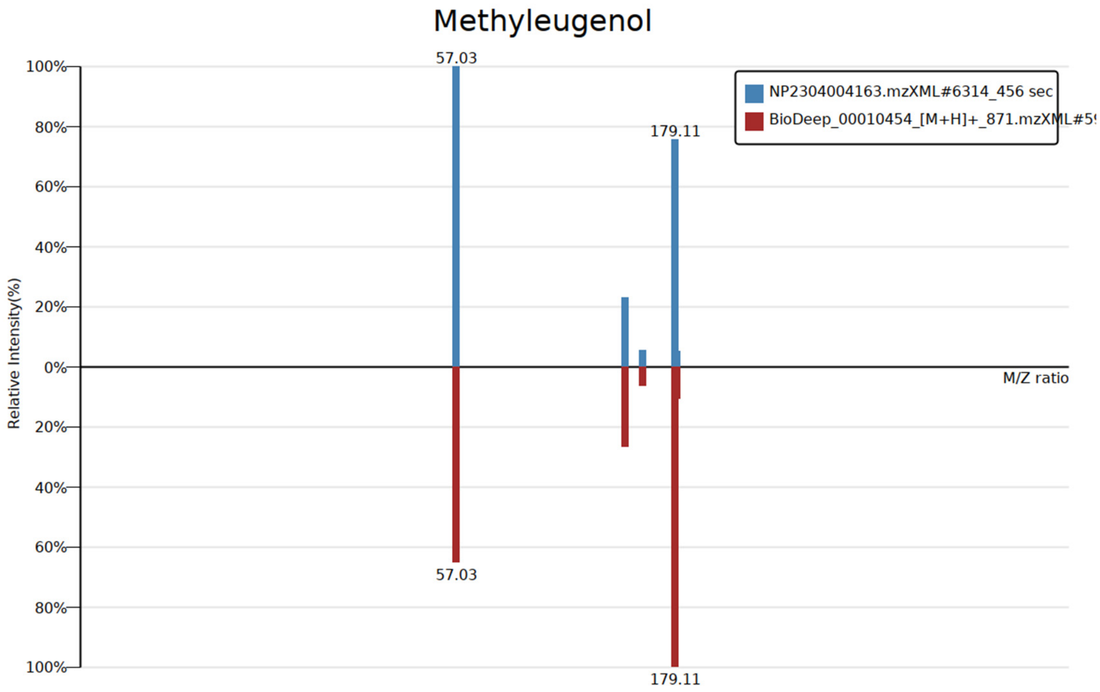

Phenylethylamine

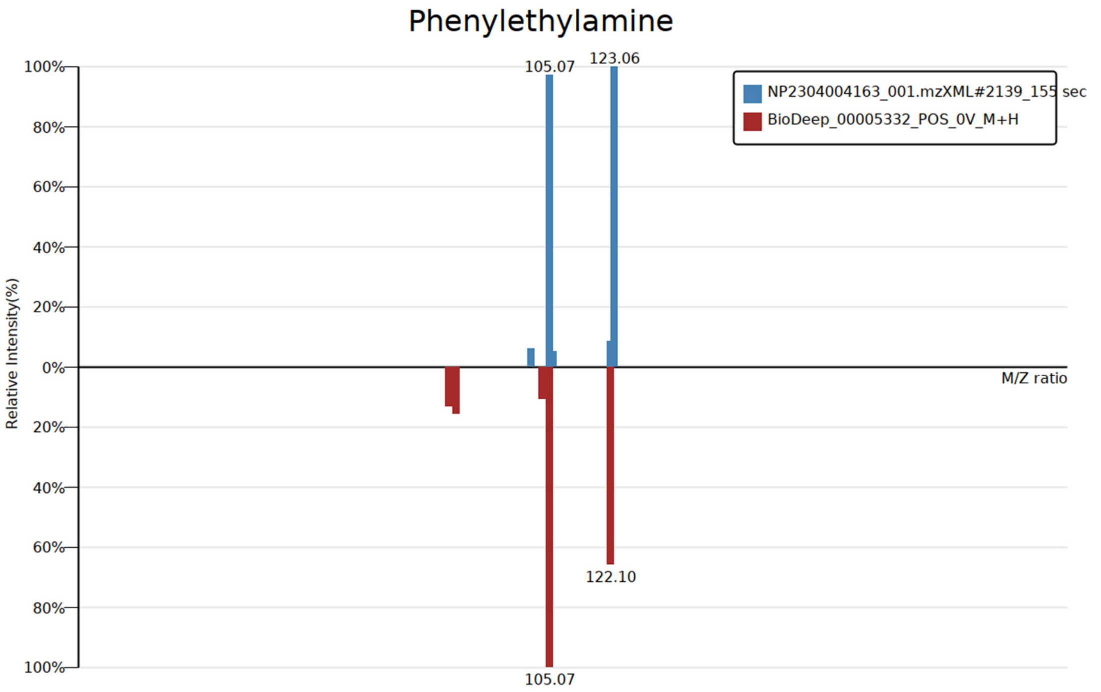

Benzaldehyde

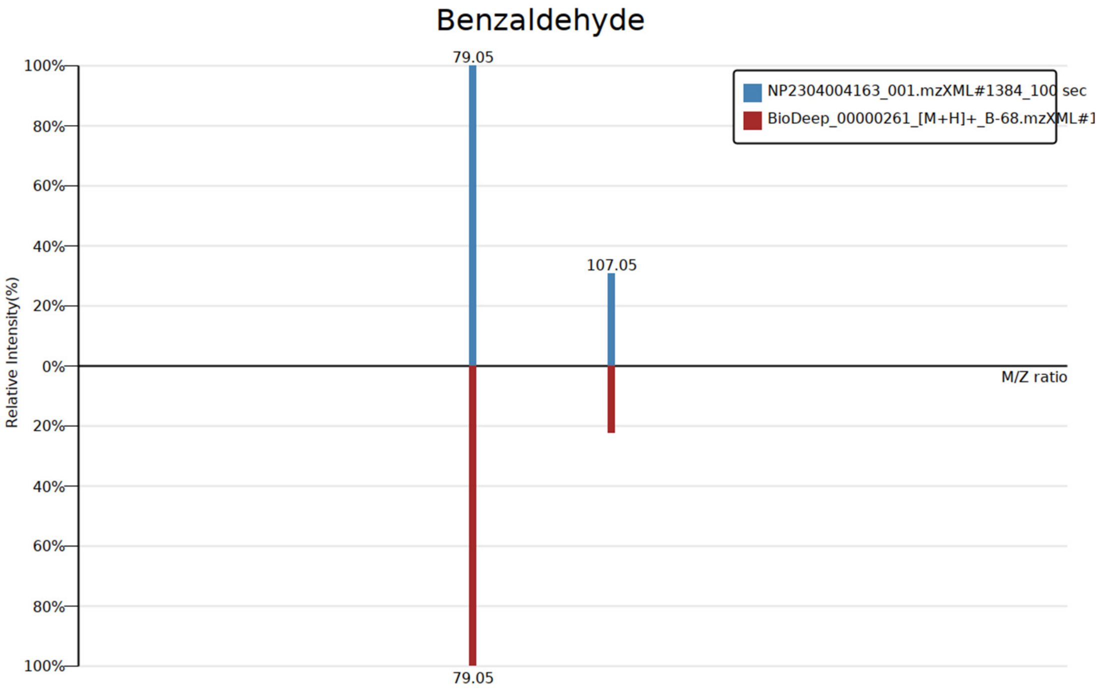

Indolin-2-one

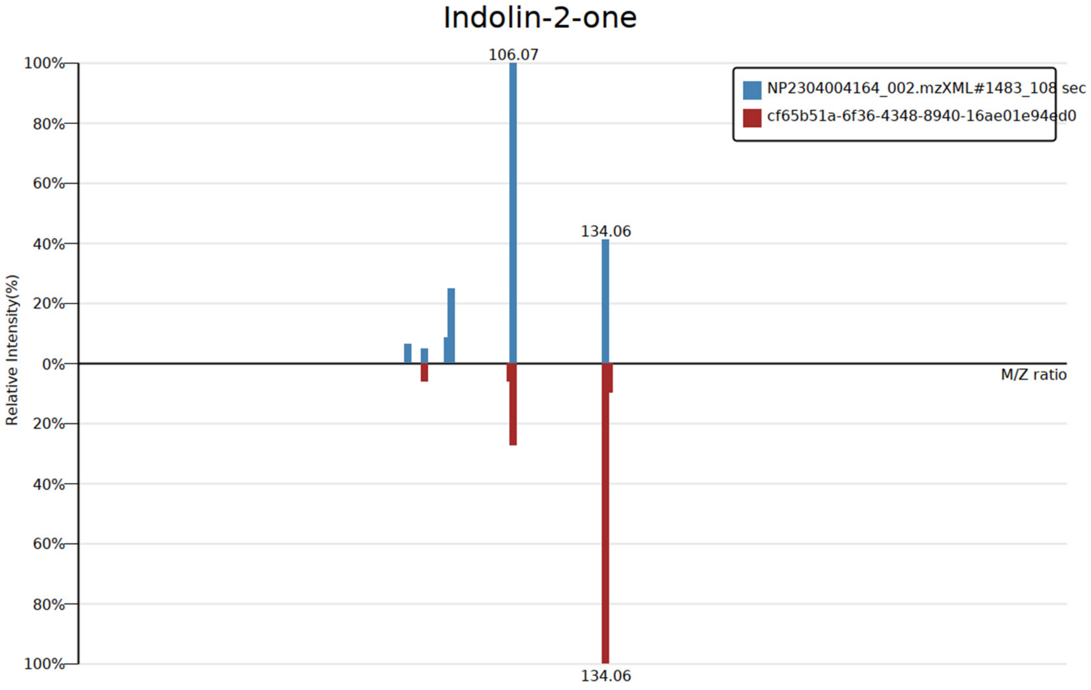

2,6-Dimethyl-naphtalene

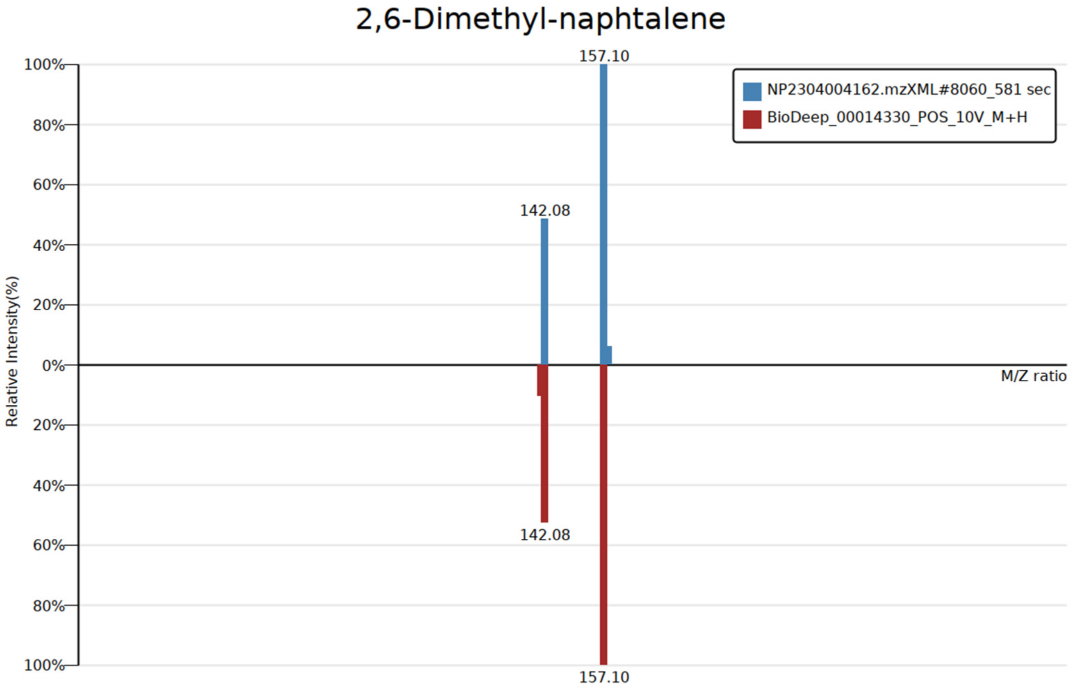

Methacholine

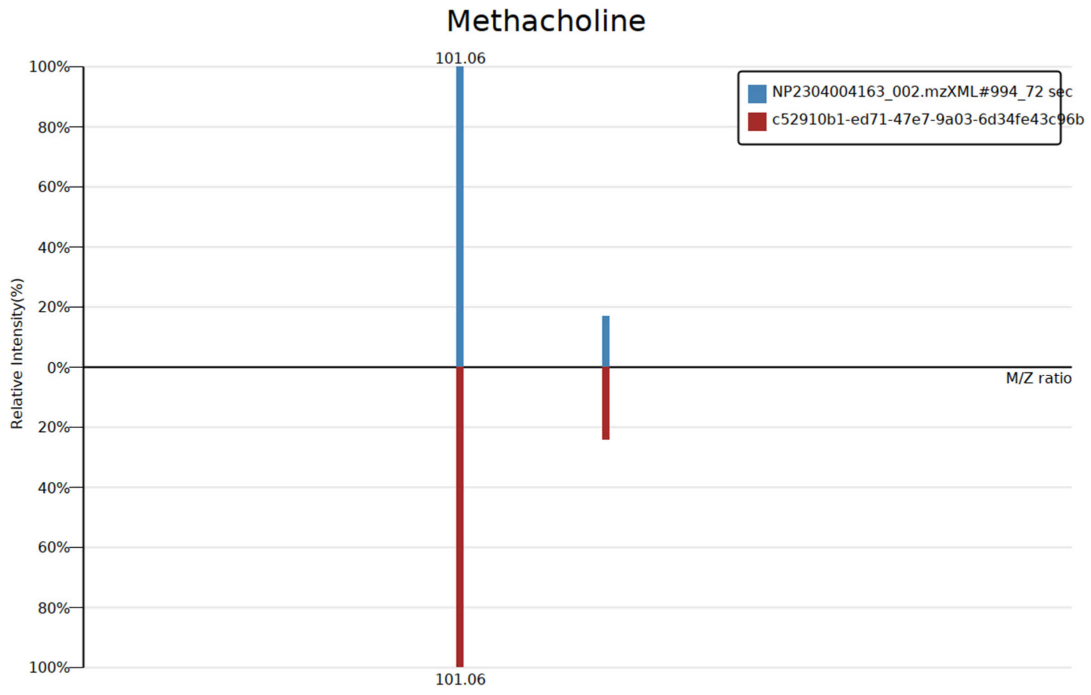

1,4-Dimethyl-7-ethylazulene

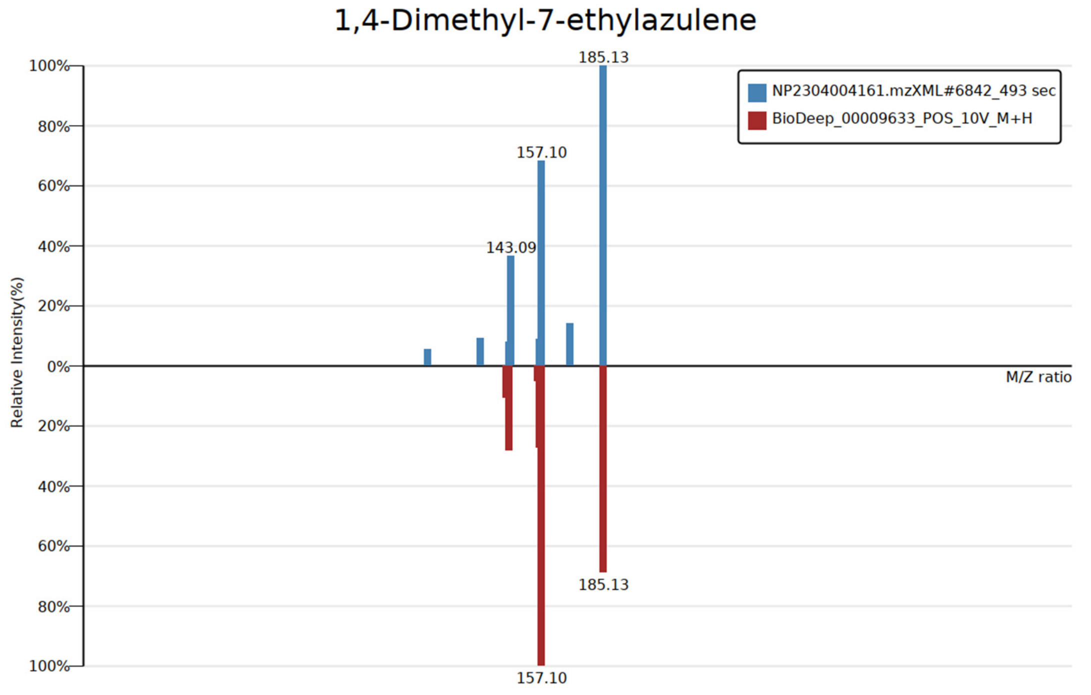

Carnosine

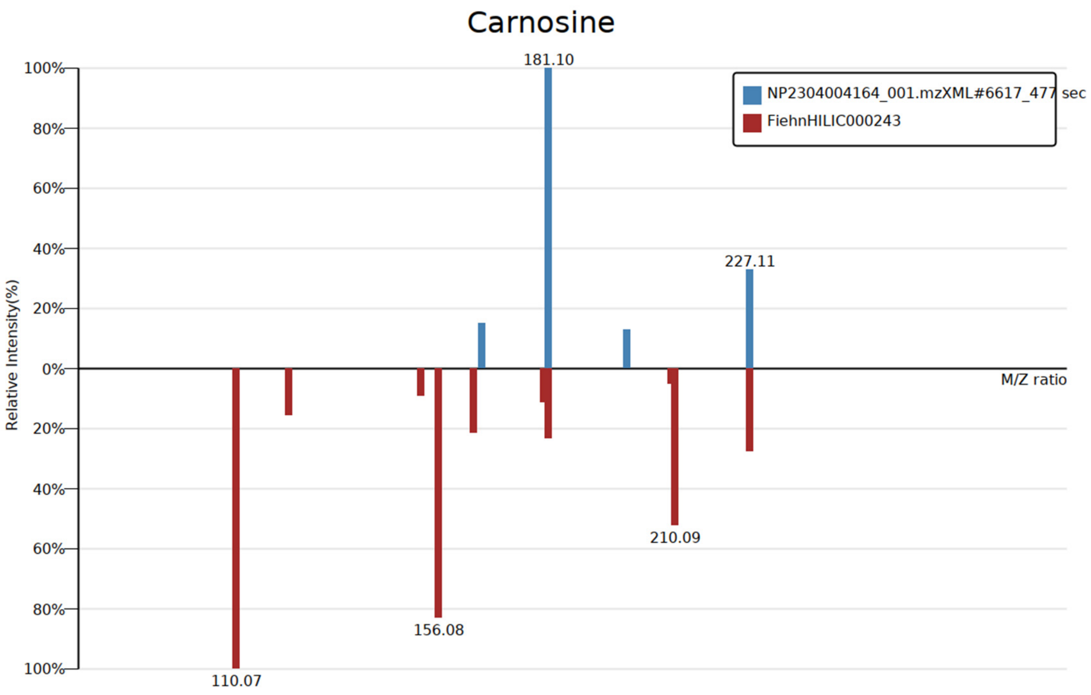

Glycerophosphocholine

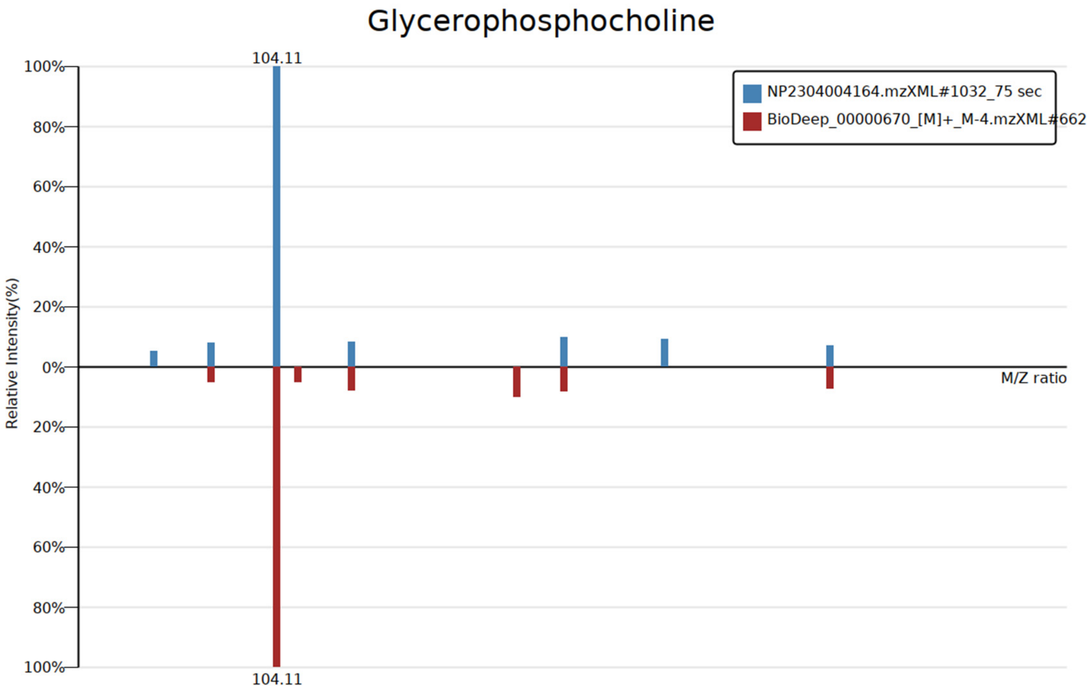

1,3-Benzenediol

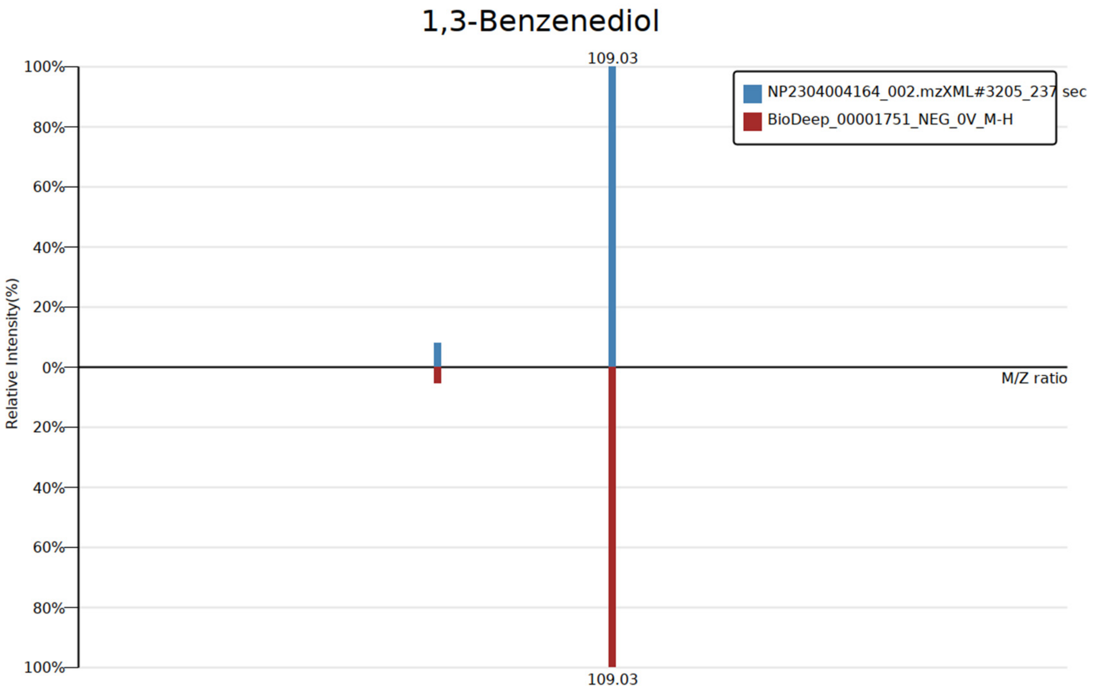

Acetylphosphate

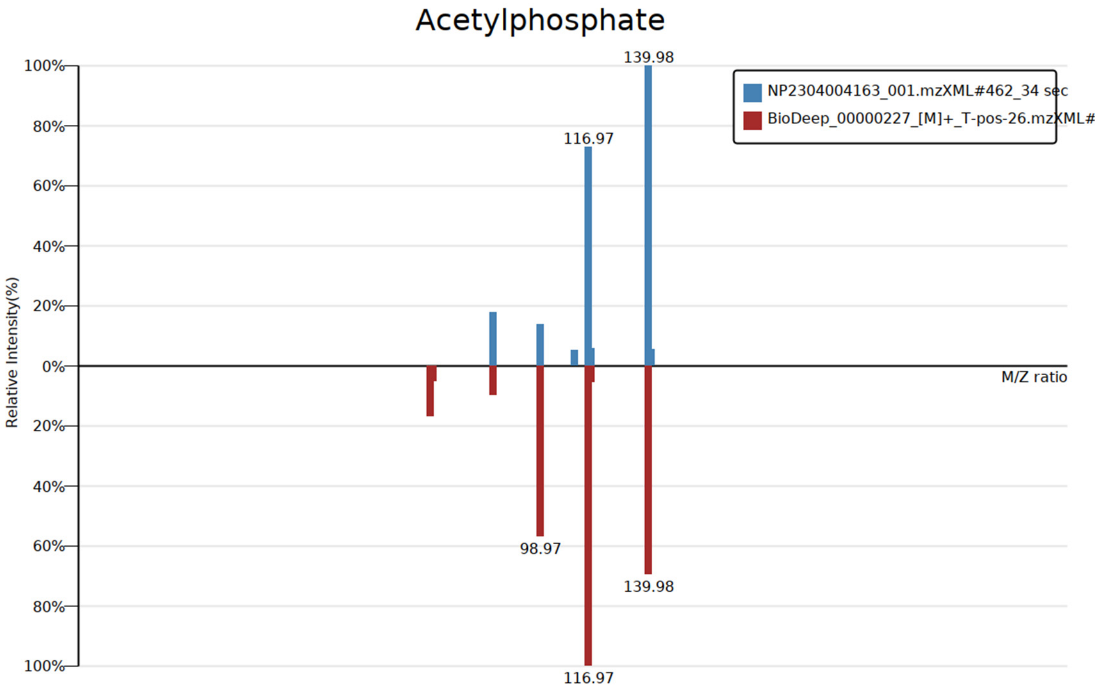

Kinetin

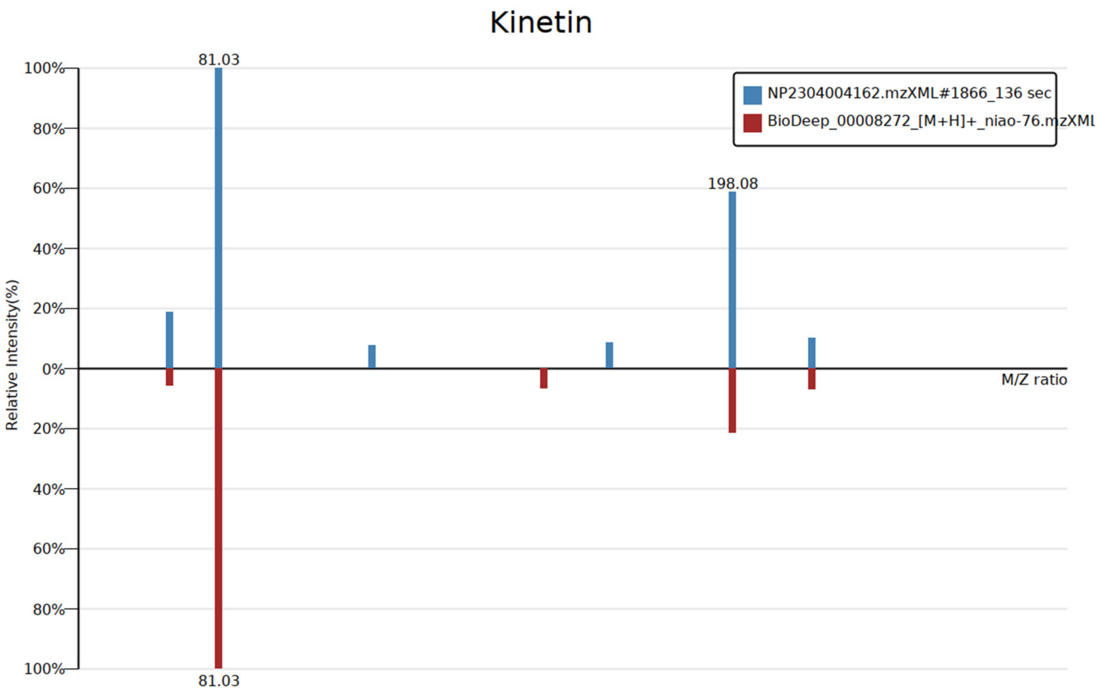

Kinetin
NP2304004162.mzXML#1866_136 sec
BioDeep_00008272_[M+H]+_niao-76.mzXML
100%
80%
60%
40%
20%
0%
20%
40%
60%
80%
100%
Relative Intensity(%)
81.03
198.08
M/Z ratio
81.03
