# Supplementary material for: A Comprehensive Metabolomic Analysis of Volatile and Non-Volatile Compounds in Folium Artemisia argyi Tea from Different Harvest Times
Source: Foods. 2025 Feb 28;14(5):843. doi: 10.3390/foods14050843 (PMC11899400; doi:10.3390/foods14050843)

Characterization of the chemical structures of key compounds and chromatograms in FAA tea harvested at four dif-ferent times

1. 1-Octen-3-one

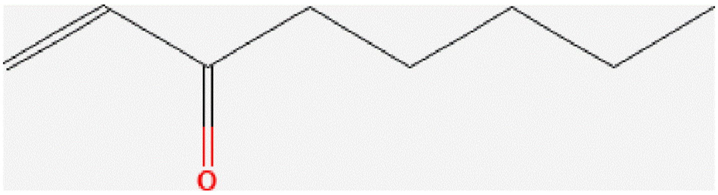

2. (E)-2-octenal

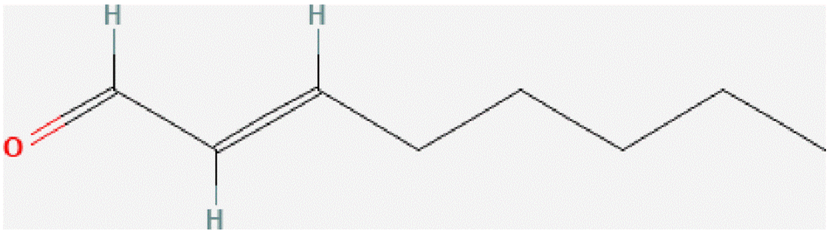

3. (E)-2-undecenal

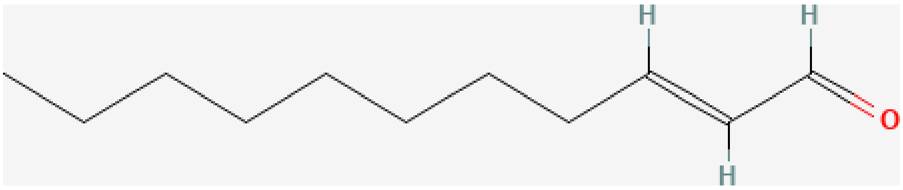

4. Heptanal

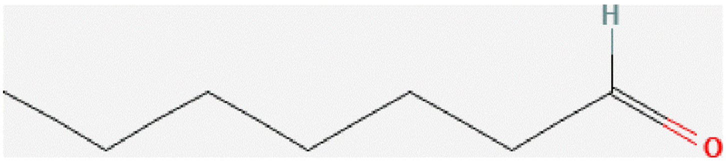

5. D-xylitol

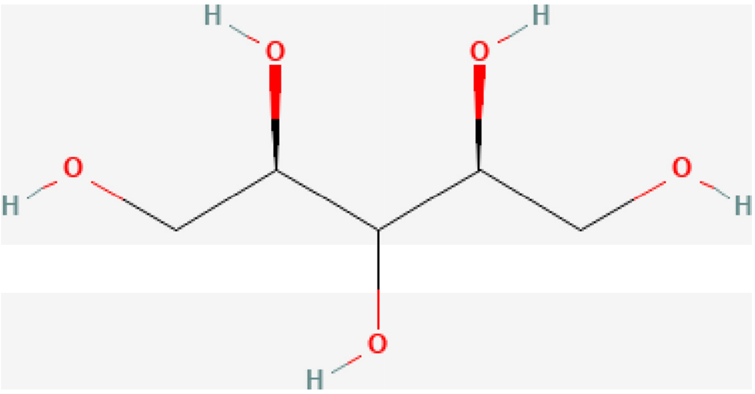

6. L-glutamic acid

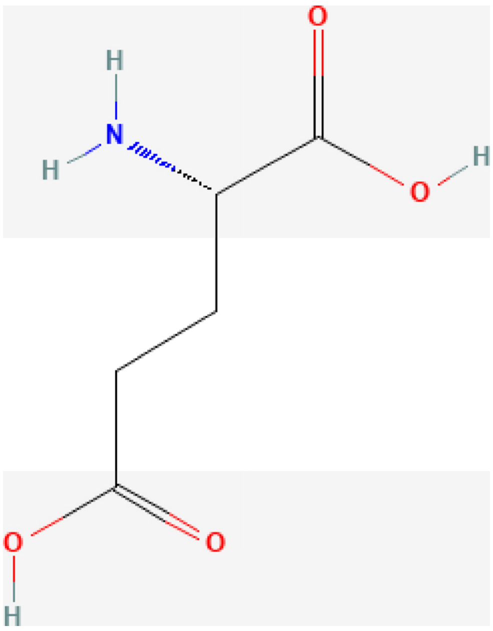

7. Honokiol

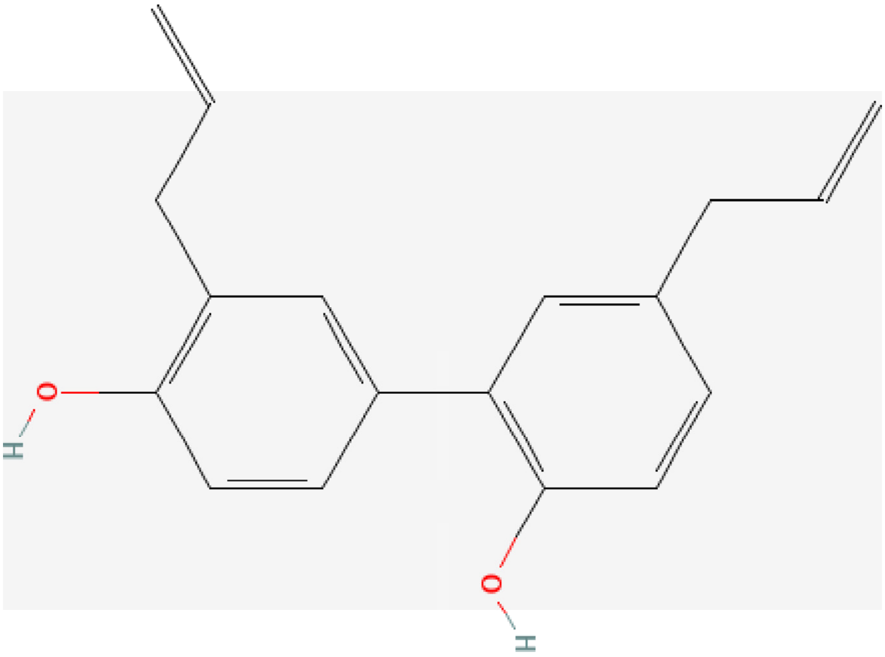

8. Costunolide

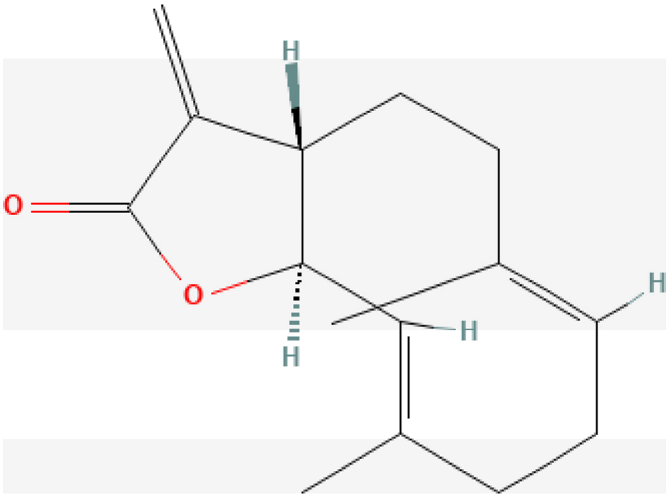

9. kynurenic acid

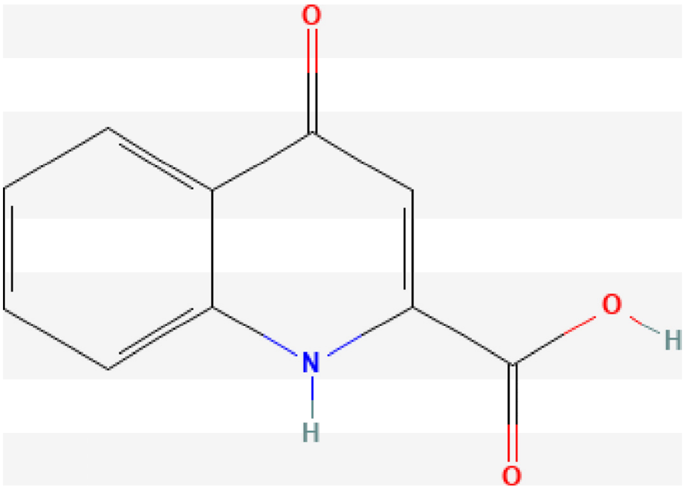

Supplement: Supplementary file 1 [file foods-14-00843-s001.zip › Figure S3 Characterization of the chemical structures of key compounds and chromatograms in FAA tea harvested at four dif-ferent times.pdf]
