# Supplementary material for: A Comprehensive Metabolomic Analysis of Volatile and Non-Volatile Compounds in Folium Artemisia argyi Tea from Different Harvest Times
Source: Foods. 2025 Feb 28;14(5):843. doi: 10.3390/foods14050843 (PMC11899400; doi:10.3390/foods14050843)
Supplement: Supplementary file 1 [file foods-14-00843-s001.zip › Table S1 The volatile compounds with ROAV values greater than one in four FAA tea harvest times.pdf]

Table S1 The volatile compounds with ROAV values greater than one in four FAA tea harvest times

Name	Class	Thresholds	Odor Character	ROAV			
				FAA-3	FAA-4	FAA-5	FAA-6
1-Octen-3-ol	Alcohols	11	Mushroom	1.3	1.82	1.2	22.62
1-Pentanol	Alcohols	5.5	wine	0.37	0.97	0.8	4
1-Hexanol	Alcohols	2.4	green grass, plastic	0.12	0.74	0.53	11.45
(E)-2-Octenal	Aldehydes	0.003	Nuts, Green, Fatty	934.52	1480.85	870.32	6349.64
(E)-2-Nonenal	Aldehydes	0.0002	Fatty, Cucumber	710.96	882.28	1029.27	100
Heptanal	Aldehydes	0.003	Citrus, Fatty, Rancid	103.33	269.33	139.9	1394.18
Vanillin	Aldehydes	0.0002	vanilla, caramel, sweet	100	100	100	100
(2E,6Z)-Nonadienal	Aldehydes	0.02	Cucumber, Green	53.52	47.44	1	1
Benzaldehyde	Aldehydes	1.5	bitter almond, fruity, vanilla	48.89	74.71	39.79	483.07
Benzeneacetaldehyde	Aldehydes	1	Floral, Honey	36.31	67.51	31.23	37.59
(E)-2-Undecenal	Aldehydes	0.0004	Fatty, Waxy, Rose, Citrus	35.17	50	342.44	1535.34
α-Terpineol	Aldehydes	1	Piney, Iris, Teil	7.25	17.73	15.74	5.23
Nonanal	Aldehydes	1	Aldehyde, Citrus, Orange Peel	1.16	1.16	0.88	0.02
Citral	Aldehydes	0.024	lemon, flowery, citrous	0.59	26.08	15.08	100.73
Octanal	Aldehydes	2.5	Lemon, Citrus, Green Grass	0.43	0.64	0.18	3.36
Hexanal	Aldehydes	20	Green Grass, Fruity	0.2	0.3	0.18	1.76
Styrene	Aromatic hydrocarbon	2.8	sharp, sweet	0.01	0.01	0.01	4.2
p-Cymene	Aromatic hydrocarbon	11.4	woody, herbal	0.14	4.64	2.17	0
Isovaleric acid	Carboxylic Acids	0.02	Rancid Cheese, Sweaty, Putrid	259.96	589.78	414.87	2072.69
Benzyl acetate	Esters	0.16	pears, plastic, etherous, anise	10.1	31.09	16.7	70.83
Ethyl palmitate	Esters	2	Wax	0.29	0.47	0.35	6.18
Phenethyl acetate	Esters	108	Rose, Honey	0.18	0.51	0.33	1.2
Phenol	Esters	4.5	medicinal, acid, ink, creosote, empyreumatic	0.17	0.31	0.2	3.86
Ethyl hexanoate	Esters	30	Fruity, Green Apple	0.07	0.14	0.11	1.81
Ethyl-2-methylbutyrate	Esters	1.6	Fruity	0.01	0.01	0.01	7.63
trimethyl-Pyrazine	Heterocyclics	0.023	Roasted Nuts, Cocoa, Peanuts	35.22	100.9	64.32	300.3
2-pentyl-Furan	Heterocyclics	0.006	Green Beans, Vegetable	8.19	110.29	18.25	3.33
1-Octen-3-one	Ketones	0.005	Mushroom-Like	1125.85	1722.07	1107.43	8573.02
2-Undecanone	Ketones	0.004355	Orange, Fresh, Green	21.2	47.34	23.17	127.5
Acetophenone	Ketones	0.24	sweet, almond, pungent, oranges, river water	18.97	37.15	25.82	130.85
Isophorone	Ketones	0.3	sharp	1.32	5.13	0.76	98.32
2-Heptanone	Ketones	0.75	sweet, mushroom	0.56	1.21	1	6.95
Ethyl isobutyrate	Phenols	4.5	Fruity, Strawberry	0.01	0.12	0.4	1.38
α-Pinene	Terpenoids	0.06	turpentine, rosiny, pine tree, camphorous, firneedles	20.31	82.76	79.03	763.11
β-Ionone	Terpenoids	8.4	Flowery, Violet-Like	0.05	0.92	0	6.78
Camphor	Terpenoids	2.6	camphorous	0.01	0.01	0.01	109.17
