# Supplementary figures and images for: A Comprehensive Metabolomic Analysis of Volatile and Non-Volatile Compounds in Folium Artemisia argyi Tea from Different Harvest Times
Source: Foods. 2025 Feb 28;14(5):843. doi: 10.3390/foods14050843 (PMC11899400; doi:10.3390/foods14050843)

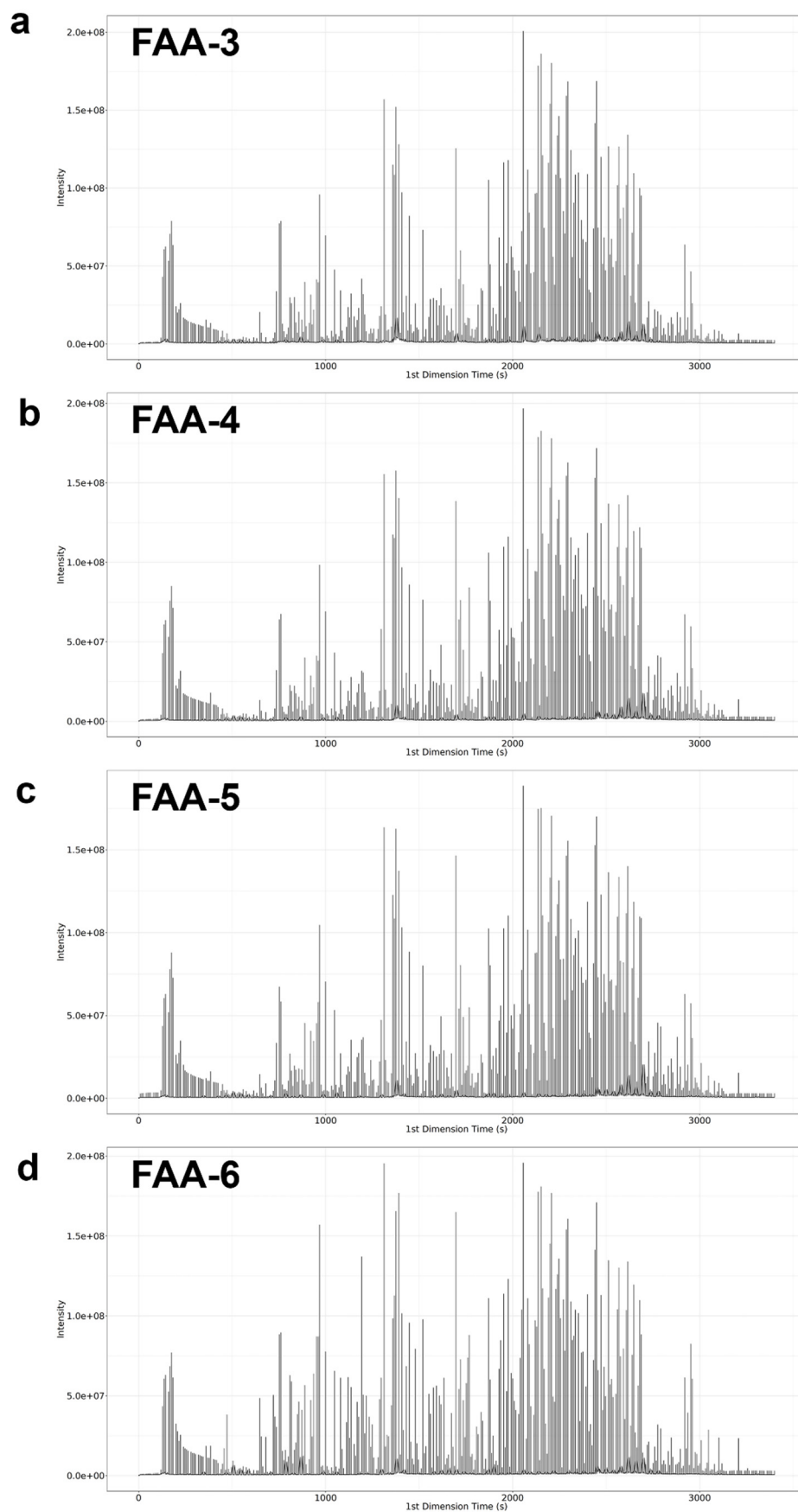

Figure S4 Chromatograms of FAA tea harvested at different times.

Supplement: Supplementary file 1 [file foods-14-00843-s001.zip › Figure S4 Chromatograms of FAA tea harvested at different times.pdf]
